# Candidate epitopes for measurement of hCG and related molecules: the second ISOBM TD-7 workshop

**DOI:** 10.1007/s13277-013-0994-6

**Published:** 2013-09-26

**Authors:** P. Berger, E. Paus, P. M. Hemken, C. Sturgeon, W. W. Stewart, J. P. Skinner, L. C. Harwick, S. C. Saldana, C. S. Ramsay, K. R. Rupprecht, K. H. Olsen, J.-M. Bidart, U.-H. Stenman, on behalf of the members of the ISOBM TD-7 Workshop on hCG and Related Molecules

**Affiliations:** 1 Institute for Biomedical Aging Research, University of Innsbruck, Rennweg 10, A6020 Innsbruck, Austria; 2 Department of Medical Biochemistry at Radiumhospitalet, Oslo University Hospital, Oslo, Norway; 3 Diagnostic Research and Development, Abbott Diagnostics, Abbott Park, IL USA; 4 Department of Clinical Biochemistry, Royal Infirmary, UK NEQAS for Peptide Hormones, Edinburgh, UK; 5 Department of Immunology, Ninewells Hospital and Medical School, Dundee, UK; 6 Department of Clinical Biology, Institut Gustave-Roussy, Villejuif, France; 7 Department of Clinical Chemistry, Helsinki University Central Hospital, Helsinki, Finland

**Keywords:** hCG variants measurement, Antibody standardization, Epitope standardization, International standards for hCG, hCG IRR

## Abstract

Participants of the Second International Workshop (WS) on human chorionic gonadotropin (hCG) of the International Society of Oncology and Biomarkers Tissue Differentiation 7 (ISOBM TD-7) have characterized in detail a panel of 69 antibodies (Abs) directed against hCG and hCG-related variants that were submitted by eight companies and research groups. Specificities of the Abs were determined using the First WHO International Reference Reagents for six hCG variants, i.e., hCG, hCGn, hCGβ, hCGβn, hCGβcf, and hCGα, which are calibrated in SI units, and hLH. Molecular epitope localizations were assigned to the ISOBM-mAbs by comparing ISOBM-Ab specificity, sandwich compatibility, and mutual inhibition profiles, to those of 17 reference monoclonal (m)Abs of known molecular epitope specificities. It appeared that 48 Abs recognized hCGβ-, 8 hCGα-, and 13 αβ-heterodimer-specific epitopes. Twenty-seven mAbs were of pan hCG specificity, two thereof with no (<0.1 %; epitope β_1_), 12 with low (<1.0 %; epitopes β_2/4_), and 13 with high (>>1 %; epitopes β_3/5_) hLH cross-reactivity. The majority of hCGβ epitopes recognized were located in two major antigenic domains, one on the peptide chain of the tips of β-sheet loops 1 and 3 (epitopes β_2–6_; 27 mAbs) and the second around the cystine knot (e.g., epitopes β_1_, β_7_, and β_10_; 9 mAbs). Four mAbs recognized epitopes on hCGβcf-only (e.g., epitopes β_11_ and β_13_) and six mAbs epitopes on the remote hCGβ-carboxyl-terminal peptide (epitopes β_8_ and β_9_ corresponding to amino acids 135–144 and 111–116, respectively). For routine diagnostic measurements, methods are used that either detect hCG-only, hCGβ-only, or hCG together with hCGβ or hCG together with hCGβ and hCGβcf. Sandwich assays that measure hCG plus hCGβ and eventually hCGβcf should recognize the protein backbone of the analytes preferably on an equimolar basis, should not cross-react with hLH and not be susceptible to blunting of signal by nonmeasured variants like hCGβcf. Such assays can be constructed using pairs of mAbs directed against the cystine knot-associated epitope β_1_ (Asp10, Asp60, and Gln89) in combination with epitopes β_2_ or β_4_ located at the top of β-sheet loops 1 + 3 of hCGβ involving aa hCGβ20-25 + 68-77. In summary, the results of the First and Second ISOBM TD-7 WSs on hCG provide the basis for harmonization of specificities and epitopes of mAbs to be used in multifunctional and selective diagnostic hCG methods for different clinical purposes.

**Electronic supplementary material:**

The online version of this article (doi:10.1007/s13277-013-0994-6) contains supplementary material, which is available to authorized users.

## Objective

Improving between method comparability for measurement of the heterogeneous glycoprotein hCG requires harmonization of epitopes of the Abs used and broad consensus about assay specificity. To address this, the Second International Society of Oncology and Biomarkers Tissue Differentiation 7 (ISOBM TD-7) Workshop (WS) by a three-step algorithm characterized and epitope typed 69 Abs directed against hCG and variants submitted by diagnostic companies and research groups. The results of this WS in combination with those of the First WS enable recommendations to be made regarding epitope combinations to be used for the design of immunoassays for hCG and its variants [1].

## Introduction

### Physiology, protein structure, and posttranslational protein backbone variants of hCG

The glycoprotein hormone hCG is essential for maintaining pregnancy. Physiologically, it is produced and secreted by the placental trophoblast and pathophysiologically by trophoblastic cancers and by germ cell tumors of the testis and ovary [2].

hCG is a protein heterodimer consisting of hCGα noncovalently linked to the hCGβ subunit. As all glycoprotein hormone (GPH) subunits, hCGα and hCGβ share structural homology with members of the cystine knot growth factor superfamily that includes nerve growth factor, platelet-derived growth factor and transforming growth factor beta [3]. The common structural cystine knot motif consists of two disulfide bridges that link adjacent antiparallel strands of the single peptide chain to form a ring that is axially permeated by a third disulfide bond. This central cystine knot determines the three-dimensional structure of hCGα and hCGβ. On one side of the knot, there are two neighboring hairpin-like peptide loops 1 and 3, which, in hCGβ, are stabilized by a disulfide bond between Cys 23 and Cys72. The single larger loop 2 is located on the opposite side of the knot [3].

The subunits are noncovalently linked in antiparallel, i.e., a head-to-toe fashion, such that loops 1 + 3 of one subunit are adjacent to loop 2 of the other subunit [3]. Loops 1 and 3 of either subunit and the hCGβ cystine knot, respectively are the most important antigenic regions [1].

The hCGβ genes have developed from an ancestral LHβ gene by gene duplications and mutations [4]. The hCGβ protein is 145 amino acids (aa) in length and encoded by 4 genes and 2 alleles (CGβ6/7, CGβ3/9, CGβ5, and CGβ8), while hLHβ is encoded by a single gene, CGβ4, on chromosome 19q13.3. Thus, hCGβ and hLHβ are highly similar in protein sequence (>85 %) and are immunologically closely related. Furthermore, LH and hCG activate the same receptor. The major structural difference between hCGβ and hLHβ is a carboxyl-terminal peptide extension of hCGβ (hCGβCTP) encompassing aa 113–145. hCGβCTP evolved through a read-through event due to a mutational loss of the stop codon at the genomic level and the incorporation of a hitherto untranslated gene sequence into the coding region [5]. Antibodies recognizing epitopes on hCGβCTP are used in a number of highly specific hCG assays [6]. A single gene on human chromosome 12q21.1-23 encodes the α-subunit, which is 92 aa in length and common to all four human GPHs [7].

**Fig. 1:**
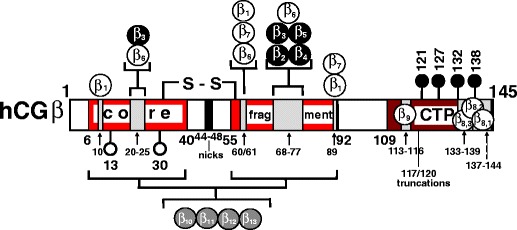
Schematic representation of human chorionic gonadotropin β (hCGβ) protein backbone variants and molecular epitope localizations on assembled and free hCGβ (amino acids, aa hCGβ1-145), hCGβ core fragment (hCGβcf, aa hCGβ6-40 + β55-92), and the carboxyl-terminal peptide (hCGβCTP, aa hCGβ109/113-145). Modified according to [1] with permission (INN). Antigenic determinants are diagrammatically represented on the linear aa sequence. Non-assembled hCGβ carries nine epitopes (β_1_–β_9_), seven are present also on the hCGαβ-heterodimer (β_1_–β_5_, β_8_, β_9_), and all, except those on the hCGβCTP (β_8_, β_9_), are located within the amino acid sequences (aa) of hCGβcf. Four additional specific epitopes are present on hCGβcf only (β_10_–β_13_) but not on intact hCGβ and hCG. All epitopes that are located on core hCGβ (aa 1–112) are conformationally dependent and determined by the tertiary protein structure. Important residues contributing to these epitopes at the primary sequence level were identified by selective mutational analyses: Pro24, Val25, Arg68, Gly71, and Gly75 contribute to epitope β_3_, aa Lys20, Glu21, Gln22, Gly75, and Asn77 to free subunit epitope β_6_ and Arg68 to structurally overlapping epitopes β_2_, β_3_, β_4_, and β_5_ [22, 42]. hCG-specific epitope β_1_ is built up by cystine-knot associated Arg10 and Arg60 and to a minor extent Gln89 as it does in epitope β_7_. Asp61 plays a role in free subunit epitopes β_6_ and β_7_ [43]. Major antigenic regions of hCGβCTP are rather linear in nature and determined by the primary structure: aa hCGβ133-144 comprising epitope β_8_, that is substructured into β_8,1_ to β_8,3_ and aa hCGβ113-116 corresponding to epitope β_9_ [21, 24, 29, 50, 71]. *Numbers* represent positions of amino acid residues in the peptide chain. The metabolic product hCGβcf consists of two peptide fragments that are linked via five disulfide bonds (depicted by S–S), and its N-linked carbohydrate antennae are truncated. *Open circles* N-linked glycans, *filled circles* O-linked glycans

hCG is heterogeneous with respect to protein backbone structure and carbohydrate content and is best considered as a complex family of hCG variants occurring in body fluids and tissues. The unambiguous nomenclature for the most important hCG forms of the protein backbone developed by the International Federation of Clinical Chemistry (IFCC) Working Group for Standardization of hCG Determinations is used here (Table 1 and Fig. 1) [1, 8, 9].

**Table 1:** Nomenclature of hCG and hCG-related variants (modified according to [1] with permission)

Symbol	Molecular definition
hCG	Intact αβ heterodimer, bioactive
hCGn	Nicked αβ heterodimer, nicks in the region of aa hCGβ44-48
hCGβ	Intact noncombined free hCGβ-subunit, aa hCGβ1-145
hCGβn	Nicked hCGβ, nicks in the region of aa hCGβ44-48
hCGβcf	Core fragment of hCGβ; aa hCGβ6-40 linked to hCGβ55-92
hCGα	Noncombined free α-subunit of hCG; aa hCGα1-92
Less well-defined hCG variants	
hCGβCTP	Carboxylterminal extension of hCGβ, aa hCGβ109/114-145
-CTPhCG	hCGβ truncated core hCG, missing most of the hCGβCTP (aa hCGβ121-145)
-CTPhCGβ	hCGβ truncated core hCGβ (aa hCGβ1-120), missing most of the hCGβCTP

Abbreviations and definitions for hCG and hCG-derived molecules as established by the IFCC Working Group for Standardization of hCG [1, 2].

*aa* amino acids

### Glycosylation isoforms

**Fig. 2:**
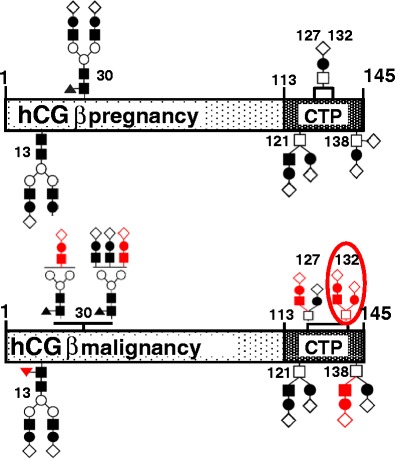
Glycosylation variants of hCGβ (according to [11]). In pregnancy-derived hCGβ, the N-linked carbohydrates are of the biantennary type. O-Glycosylation of hCGβ at Ser 121 always contains a biantennary core-2 and at Ser 138 a core-1 structure with one or two sialic acids. Malignancy-derived hCG and very early pregnancy hCG as compared to middle-to-late pregnancy hCGβ is characterized by increased content of triantennary complex-type N-linked carbohydrates attached to hCGβ Asn 30 and fucosylated carbohydrates attached to Asn 13. “Hyperglycosylated” hCGβ contains an increased proportion of triantennary N-linked carbohydrates (Asn 30); core-2 type O-glycans at Ser 127, Ser 132, and Ser 138; and fucosylated Asn 13-linked glycan. Some glycosylation sites were not glycosylated in some variants (Ser 138, Ser 121, and Asn 13). Immunoassays for hCG-h based on mAb B152 recognize the encircled glycan at Ser 132 and surrounding peptide structure. The major differences in carbohydrate antennae composition between early and mid-to-late pregnancy- and malignancy-derived hCG are depicted in *red*. *Filled square* GlcNAc, *filled diamond* Fuc, *empty square* GalNAc, *empty circle* Man, *filled circle* Gal, *empty diamond* NeuAc

hCG subunit folding, assembly, intracellular trafficking, secretion, receptor activation, and half-life in serum is dependent on glycosylation [10]. Both hCG subunits are glycosylated: hCGα contains two N-glycosylation sites at Asn52 and Asn78 that are either mono-, bi-, or triantennary or are sometimes missing. Most N-linked carbohydrate antennae at Asn13 and Asn30 of hCGβ are of the bi-antennary type, but malignancy-associated hCG increasingly carries triantennary carbohydrates at Asn30 and fucosylation at Asn13 (Fig. 2). Four putative O-glycosylation sites are located at Ser121 (core-2), Ser127 (core-1), Ser132 (core-1), and Ser138 (core-1) on the hCGβCTP. The pregnancy associated core-1 glycans on Ser127 and Ser132 are frequently replaced by core-2 glycans in hCG synthesized in early pregnancy and by tumors [11].

Due to variability in branching of carbohydrate antennae and terminal sialylation (8–15 sialic acids), numerous isoforms exist [11, 12]. The relative proportions of more extensively glycosylated and terminally sialylated glycosylation variants change with advancing pregnancy, tumor progression, and between different tumors [13–15]. Consequently, acidic variants (av) of hCG (avhCG) produced by testicular cancer [16], by other tumors, and in early pregnancy [13] have very low isoelectric points (p*I*s) and higher MWs [15, 17, 18]. The expression avhCG, describing hCG with complex extensively terminally sialylated carbohydrate antennae, was later replaced by the term “hyperglycosylated” hCG (hCG-h) [19]. Presently, hCG-h is defined as hCG isoforms carrying a biantennary core-2 O-glycan on Ser132 that is detected by immunoassays using a mAb-designated B152 (Fig. 2) [20].

### hCG epitopes

Elucidation of the three-dimensional structure of hCG [3] provided the basis for assignment of immunologically and biologically important domains to the molecular surface of hCG and hCG-related molecules. Several strategies were pursued to resolve epitope distribution and arrangement as well as identification of immunodominant regions. Epitope localization and sharing of epitopes among hCG, hCG-variants, subunits, and related hormones like LH and subunits were determined using molecular chimeras, hCG metabolites, homologous and heterologous glycoprotein hormones and subunits, chemically modified hormones, proteolytic hormone fragments and synthetic peptides, including peptide scanning, and most importantly by site-specific mutagenesis of hCGβ. It is important to mention that in three independent laboratories with different sets of mAbs and analytical techniques similar epitopes and antigenic domains were defined (for reviews, see [1, 21].

In previous studies, 26 epitopes on hCG and hCG-related molecules were defined (for reviews, see [1, 20, 22]). Sixteen epitopes are located on the intact holo hormone hCG (epitopes β_1_–β_5_, β_8_, and α_9_; α_1_–α_5_; and c_1_–c_4_). Seven of these are present on both free and assembled hCGβ (β_1_–β_5_, β_8_, and α_9_; Fig. 1 and Table 2).

**Table 2:**
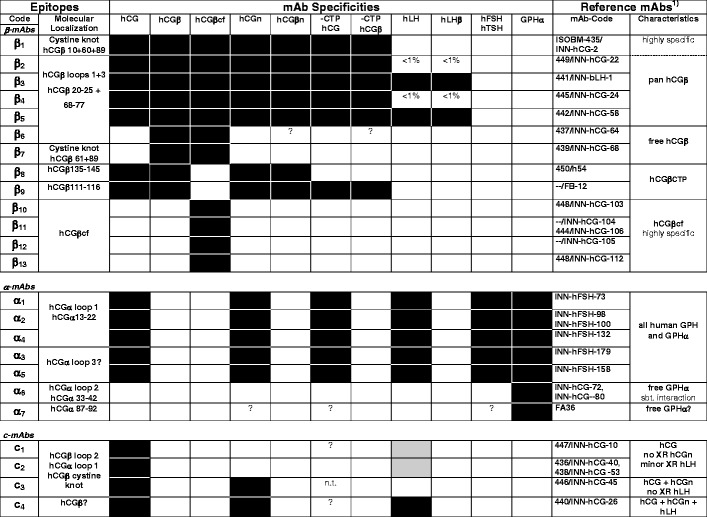
hCG reference-mAbs: molecular localization of epitopes and specificity patterns (modified according to [1], with permission)

*GPHα* glycoprotein hormone alpha subunit, *n.t.* not tested

^a^“INN-” reference mAbs can be obtained from the author (P.B.); filled squares, strong reactivity; open squares, no reactivity; gray squares, minor reactivity

Antibodies against epitopes β_1_–β_5_ recognize a wide range of hCG and hCGβ variants (pan hCG-mAbs), including hCG + hCGn + hCGβ + hCGβn + hCGβcf. In a first step, epitopes recognized by these mAbs can be discerned by their cross-reactivity with hLH or hLHβ: Abs directed against (1) epitope β_1_ have no (<0.1 %), (2) epitopes β_2_ and β_4_ <1%, and (3) epitopes β_3_ and β_5_ >>1 % hLH cross-reactivity. Epitopes β_1_–β_5_ are distributed among two antigenic domains: (1) the cystine knot (epitope β_1_) and (2) hCGβ loops 1 + 3 comprising the neighbouring epitopes β_2_–β_6_. Epitopes β_8_ andβ_9_ are located on the hCGβCTP and by definition are specific for hCG and hCGβ [1, 23, 24].

A number of epitopes are of restricted variant specificity. Abs against such epitopes are useful for variant-selective immunoassays designed to measure hCG, hCG + hCGn, hCGβ, hCGβ + hCGβcf, hCGβcf, or hCGα, respectively, in the presence of excess of other hCG protein backbone variants and GPHs.

Epitopes β_6_ (hCGβ loops 1 + 3 related) and β_7_ (cystine knot related) are shared by free hCGβ and hCGβcf but not by holo-hCG. Epitope β_14_, which is related to the core region of hCGβ (amino acids 1–112) and maybe cystine knot-related, was defined in the First ISOBM WS. It is specific for free hCGβ and not shared by hCGβcf [1]. Four hCGβcf-specific epitopes β_10_–β_13_ (of which β_10_ and β_12_ probably are hCGβ cystine knot-related, PB unpublished data) are not present on hCGβ, hCGβn, hCG, hCGn, or on hLH/hLHβ/hLHβcf. Two epitopes, α_6_ (hCGα loop 2 related) and α_7_ (hCGα carboxyl-terminal related), are specific for nonassembled hCGα.

Some additional Abs recognize epitopes defined only broadly at the molecular level, e.g., additional c- or β-mAbs. Within antigenic domains there seem to be epitopes that remain to be defined more precisely [1].

### Algorithm to define hCG epitopes of ISOBM mAbs (INN)

For the Second ISOBM TD-7 WS on hCG, a previously reported epitope mapping algorithm [22], which was also used in similar form in the First ISOBM TD-7 hCG WS [1] has been further refined to reliably characterize the specificities and epitopes of the 69 ISOBM-Abs. This three-step algorithm involves:

1. Determination of intraspecies Ab specificities with hCG, hCG-related variants [six First International Reference Reagents (IRR) preparations], hLH and synthetic hCGβCTP peptides to enable grouping of the mAbs according to their main specificities (α-, β-, and c-mAbs), and tentative assignment of epitopes by comparing specificity profiles to those of reference mAbs with known epitope recognition
2. Confirmation of epitope recognition and spatial arrangement of epitopes using sandwich assays with mutual antigen recognition or inhibition by pairs of mAbs to provide information about epitope disparity or identity/vicinity
3. Cross-referencing of the ISOBM-Abs’ reaction profiles in specificity and sandwich assays to those of reference mAbs recognizing previously defined epitopes.

This approach frequently enabled definitive assignment of epitopes at the molecular level. In rare cases where no exact molecular localization could be determined due to the lack of appropriate reference mAbs, additional circumstantial evidence, e.g., mutual steric inhibition in simultaneous antigen recognition with mAbs of known epitope localization or recognition of breakdown products like hCGβcf or inter-species cross-reactivity, was used to elucidate the epitope’s antigenic domain [1].

## Materials and methods

### ISOBM-Abs: codes and descriptions

Sixty-nine ISOBM-TD-7 Abs were submitted by eight participants to Dr. Kjell Nustad at the Central Laboratory, Norwegian Radium Hospital (NRH), Oslo, Norway (see Table 7 in Appendix). The Abs were assigned code numbers ISOBM-382 to 450. The panel contained (1) 42 Abs to be tested for molecular epitope recognition, (2) 10 mAbs that were previously specificity- and epitope-typed in the First ISOBM TD-7 WS on hCG as blinded internal controls: ISOBM-403 is identical to reference mAb -435 and corresponds to ISOBM-265 and ISOBM-274 in the First WS, ISOBM-411 is identical to -275 (First WS), ISOBM-415 to -281; ISOBM-416 to -273; ISOBM-417 to -276; ISOBM-418 to -280; ISOBM-419 to -271; ISOBM-420 to -264 and -277; ISOBM-422 to -272, and ISOBM-424 to -279 [1] (Appendix); and (3) 17 reference mAbs (ISOBM-434–450) of known specificity and epitope recognition provided by Dr. Peter Berger from the Institute for Biomedical Aging Research, Innsbruck (INN), Austria. The hybridomas producing mouse reference mAbs (INN-mAbs) against hCG, hCGβ, hCGβcf, and hCGβCTP were established as previously described [23, 25–31] and specificity, affinity, and epitope analyses by a panel of immunochemical techniques (for reviews, see [1, 22]).

The 17 reference mAbs were directed against 15 epitopes on hCG and hCG-related molecules (Table 2) (for reviews, see [1, 22]). Ten epitopes were located on intact hCG (epitopes β_1_–β_5_ and β_8_; c_1_–c_4_), and six of these shared by hCGβ (β_1_–β_5_, and β_8_). Two Abs recognized epitopes on hCGβ plus hCGβn plus hCGβcf (β_6_ and β_7_). Three reference mAbs against epitopes β_10_, β_11_, and β_13_ recognized exclusively hCGβcf. MAb FB12 recognizing hCGβCTP epitope β_9_ [32], ISOBM-278 (epitope (β_8_ type 1, β_8,1_) ISOBM-277 (epitope β_8_ type 2, β_8,2_) and ISOBM-267 (epitope β_14_) [1] were additionally used as control reagents. No reference or control mAbs for epitopes α_1_–α_7_, β_12_, and β_8/3_) were applied in the specificity and epitope typing experiments.

The Abs were checked for purity by sodium dodecyl sulfate polyacrylamide gel electrophoresis (SDS-PAGE), the protein content determined by measuring the absorbance at 280 nm (1 mg/mL = 1.43), aliquoted and 1 mg of each sent to the laboratories of the workshop participants performing the experimental work: Dr. Phil Hemken, Diagnostic Research and Development, Abbott Diagnostics (ABB); Dr. Elisabeth Paus, Radiumhospitalet, Oslo University Hospital, (NRH); Dr. Ulf-Håkan Stenman (UHS), Helsinki University Central Hospital; and Dr. Wilson Stewart, Ninewells Hospital and Medical School, Dundee (NHD).

### First international reference reagents for hCG and hCG variants

The new international standards for hCG, nicked hCG (hCGn), hCGα, hCGβ, hCGβn, and hCGβcf were purified and characterized by the IFCC Working Group for Standardization of hCG Determinations [9] and adopted by the WHO as the First IRR for hCG and related variants [33]. The material is intended for use in the calibration of immunoassays in substance concentrations, i.e., moles per liter [6]. One milligram each of the six First IRRs for hCG and related molecules were kindly supplied by the NIBSC (Dr. Catharine Sturgeon, CS) to each of the participants and used to characterize the 69 ISOBM-Abs (Table 3).

**Table 3:** The WHO 1^st^ IRRs for hCG and related variants and 5^th^ IS for hCG

Symbol	WHO code	Content/ampoule
hCG	5^th^ IS 07/364^a^	0.39 nmol or 179 IU
hCG	1^st^ IRR 99/688	1.88 nmol
hCGn	1^st^ IRR 99/642	0.78 nmol
hCGβ	1^st^ IRR 99/650	0.84 nmol
hCGβn	1^st^ IRR 99/692	0.88 nmol
hCGβcf	1^st^ IRR 99/708	0.33 nmol
hCGα	1^st^ IRR 99/720	1.02 nmol

^a^ The 1^st^ IRR 99/688 for hCG has been adopted as the new 5^th^ IS 07/364 for hCG

For iodination, FRET and BIAcore® specificity and affinity determinations the carrier-free frozen concentrates (FC) of the six First IRRs were used: hCG (FC 99/688), hCGn (FC 99/642), hCGβ (FC 99/650), hCGβn (FC 99/692), hCGβcf (FC 99/708), and hCGα (FC 99/720).

### Other hormones and peptides

Human LH (hLH-I-1) AFP4345B for iodination was obtained from National Hormone & Peptide Program, USA. The peptide hCGβCTP135-145, PGPSDTPILPQ, was ordered from AltaBioscience, UK. The peptide hCGβCTP109-145, TCDDPRFQDSSSSKAPPPSLPSPSRLPGPSDTPILPQ, was provided by Dr. Jean-Michel Bidart.

### Biochemical characterization of the mAbs (ABB, NRH)

The mAbs were biochemically characterized by gel permeation chromatography–high performance chromatography (GPC-HPLC; ABB; Online Resource 1), SDS-PAGE under reducing (ABB; Online Resource 2) and non-reducing conditions (NRH; Online Resource 3), Ab isotyping (ABB, NRH; Online Resource 4), isoelectric focusing (IEF; ABB; Online Resource 5), and finally mass spectrometry (MS; ABB; Online Resource 6), which was utilized for further characterization of Ab samples where double heavy or double light chain bands were observed using SDS-PAGE testing.

### Determination of Ab specificity, affinity, and epitope localization (ABB, NRH)

The main specificity profiles of mAbs were determined (1) by direct binding RIA (DB-RIA) with ^125^I-labeled hormones and hormone fragments with excess Ab (Online Resources 7, 8) and (2) with competitive ligand analysis (CLA), a RIA format, wherein the binding between ^125^I-hCG and serial diluted Abs is competed with fixed concentrations of the six First IRRs of hCG and hCG-related molecules and hLH (75/552), respectively (Online Resource 9). Cross-reactivity of the ISOBM-Abs with hLH was determined by titration RIA (NRH) by comparing titers of ^125^I-labeled hCG versus ^125^I-labeled LH (Online Resource 10). Epitope recognition on the hCGβCTP by ISOBM-Abs was evaluated by competitive RIA with synthetic peptides (NRH; Online Resource 11). Ab affinities were determined by Forster Resonance Energy Transfer (FRET) (ABB; Online Resource 12) and by BIAcore® (NHD; Online Resource 13). For elucidation of the spatial arrangement of epitopes, Ab compatibility in antigen recognition was evaluated by sandwich RIA (NRH; Online Resource 14).

## Results

### Biochemical characterization of the mAbs (ABB; NRH)

#### Gel permeation chromatography–high performance chromatography (ABB)

**Fig. 3:**
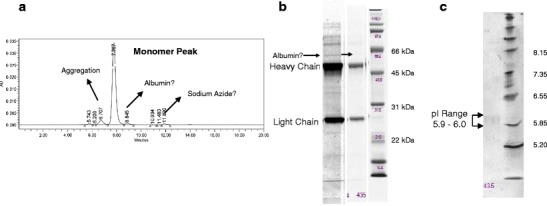
Biochemical characterization of ISOBM-435 by GPC-HPLC (**a**), reduced SDS-PAGE (**b**), and IEF (**c**). **a** Percent purity by GPC-HPLC analysis is 96 % with 7 % aggregation and a small amount (2 %) of low molecular weight (*LMW*) contaminants. The LMW material could be sodium azide and residual albumin, but this was not confirmed. **b** Reduced SDS-PAGE analysis shows a combined heavy and light chain purity of 96 %. The first strip has been enhanced to increase the image contrast to highlight a faint band (<1 %) that has a similar molecular weight as the albumin standard (66 kDa). The second strip utilized the auto-scale feature available with Quantity One software (Bio-Rad). A 1 % band is noted at approximately 76 kDa, and a 3 % band is also seen at approximately 47 kDa. **c** IEF reveals a tight p*I* range of 5.9–6.0.

The homogeneity of the mAbs determined by GPC ranged from 57 to 99 % with varying degrees of aggregation and low MW contaminants (Fig. 3a; Online Resource 15). All samples exhibited low MW peaks possibly caused by buffer components (azide, citrate, DTT, etc.).

#### Sodium dodecyl sulfate polyacrylamide gel electrophoresis (NRH, ABB)

SDS-PAGE analysis was performed under nonreducing (Online Resource 16a; NRH) and reducing conditions (Online Resource 16b; ABB). The purity of the mAbs determined as the proportion of heavy and light chains relative to all protein bands ranged from 84 to 100 % (Online Resource 16b). An example for slight albumin impurity is shown in Fig. 3b. The appearance of double chains could be due to glycosylation differences [34, 35], amino acid residue issues [34, 35], or the presence of more than one Ab in the sample (Online Resource 16b).

#### Isoelectric focusing (ABB)

The isoelectric point (p*I*) of the Abs ranged from 5.0 to 7.7 (Fig. 3c; Online Resource 17). Those for ISOBM-397, ISOBM-418, and ISOBM-431 could not be determined. The smearing or absence of bands was probably due to low solubility of these Abs at the low ion strength in IEF.

#### Isotyping (ABB)

Isotyping was performed on samples displaying double heavy or light chains in SDS-PAGE analysis (Table 4).

**Table 4:** ISOBM-Abs, biochemical characterization (ABB)

ISOBM	GPC-HPLC TOSOH G3000 SWxl column^a^			SDS-PAGE^b^	Isotype^b^	Phastsystem IEF^b^
Code	% Purity	% Aggregation	% Low MW^c^	% Purity	Heavy/light chain determination	p*I* range
382	95	5	<1	99^d^	IgG1, kappa	5.8 – 6.2
383	98	ND^e^	1	100	IgG1, kappa	6.2–6.8
384	97	2	<1	95	NT^f^	5.1–5.3
385	97	2	1	100^g^	IgG1, kappa	6.9–7.7
386	89	10	<1	96	NT	6.7–7.2
387	98	1	<1	100	NT	6.0–6.4
388	97	2	<1	99^d^	IgG1, kappa	6.3–7.2
389	98	1	<1	99	NT	5.9–6.2
390	98	1	<1	98	NT	6.8–7.6
391	90	1	9^h^	100	NT	6.4–7.4
392	92	7	<1	94	NT	5.5–5.9
393	83	5	12^i^	97	NT	5.6–6.0
394	88	<1	11^h^	100	NT	6.7–7.3
395	97	3	<1	100	NT	6.9–7.5
396	98	2	<1	99	NT	6.2–6.5
397	96	3	<1	99	NT	Indistinct^j^
398	97	2	<1	99	NT	6.2–6.6
399	88	11	<1^k^	99	NT	6.4–6.7
400	96	2	2	96^d^	IgG1, kappa	6.1–6.6
401	98	2	<1	98	NT	6.6–7.3
402	97	1	1	100	NT	6.5–7.2
403	97	2	<1	100	NT	6.0–6.3
404	98	2	<1^k^	99	NT	6.2–6.8
405	94	5	<1^k^	99	IgG1, 2a, 2b, kappa^l^	5.5–5.8, 6.1–6.6^m^
406	71	ND	29^h^	100^d^	IgG1, kappa	5.0–5.3
407	91	8	1^k^	99	NT	6.2–6.6
408	89	1	10^h^	100	NT	6.1–6.4
409	64	ND	36^h^	100	NT	6.1–6.4
410	99	1	<1	99	NT	6.8–7.3
411	94	ND	6^n^	96, 2^o^	NT	6.0–6.3
412	95	1	3^h,p^	94	NT	5.4–5.7
413	90	7	2^h^	99	NT	6.5–7.2
414	86	5	10^h^	96	NT	6.1–6.4
415	98	<1	1^h^	100	NT	6.0–6.3
416	74	<1	26^h^	99	NT	5.8–6.2
417	97	1	2^n^	99, 1^o^	NT	6.3–6.6
418	99	<1	<1	98	NT	Indistinct^j^
419	86	3	10^h^	96	NT	5.6–6.0
420	99	<1	<1	100	NT	6.6–7.5
421	97	2	<1	100	NT	6.1–6.6
422	83	1	16^h^	100	NT	5.6–6.0
423	96	2	1^h^	99	NT	6.1–6.5
424	98	<1	<1	99	NT	6.2–6.8
425	98	<1	1	100	NT	6.0–6.7
426	98	1	1^h^	100^g^	IgG1, kappa	6.1–6.4
427	97	2	<1^h^	99	NT	6.0–6.5
428^q^	81	11	8^n^	98, 2^o^	NT	6.0–6.1
429^q^	92	4	3	100	NT	5.9–6.1
430^q^	97	1	2	100	NT	6.0–6.2
431^q,r^	89	ND	ND	100	NT	ND
432	95	4	1	99	NT	6.5–7.0
433	97	2	<1	99	NT	5.8–6.2
434	88	4	7^n^	91, 2^o^	NT	6.1–6.3
435	91	7	2^n^	96, 1^o^	NT	5.9–6.0
436	86	13	1^n^	86^d,g^, 1^o^	IgG1, lambda and kappa^s^	5.7–6.1
437	88	10	<1	96	NT	6.0–6.3
438	87	11	1^n^	96, 1^o^	NT	5.5–5.7
439	93	5	1^n^	95, 1^o^	NT	6.3–6.9
440	93	2	5^n^	91^d^, 2^o^	IgG1, kappa	5.9–6.0
441	83	12	5^n^	94, 2^o^	NT	6.2–6.5
442^q^	57	35	8^i,n^	85^d^, 1^o^	IgG1, kappa	5.4–5.7
443	90	9	1	99	NT	6.3–6.6
444	93	4	3^n^	95, 1^o^	NT	6.0–6.3
445	72	25	3^n^	84, 3^o^	NT	5.3–5.5
446	95	3	2^n^	97	NT	6.1–6.4
447	97	2	<1	98	NT	6.3–6.6
448	92	3	5^n^	87^g^, 3^o^	IgG2a, kappa	5.8–6.9
449	87	12	<1	95	NT	6.1–6.5
450	89	3	7^i,n^	90^d^	IgG1, kappa	6.0–6.7

^a^GPC-HPLC samples were run in triplicate. Mean values are ±1.9 %, which is established from the largest standard deviation (SD) observed. Triplicate injections of sample 442 had the largest SD of 1.91.

^b^Single lanes or strips were run for these tests, to preserve sample for additional testing.

^c^All the samples have peaks with the same retention time as sodium azide. The presence of sodium azide could not be confirmed due to lack of sample volume to perform additional testing.

^d^Double light chains. Possible causes for double chains include but are not limited to; glycosylation differences, amino acid residue differences or more than one antibody present in the sample

^e^Not Detected

^f^Not Tested, only samples exhibiting double heavy or light chains by SDS-PAGE or multiple clusters of bands by IEF underwent isotype analysis.

^g^Double heavy chains.

^h^These samples have peaks that could represent high levels of residual citrate in these samples. Residual testing would need to be performed to confirm this. Testing was not performed due to lack of sample volume. High levels of citrate can react with iron that may be present in HPLC equipment forming iron-citrate complexes. This phenomenon has been observed in other samples containing high citrate levels at retention times of approximately 11.3 minutes using a G3000SWxl Column and our Waters HPLC system.

^i^A tailing shoulder is present behind the main antibody peak.

^j^The sample only produced a smear, possibly due to a high salt concentration.

^k^Retention times of 9.2 to 9.6 minutes correspond to a molecular weight of 20–30 kDa and could represent free light chain material. The molecular weight determination was obtained by plotting the logarithm of the GFS molecular weights versus their retention times.

^l^Two types of heavy chain isotypes indicate this sample is probably not derived from a single clone.

^m^Two pI ranges indicate this sample may not be derived from a single clone.

^n^These samples have peaks that have a similar retention time as albumin. The presence of albumin could not be confirmed due to lack of sample volume to perform additional testing.

^o^A band was observed near the albumin standard.

^p^These samples have peaks that have a similar retention time as DTT. The presence of DTT could not be confirmed due to lack of sample volume to perform additional testing.

^q^Sheep Antibody

^r^The label concentration may be incorrect. The observed signals were not consistent with the label concentration.

^s^Two types of light chain isotypes indicate this sample is probably not derived from a single clone.

GFS Standards: thyroglobulin MW 670,000, RT 6.0–6.1; gamma-globulin MW 158,000, RT 7.9–8.0; ovalbumin MW 44,000, RT 9.2–9.3, myoglobin MW 17,000, RT 10.3–10.4, vitamin B12 MW 1,350, RT 11.9–12.0

#### Mass spectrometry analysis (ABB)

Apart from the following exceptions most ISOBM-Abs gave expected results. ISOBM-382 had double light chains, and more than one group of heavy chains present following deglycosylation, suggesting presence of two mAbs. ISOBM-385 had two groups of heavy chains due to glycosylation and ISOBM-388 two nonglycosylated light chains due to amino acid residue differences. ISOBM-400 and ISOBM-406 had more than one group of light chains due to differences in glycosylation and ISOBM-426 complex heavy chains and ISOBM-450 complex light chains (Online Resource 18).

#### Ab affinities, specificities, and epitope localizations

##### Ab specificities (NRH)

**Fig. 4:**
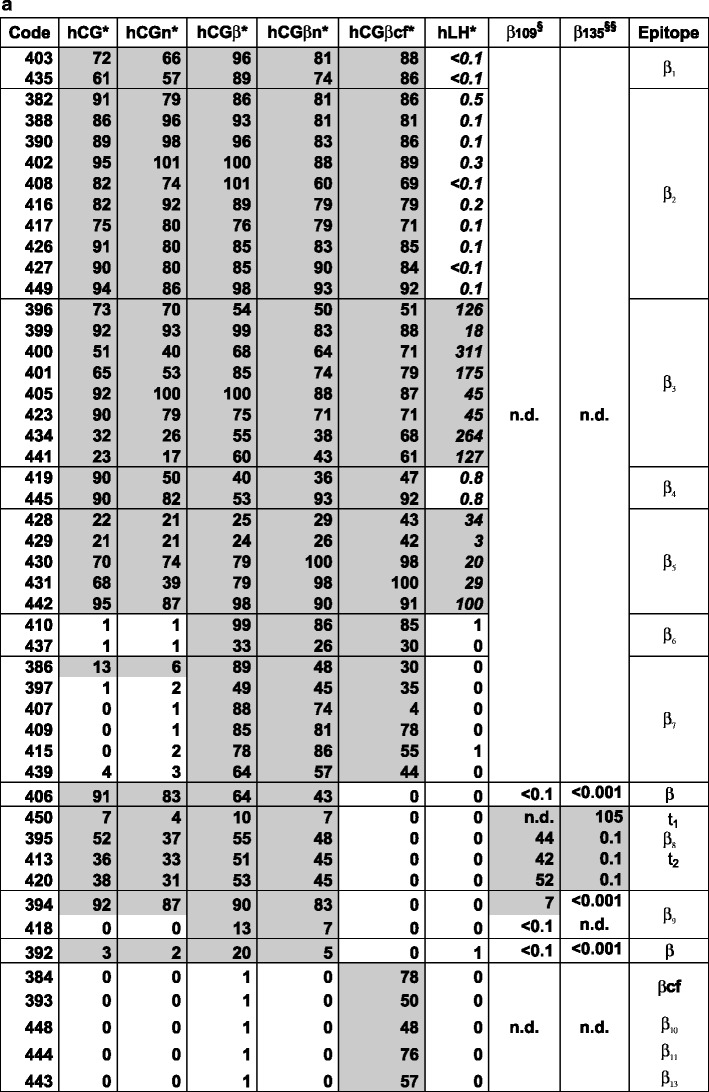

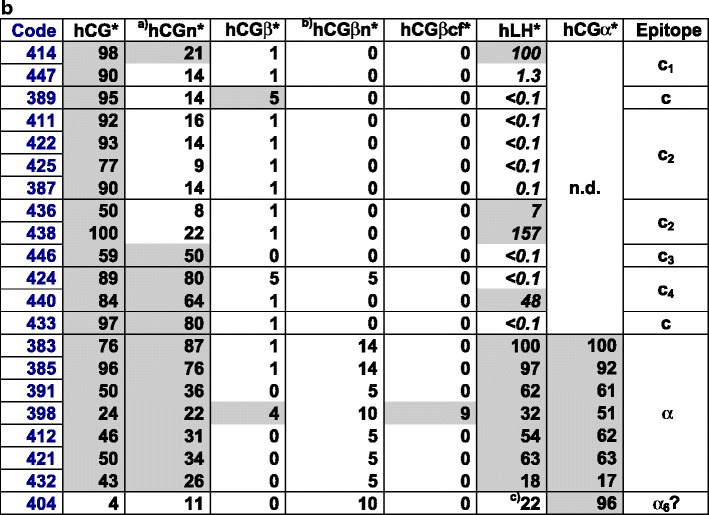
Specificity profiles of the ISOMB-Abs of the Second TD-7 WS recognizing hCG and hCGβ variants (**a**), hCG-only and hCGα, respectively (**b**) were determined by binding of iodinated tracers to excess of Ab (DB-RIA) (NRH). ISOBM-mAbs were classified according to their main specificities and their epitopes recognized on the basis of cross-reactivity patterns with hCG, hCG-variants, and hLH: (1) β-mAbs corresponding to epitopes β_1_–β_13_, (2) c-mAbs recognizing epitopes c_1_–c_4_ on holo-hCG only, and (3) α-mAbs. **a** MAbs directed against epitopes β_1_–β_5_ are pan-hCG reagents recognizing hCG and hCGβvariants but differ in their cross-reactivity with hLH: β_1_ mAbs are highly specific for hCG and show no hLH cross-reactivity (<0.1 %), β_2_ and β_4_ show very low hLH reactivity (<1 %), whereas β_3_ and β_5_ strongly cross-react (>>1 %). Epitopes β_6_ and β_7_ are specific for uncombined hCGβ, hCGβn, and hCGβcf. MAbs against epitope β_8_ at the very carboxyl-terminal end of hCGβCTP do not cross-react with hCGβcf and hLH but recognize all other hCG variants except for those lacking the CTP. These mAbs constantly show a low bindable fraction of the tracers as only approximately 50 % of the tracers can be bound specifically. This is in contrast to the β_1_–β_5_ mAbs. ISOBM-418 seems to be directed against epitope β_9_ as already typed previously in the First WS (ISOBM-280, [1]. Epitopes β_10_–β_13_ are specific for hCGβcf as no other hCG variants or hLH are recognized by the respective mAbs. **b** c-mAbs directed against epitopes determined by the quaternary structure of hCG either do not (c_1_ and c_2_) or do recognize hCGn (c_3_ and c_4_) [56]. The apparent hCGn cross-reactivity of c_1_ and c_2_ mAbs is due to a cross-contamination of this preparation with non-nicked hCG (approximately 20 %) [1]. The presence of non-nicked hCG and recognition by the ISOBM-mAbs of the two-nicked forms in hCGn were investigated in detail by LC-MS/MS (see accompanying publication by H. Lund). Epitope c_3_ (ISOBM-446 = INN-hCG-45, reference mAb) is highly specific for hCG + hCGn. ISOBM-mAb 433 that has the same specificity pattern might be directed against a fifth sterically independent c-epitope as shown by sandwich assay. The exact molecular localization of epitope c_4_ on hCG is not known, but it is remote from the other c-epitopes. In the First ISOBM TD-7 WS, ISOBM-424 has been characterized (ISOBM-279) and classified as c_4_ specific [1]. The α-mAbs have not been investigated in detail as to their epitope recognition. As they readily recognized iodinated tracers (in contrast to α_3_- and α_5_-mAbs), they should be directed against the epitope cluster α_1_/α_2_/α_4_ with the exception of ISOBM-404 that is free hCGα-specific and therefore presumably recognizing the subunit assembly region of hCGα (aa hCGα33-42). Minor apparent cross-reactivity with hCGn is owed to a cross-contamination of hCGα in that preparation. hLH cross-reactivity of ISOBM-404 might be due to dissociation of highly purified hLH that is observed during testing (PB, personal observation). DB-RIA with ^125^I-tracers: results are expressed as maximum specific binding in percent of the “bindable fraction” of added tracer (NRH) [26]. *Italics* RIA titration experiments (NRH): Results are expressed as percent hLH cross-reactivities compared to hCG; *asterisk* ^125^I-tracers; *gray background* significant cross-reactions. *Superscripted a* Apparent cross-reactivities with hCGn of ISOBM-447–438 are caused by an approximate 20 % cross-contamination of intact hCG (see accompanying publication by H. Lund) and *superscripted b* of ISOBM-383–404 due to a suchlike with hCGα that is contained in hCGβn; *superscripted c* ISOBM-404: apparent cross-reactivity is probably caused by slight dissociation of α-subunit in hLH. *Section symbol* Competitive RIA with hCGβ109-145 vs. hCGβ*, percent cross-reactivity. *Double section symbol* Competitive RIA with hCGβ135-145 vs. hCGβ*, percent cross-reactivity

Based on the results of DB-RIAs with ^125^I-labeled hCG, hCG-variants, and hLH tracers, the 69 ISOBM-Abs were categorized according to their main specificities (α-, β-, and c-mAbs) (Fig. 4): Antibodies either recognized (a) assembled and/or free hCGα (hCGα-mAbs, *n* = 8; α epitopes were not determined) or (b) assembled and/or free hCGβ or hCGβ metabolites such as hCGβcf (hCGβ-mAbs, *n* = 48; epitopes β_1_–β_13_), or (c) exclusively the intact ± nicked hCGαβ heterodimer, but not the free subunits or metabolic variants thereof (c-mAbs, *n* = 13; epitopes c_1_–c_4_).

Comparing specificity profiles of ISOBM-Abs to those of reference mAbs permitted preliminary epitope assignment. To discern mAbs against epitopes β_1_–β_5_, hLH cross-reactivity was determined by titration RIAs with ^125^I-hLH (Fig. 4). Recognition of hCGβCTP was investigated by competitive RIA using synthetic peptides derived from hCGβCTP (Fig. 4a; Online Resource 19). No mAbs against epitope β_14_ (hCGβ specific) were identified in this ISOBM panel (Fig. 4a).

##### Ab affinities and specificities as determined by FRET (ABB)

**Fig. 5:**
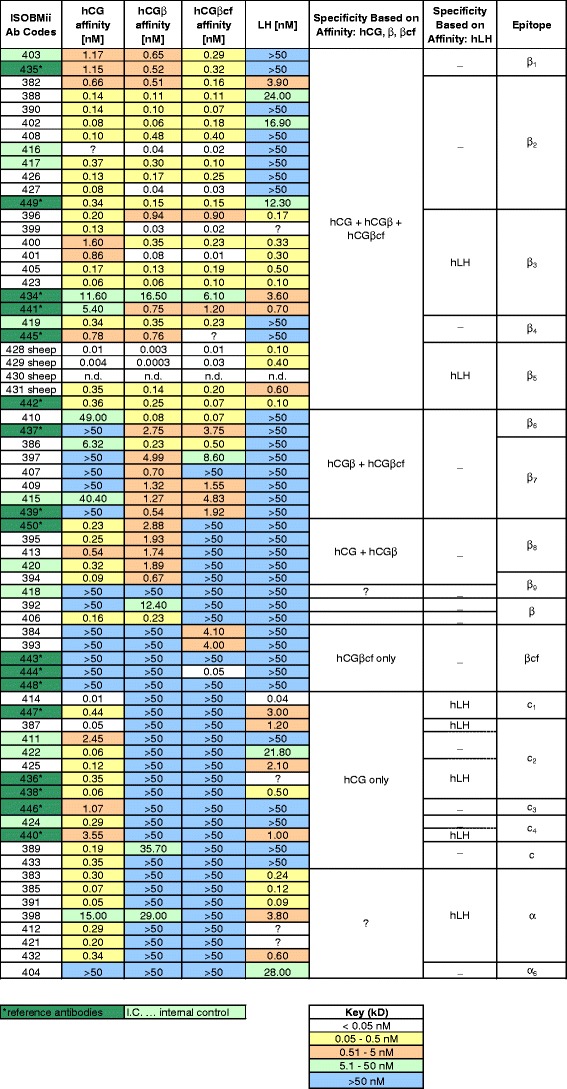
Affinity and specificity of the ISOBM-Abs as determined by FRET (ABB). Preliminary assignment of epitopes was done by comparing the specificity profiles of the ISOBM-Abs to those of reference mAbs. Specificities based on affinity of mAbs against hCGα could not be determined with hCG, hCGβ, and hCGβcf

The ISOBM-Abs were grouped according to their specificity profiles based on affinity for hCG, hCGβ, hCGβcf, and hLH (Fig. 5) determined by FRET. Affinities for the major hCG variants and hLH, reported as dissociation constants (*K*_d_), ranged from subpicomolar values (0.3 pmol/L for hCGβ of ISOBM-429) to ≥50 nmol/L. The latter value indicated that binding was very weak or not detectable.

Only nine Abs (ISOBM-387, ISOBM-399, ISOBM-401, ISOBM-414, ISOBM-416, ISOBM-427, ISOBM-428, ISOBM-429, and ISOBM-444) expressed high affinities (*K*_d_, ≤50 pmol/L) for any of the four antigens tested (hCG, hCGβ, hCGβcf, and hLH). It is striking that six of these mAbs recognize the major antigenic domain on the tips of hCGβ loops 1 + 3. An exception was the hCGβcf-specific mAb ISOBM-444 (reference mAb INN-hCG-106), the epitope of which (β_11_) does not overlap with epitopes β_2_–β_5_ on hCGβ loops 1 + 3 nor with the cystine knot-associated epitopes β_1_ and β_7_. Thus, this epitope is remote from either cluster. This is an interesting mAb for highly sensitive and specific measurement of hCGβcf in particular in combination with β_2_-mAbs [9, 36].

Another interesting observation is that all three sheep mAbs, ISOBM-428, ISOBM-429 and ISOBM-430, were in the high affinity group. ISOBM-430 was not tested by the FRET technology but by titration RIA. All four sheep Abs (three mAbs and polyclonal ISOBM-431) were directed against hCGβ loops 1 + 3 epitope β_5_ that is shared by hLH and, therefore, in principle, do not seem suitable for hCG measurement. Nevertheless, ISOBM-429 seems to have tolerably low cross-reactivity with hLH (Figs. 4a and 5 and Online Resource 20), but its suitability for use in hCG + hCGβ variant measurement might still be hampered by preferential recognition of hCGβ.

Most Abs, including all directed against the cystine knot-associated epitopes β_1_ and β_7_, the hCGβCTP epitope β_8_ types 1 and 2, showed moderate affinities (50 pmol/L–5 nmol/L) against their primary target hCG variant. Low affinities could be observed for three reasons: (1) the primary antigenic target hCG variant of the mAb in question was not among the antigens tested, as is the case of mAbs against uncombined hCGα (ISOBM-404), or (2) genuine low affinity to the primary target antigens, e.g., ISOBM-418/280 (hCGβCTP epitope β_9_, aa hCGβ113-116) and ISOBM-443 and 448 against hCGβcf, and (3) FRET labeling affected binding of Abs (ISOBM-445 to hCGβcf; ISOBM-399, ISOBM-436, ISOBM-412, and ISOBM-421 to hLH) (Fig. 5). This is also the case with ^125^I-labeling of epitopes α_3_ and α_5_ [37].

##### Ab affinities and specificities as determined by BIAcore® (NHD)

The specificity patterns of the ISOBM-Abs were determined based on their affinity for hCG, hCGβ, and hCGβcf in BIAcore®. The affinities (dissociation constants; *K*_d_) ranged from picomolar values (<10 pM for hCG of ISOBM-399) to >10 nM. An affinity of <100 nM was observed for 43 of the Abs for either a single or a combination of the three antigens tested (hCG, hCGβ, and hCGβcf). Five of these Abs, ISOBM-427 (epitope β_2_), ISOBM-399, ISOBM-423, ISOBM-441 (all three epitope β_3_), and ISOBM-428 (epitope β_5_) had affinities of <10pM for the antigen. It is striking that all of these recognize the tops of hCGβ loops 1 + 3; thus, all epitopes were located within the same antigenic domain. Assignment of epitopes to the ISOBM-Abs was achieved by comparing their specificity profiles to those of reference mAbs (Online Resource 20).

The affinity of many of the ISOBM-Abs appeared to be higher than determined by FRET analysis. This could perhaps be a consequence of having the antigen in a bound form on the BIAcore® chip rather than in a fluid state. The affinity for this immobilized form of antigen may result in an overestimate of affinity.

##### Ab specificities as determined by CLA (NHD)

In the CLA approach, ISOBM-Abs were titrated against ^125^I-hCG and in parallel competed with a fixed amount (0.5 pmol/mL) of hCG, hCG-variants, and hLH, respectively. A shift of the Ab dilution curves to a lower titre indicated cross-reactivity of this competitor with the Ab. Based on the CLA results, 34 ISOBM-Abs, either recognized (a) assembled and/or free hCGβ metabolites such as hCGβcf (hCGβ-mAbs, *n* = 24; epitopes β_1_–β_9_) or (b) exclusively hCG ± hCGβ, but not the free subunits (c-mAbs, *n* = 10; epitopes c_1_–c_4_). Epitope assignment was achieved by comparing profiles of the reference mAbs with ISOBM-Abs (Online Resource 20).

No CLA analysis was possible for 35 ISOBM-Abs, which had very low or no signal, which suggested that the Ab did not recognize ^125^I-hCG or could not be competed with the amounts utilized.

##### Epitope classification by sandwich assays (NRH)

**Fig. 6:**
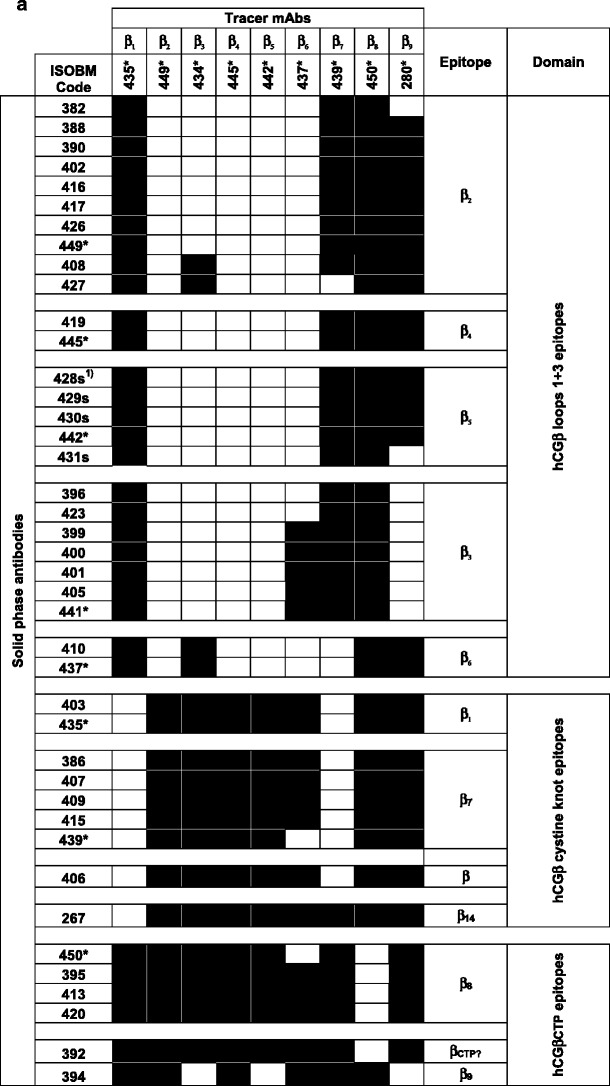

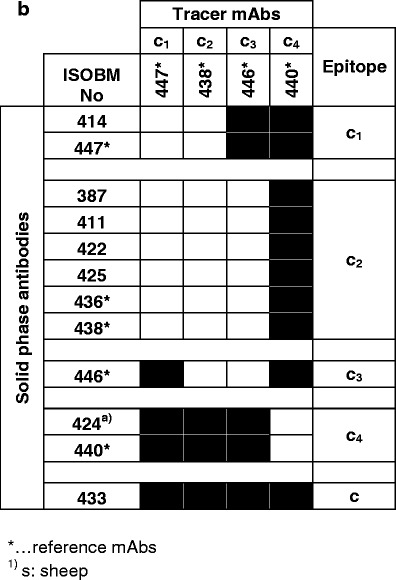
Classification and spatial relationship of ISOBM-mAb epitopes. Two-site IRMA-like sandwich assay experiments with a chessboard-like matrix of antibody pairs tested for their ability to simultaneously bind hCGβ (99/650) for hCGβ-mAbs (**a**) and hCG (99/688) for holo hCG-mAbs (**b**) (NRH). Reference Abs for epitopes β_1_–β_9_ and c_1_–c_4_ served as ^125^I-labeled detection reagents, respectively. Reaction profiles of the solid-phase ISOBMii mAbs with the detection reference mAbs were cross-matched to that of solid-phase reference mAbs the molecular epitope specificity of which had previously been defined [1]. Similar reaction profiles were interpreted as epitope identity or neighborhood of mAbs. **a** The compatibility patterns of pairs of mAbs do not only reveal epitope affiliation of single mAbs but also disclose hCGβ epitope arrangement in larger antigenic domains consisting of one or more epitopes. Abs directed against epitopes located within the same antigenic domain are generally mutually exclusive in hCGβ recognition, whereas those the epitopes of which are located in different domains are compatible. Three major antigenic domains were identified on hCGβ: (1) the domain on the tips of hCGβ loops 1 + 3 encompassing epitopes β_2_–β_6_ (2) the cystine knot associated domain including hCG specific epitope β_1_, hCGβ + hCGβcf specific epitope β_7_, and a structurally related hCGβ-only specific epitope epitope β_14_ located on core hCGβ1-112 and characterized by a single mAb, and (3) hCGβCTP epitopes β_8_ and β_9_ remote from the other domains. MAbs against all hCGβ loops 1 + 3 associated epitopes β_2_–β_6_ are compatible with the hCG-specific cystine knot-associated epitope β_1_ and vice versa. Within antigenic domains not all epitopes can be discerned by distinct reaction profiles. As an example, although β_1_ and β_7_ show identical patterns in sandwich assays and are not compatible with each other, they are definitely recognizing different but adjacent epitopes as β_1_-mAbs are pan-hCGβ-mAbs recognizing a broad spectrum of hCG-variants and in contrast β_7_-mAbs are highly selective for hCGβ + hCGβcf and would not recognize, e.g., hCG (see, e.g., DB-RIA, Fig. 4). A second example are mAbs against epitopes β_4_ (ISOBM-419 and ISOBM-445) and β_5_ (ISOBM-428, ISOBM-429, ISOBM-430, ISOBM-431, and ISOBM-442) having an identical compatibility profile, i.e., nicely work with mAbs against epitopes β_1_ and β_7–9_ but not with β_2_–β_6_. These epitopes can be discerned by their variant recognition profiles whereby β_4_ mAbs are specific for hCG (≤1 % cross-reactivity with hLH) and β_5_ mAbs strongly cross-react with hLH (>>1 %) in titration and competitive RIA (Fig. 4). β_3_-mAbs, although showing a similar reaction pattern as other mAbs directed to hCGβ loops 1 + 3 associated epitopes (β_2_, β_4_, β_5_, and β_6_), seems to be remote from the free subunit specific epitope β_6_ and not compatible with hCGβCTP113-116 located epitope β_9_ at the beginning of hCGβCTP. Such spatial vicinity between the hCGβCTP and hCGβ loop 3 has already been postulated previously [72]. As expected, the epitope of β_8_-mAbs located at the very carboxyl-terminal end of hCGβ (aa hCGβ141-144; [24]) is compatible with all other epitopes. In the first ISOBM TD-7 WS, a new epitope β_14_ was observed represented by a single mAb (ISOBM-267) that exclusively recognized core hCGβ [1] and that now appeared compatible with all hCGβ locate epitopes except for epitope β_1_, thus seems to be remote from any other core hCGβ epitope. ISOBM-406 according to its sandwich pattern (no compatibility with cystine knot epitopes β_1_ and β_7_) seems to be cystine knot associated. **b** c-mAbs show variant reaction patterns among themselves. The heterodimeric epitopes c_1_–c_3_ are located in the same antigenic domain thus are not compatible with each other. c_4_ is clearly remote from that domain as as it is compatible with c_1_ to c_3_-mAbs. ISOBM-433 recognizes a previously structurally not defined epitope that is highly hCG specific as is ISOBM-446 (epitope c_3_) (Fig. 4). ISOBM-389 a highly hCG specific c-mAb that according to BIAcore® analyses rapidly dissociates (*K*_d_ = 13E−03), ISOBM-397 and ISOBM-418 (very low affinity in FRET analyses) did not perform well as capture mAbs in this type of assay and were negative throughout (not shown). Reactions classified as positive (mean + 2 standard deviations) are depicted as closed squares. Noncompatible mAb pairs are shown as white *squares*

IRMA-like sandwich assays were performed to confirm preliminary Ab epitope classifications by specificity assays and to determine epitope localization by comparison with reference mAbs. Characteristic reaction patterns were observed when solid-phase bound ISOBM-mAbs were tested for their ability to sandwich hCG or hCGβ with the panel of reference mAbs directed against epitopes β_1_–β_9_ (Fig. 6a) and c_1_–c_4_ (Fig. 6b). Patterns observed agreed with previously determined epitope locations for the reference mAbs [28, 38].

Compatibility of Ab pairs in sandwich assays indicated that their epitopes were spatially distinct, e.g., epitopes β_1_ versus β_2_–β_6_ and vice versa (Fig. 6a). Identical or highly similar compatibility patterns of Abs to that of reference or other mAbs indicated recognition of identical or of neighboring epitopes within the same antigenic domain: e.g., the cystine knot-related epitopes β_1_ and β_7_, or epitopes β_2_, β_4_, and β_5_ on hCGβ loops 1 + 3. Epitopes within a particular antigenic domain can be easily discerned by cross-reactivity patterns with hCG variants and LH from various species [1]. Thus, although β_1_- and β_7_-mAbs show identical compatibility patterns in sandwich assays and are not compatible with each other (Fig. 6a), they recognize different but spatially adjacent cystine knot-related epitopes reflected by differing variant recognition patterns: β_1_-mAbs recognize a broad spectrum of hCG-variants whereas β_7_-mAbs are highly selective for hCGβ, hCGβn ± hCGβcf and do not recognize hCG (Fig. 4).

##### Antigenic domains and epitope maps of hCG and hCGβ (INN)

**Fig. 7:**
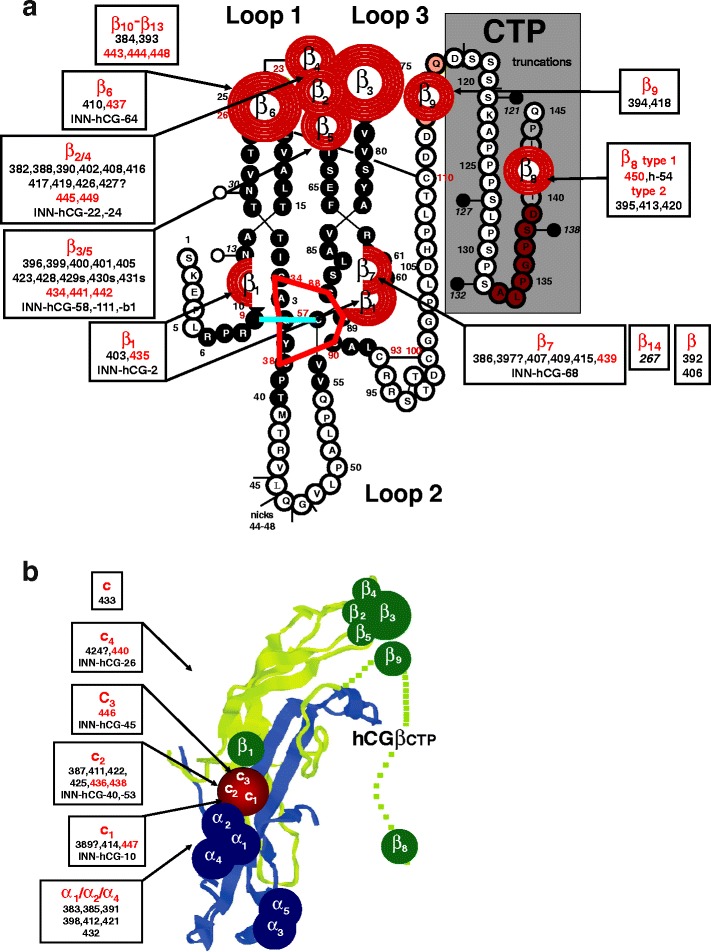
Epitope maps of hCG, hCGβ, and variants (INN) (modified according to [1], with permission) were previously constructed based on the epitopes recognized by the reference mAbs. The identification of reference mAb epitopes was performed by direct binding, competitive and sandwich RIA and ELISA with hormones of various species, hormones subunits, metabolic breakdown products, and synthetic peptides (for reviews, see [1, 22]). Furthermore, on the basis of molecular modeling of crystallographic data of hCG and subsequent mutational analyses to assign epitopes to particular amino acids, epitopes of reference mAbs and, by comparison, epitopes of ISOBM-mAbs could be superimposed on the molecular model of hCGβ. **a** Assignment of ISOBM-mAbs to epitopes on the molecular model of hCGβ/hCGβn/hCGβcf/hCGβCTP. Reaction profiles of the ISOBMii mAbs in specificity and sandwich assays were compared to that of reference mAbs. It appeared that the most immunogenic region of hCGβ is determined by the peptide sequences that correspond to hCGβcf. In particular, the tips of beta-sheet loops 1 + 3 corresponding to hCGβ20-25 + 68–77 comprise the major antigenic domain (epitopes β_2_–β_6_) that is recognized by high affinity mAbs. The only hCG-specific epitope on core hCGβ is β_1_ located around the center of the molecule corresponding to part of the cystine knot (aa hCGβ10,60,89). Adjacent to epitope β_1_, the hCGβ/hCGβcf-specific epitope β_7_ is also located in this region (aa hCGβ61,89) [43]. Thus, pairs of antibodies against these two epitopes are not compatible in sandwich type assays (Fig. 6a) [24]. hCGβCTP epitopes β_9_ and β_8_ are located at either end of the hCGβCTP, whereby β_9_ might be close to epitope β_3_ (Fig. 6a) [72]. **b** Epitope map of hCG. ISOBM-mAbs were assigned to epitopes on a ribbon representation of the molecular model of hCG [3]. hCGα and epitopes thereon are depicted in *blue*, hCGβ and its epitopes in *green*. Conformationally (**c**) dependent epitopes determined by the quaternary structure of hCG are shown in *red*. Note the major antigenic clusters of epitopes on the top of beta sheet loops 1 and 3 of hCGα (α_1_/α_2_/α_4_ and α_3_/α_5_) and of hCGβ (β_2_– β_5_), the central cystine knot-based epitope cluster encompassing highly hCG-specific β_1_ and c-epitopes (c_3_), the latter having a share on loop 3 of hCGβ, that in turn are confluent with the α_1_/α_2_/α_4_ epitope cluster. The hCGβCTP epitopes are located on both of its ends at aa hCGβ113-116 (epitope β_9_) and aa hCGβ133-144 (epitope β_8_)

Results of the three approaches for epitope typing are summarized in Table 5. In Fig. 7, the ISOBM-mAbs are assigned to the three-dimensional epitope maps of hCGβ (a) and hCG (b), which were established with the reference mAbs previously [1]. The Abs grouped according to epitope recognition are listed in Table 8 in Appendix.

**Table 5:**
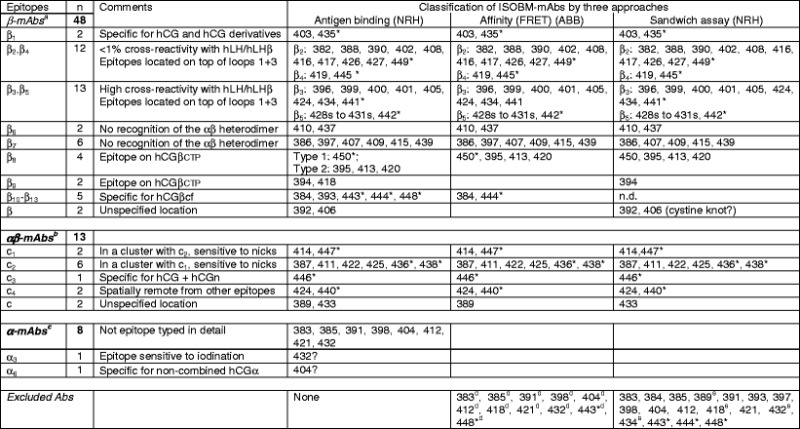
Epitope assignment of the ISOBM-Abs using three approaches

^a^Fourteen epitopes, 13 of which recognized by ISOBM-mAbs

^b^At least four epitopes; four plus one recognized by ISOBM-mAbs

^c^Seven epitopes; these ISOBM-mAbs were not characterized in detail

^d^Specificity based on affinity for hCG, hCGβ and hCGβcf could not be determined

^e^These four ISOBM-mAbs did not perform well as capture reagents

*Reference mAbs

In the ISOBM panel, 48 out of 69 were β-Abs. The major antigenic domain on hCGβ located on the tips of the neighboring β-sheet loops 1 and +3 encompassing aa hCGβ20-25 and 68–77 (epitopes β_2_–β_6_) was recognized by 27 of the β-Abs. Of these, 12 Abs recognize epitopes β_2_ or β_4_ (β_2/4_), 13 epitopes β_3_ or β_5_ (β_3/5_), and 2 epitope β_6_. Epitopes β_2_–β_5_ are pan hCG specific, i.e., present on hCG, hCGn, hCGβ hCGβn, and hCGβcf (Fig. 4a), whereas β_6_ is present only on hCGβ, hCGβn, and hCGβcf.

The cystine knot-associated antigenic domain comprises a number of epitopes that are recognized by 10 of the 48 β-mAbs (including ISOBM-397 results of which are ambiguous). ISOBM-267 that is hCGβ specific and its epitope cystine knot-related (epitope β_14_) was used as a control mAb: The pan-hCG epitope, β_1_ (hCGβ Arg10, Arg60, and Gln89), which is not shared by hLH, was recognized by two ISOBM-mAbs (ISOBM-403 and reference mAb ISOBM-435). It is spatially close to epitope β_7_ (hCGβ Asp61 and Gln89) against which six mAbs (including ISOBM-397) were directed. MAbs classified as β_7_ recognize either hCGβ + hCGβn + hCGβcf or mainly hCGβ + hCGβn (ISOBM-407). The cystine knot-related epitope β_10_ recognized by reference mAb ISOBM-448 is hCGβcf specific. One mAb (ISOBM-406) reacted with a not specified cystine knot epitope (Fig. 6a). This mAb is of restricted pan-hCG specificity and does not recognize hCGβcf (Fig. 4a).

In addition to the above-mentioned mAb ISOBM-448 (cystine knot related epitope β_10_), 4 of the 48 β-Abs recognize epitopes located on hCGβcf only (epitope β_11_, ISOBM-384 and ISOBM-444; epitope β_13_, ISOBM-443; and one non-coded hCGβcf epitope, ISOBM-393).

Six of the 48 β-Abs are directed against the hCGβCTP. The linear antigenic region (aa hCGβ137–144; epitope β_8_) at the very end of the hCGβCTP is recognized by four mAbs; type 2). Three of these (ISOBM-395, ISOBM-413, and ISOBM-420) are mAbs against epitope β_8,2_ recognizing glycosylated hCGβ much better than the nonglycosylated synthetic peptide. Thus, epitope β_8,2_ might be influenced by glycans on Ser132 and/or Ser138 [1, 39]. One mAb (ISOBM-450; epitope β_8,1_) recognizes both antigens to the same extent. Two mAbs, 394 and 418, may be directed against epitope β_9_. One β-mAb (ISOBM-392) could not be classified but it does not seem to be located on hCGβCTP (Figs. 4a and 7a).

Thirteen of the 69 mAbs reacted with c-epitopes: c_1_ (*n* = 2), c_2_ (*n* = 6), c_3_ (*n* = 1), c_4_ (*n* = 2), c (*n* = 1; ISOBM-433; new noncoded c-epitope). One c-mAb could not be classified (ISOBM-389).

Eight out of 69 mAbs are directed against hCGα. Six recognize assembled and one, ISOBM-404, which has been prepared by immunization with hCGα (Stenman et al., unpublished data), recognizes only free hCGα. The exact molecular localization of the hCGα mAbs was not elucidated (Figs. 4b and 7b).

## Discussion

### Topography of hCG epitopes

#### Epitopes and antibodies

By definition, epitopes are molecular structures dependent on the existence of complementary Abs. Not the entire surface of a glycoprotein like hCG is antigenic. Against certain molecular areas no Abs exist as they are immunologically inert, e.g., due to insufficient T cell help, or sterically not accessible due to protein folding or shielding by glycans. In contrast, other areas representing structurally inherent epitopes, which are characterized by high solvent accessibility and high protrusion indices, are often sites of Ab recognition [40]. hCGβ cystine knot-associated residues Arg10 and Gln89 (epitopes β_1_ and β_7_), hCGα loop 1 residues Pro16, Phe17, and Phe 18 (epitopes α_1_, α_2_,and α_4_) and the antigenic domain on hCGβloops 1 + 3 comprising aa 20–25 + 68–75 (epitopes β_2_–β_6_) all bulge away from the molecule forming prominent surfaces that are the major antigenic domains of hCG [3, 22, 37, 41–43]. There is a good chance that irrespective of the immunized species these molecular structures will be recognized as epitopes [38]. For example, the immunodominant antigenic domain on top of hCGβ beta-sheet loops 1 and 3 is recognized by Abs derived from mice and sheep as shown in the present study and interestingly by Abs from humans and rabbits (PB, unpublished observations). Moreover, hLH cross-reactive mAb B206 directed against an epitope within this cluster, presumably epitope β_3/5_, inhibited 40–90 % of the binding of human antisera to hCG [44].

The definition of epitopes by Abs and recognition of the multitudes of possible amino acid combinations within an inherently antigenic structure/domain is dependent on and restricted by the combinatorial repertoire of the VDJ and VJ immunoglobulin heavy and light chains gene segments, respectively, and the cellular capacity to mature the paratope of a given Ab to optimally fit the antigenic surface. This repertoire of Ab specificity varies with individual immune responses, haplotypes, and species. Not every amino acid combination within an antigenic domain will therefore be recognized by Abs of any individual or species. Thus, the repertoire of Ab specificities and corresponding epitopes within an antigenic domain is very large but still somewhat restricted as shown by the present and previous studies. For example, the antigenic domain on hCGβ loops 1 + 3 is recognized by large panels of Abs that differ slightly in hCG variant recognition, hLH cross-reactivity, affinity, etc. This has been shown to be due to variability in amino acid recognition within the antigenic domain [1].

It is striking that this antigenic region, aa hCGβ20–25 + 68–75 on the tips of loops 1 + 3, comprises 16 amino acids, a number that reasonably well corresponds to the surface covered by a single complementary paratope of an Ab whereby two to three amino acids that vary from Ab to Ab provide most of the binding energy and fine specificity [45]. Consequently, dozens of ISOBM-mAbs and Abs of other panels directed against hCGβ loops 1 + 3 epitopes β_2_–β_5_ do not behave uniformly in their recognition of the approximately 15 potential contact amino acids composing discontinuous epitopes, even though they cover more or less the same surface with their paratope [43]. Thus, all differences in affinity, specificity, and hLH cross-reactivity of numerous antibodies directed against this major antigenic region seem to have their basis in variability of preferential recognition of a few amino acids, providing binding energy within very similar or even identical sets of amino acids covered by the Abs’ paratopes.

The surface area of an epitope that is covered by a cylinder-like antigen binding site of an Ab is approximately 700 Å^2^ in size [38, 46], whereby the radius of the antibody binding domain is 8–10 Å and the radius of the epitope covering area is 15 Å irrespective of Ab specificity [45]. X-ray crystallography studies revealed that core hCG, i.e., hCG without hCGβCTP, has a length of 75 Å and a width of 30–35 Å [3, 47] corresponding to a surface area of approximately 8,200 Å^2^. As some regions on assembled hCGβ, such as the stems of β-sheet loops 1 + 3, are not recognized by any anti-hCG-mAbs [1, 18, 48], the total epitope-covered area on core hCG could be in the range of 5,000 Å^2^ theoretically accommodating simultaneous binding of up to seven Abs to spatially independent epitopes. The minimal spatial requirement for sterical compatibility of two mAbs is that the respective epitopes are approximately 20–30 Å apart. In fact preliminary experiments showed that at least five radiolabeled mAbs against epitopes β_1_ + β_3_ + α_2_ + α_3_ + c_4_ were able to bind to core hCG simultaneously [38].

#### Glycosylation and epitopes

With two exceptions, glycosylation has little effect on hCG’s immunological make-up, although the glycans, which are hydrophilic in nature and thus surface exposed, represent approximately 30–35 % of its total molecular mass. The exceptions are glycans at the very end of hCGβCTP and in the stem region of hCGβ loop 1. The 14 epitopes on core hCG, which is lacking hCGβCTP, are dependent on the protein backbone. Neither desialylation, deglycosylation [48], partial natural deglycosylation as in the case of the metabolic product hCGβcf [49], nor intense glycosylation as shown with highly acidic p*I* variants of pregnancy- and tumor-derived hCG have essential effects on Ab recognition by the reference mAbs [17, 18]. In addition, the number and the relative spatial location of epitopes do not differ between the isoforms [1, 18, 48].

The peptidic stem region of assembled hCGβ loop 1, which accommodates the two large N-linked glycans at hCGβAsn13 and Asn30 that are spatially near the hCGα glycan at Asn52 [3], is not recognized by any mAb in the panels of anti-hCG-mAbs of the previous and the present study. Thus, the immune response seems to be attenuated by the N-linked glycans in this region of hCGβ loop 1 [1, 18, 48].

A mAb (B152) that was not included in this study recognizes hCG with a core-2 O-glycan at Ser 132 and surrounding peptide structures [50, 51]. Its epitope, which we termed β_8,3_, is spatially related to epitope β_8,2_ that also seems to be influenced by the glycans on Ser 132 and/or Ser 138 [1, 29].

Some hCG assays have been claimed to underestimate hCG-h [52]. However, these results have been obtained with an hCG-h preparation that also was completely nicked (C5) [39]. Thus, the results most probably reflected failure to recognize hCGn rather than hyperglycosylated hCG.

#### Epitopes on assembled and/or free hCGβ (β_1_–β_9_, β_14_) and hCGβcf only (β_10_–β_13_)

The immunodominant structure of hCG and hCGβ-related molecules is the molecular region corresponding to hCGβcf, which has lost its N-terminus, the long loop 2, most of its N-linked carbohydrate antennae, and the hCGβCTP with all O-linked glycans but has retained its protein backbone configuration [53]. Thus, numerous mAbs against epitopes β_1_–β_7_ recognize hCGβ, hCGβn, and hCGβcf. However, one mAb (ISOBM-407) did not react with hCGβcf.

The epitopes on assembled and/or free hCGβ (β_1_–β_9_, β_14_) are located in three molecular regions: (1) hCGβ cystine knot, (2) tips of hCGβ loops 1 + 3, and (3) hCGβCTP.

The cystine knot-associated antigenic domain includes epitope β_1_ involving aa hCGβArg10 + Arg60 and possibly Gln89 that sterically are in close proximity to each other [42, 43]. hCGβArg10 and Gln89 are unique to hCG and not shared by hLH. This presumably explains why epitope β_1_ is highly specific for hCG and its variants and therefore is not present on hLH or hLHβ [26]. Due to its superior specificity, it is highly valuable for hCG/hCGβ-variant measurement by immunoassay with no interference by hLH or hLHβ [1].

The assumed location of epitope β_7_ on hCGβ, hCGβn, and hCGβcf is based both on mutational analyses and vicinity analysis by sandwich assays: It is associated with the cystine knot, present on hCGβcf, and Asp61 and Gln89 have a role in this epitope. Thus, in sandwich type assays, β_7_-mAbs are not compatible with β_1_-mAbs (Fig. 6a) [1, 22, 24].

MAbs against the cystine knot epitope β_7_ recognize hCGβcf in addition to hCGβ. ISOBM-407 is an exception to this, although other parameters match with epitope β_7_, it shows an exceptionally low cross-reactivity with hCGβcf (Fig. 4) and thus seems to be suitable for measurement of hCGβ in urine in the presence of high levels of hCGβcf. The assignment of hCGβ specific epitope β_14_ to the cystine knot antigenic domain is based on circumstantial evidence as mAb ISOBM-267 defined in the First ISOBM TD-7 WS to recognize epitope β_14_ is not compatible with hCGβcystine knot-related epitope β_1_ but with all other hCGβ-related epitopes (Fig. 6a). Two hCGβcf epitopes β_10_ and β_12_ are also cystine knot-associated (PB, unpublished data). An additional cystine knot-related epitope is represented by mAb ISOBM-406.

Antibodies directed against the major hCGβ antigenic domain on loops 1 and 3 are of significantly higher affinity compared to those against other antigenic regions of hCGβ[1, 21, 54]. MAbs against epitopes β_2_–β_5_ recognize a wide spectrum of hCG and hCGβ-related variants (hCG, hCGn, hCGβ, hCGβn, and hCGβcf) [1, 17, 18]. MAbs against epitopes β_3_ and β_5_ additionally react well with hLH and hLHβ, whereas epitopes β_2_ and β_4_ are specific for hCG and hCGβ variants (<1 % hLH and hLHβ cross-reactivity) and thus highly suitable for specific measurement of hCG and hCGβ variants (Fig. 4) [1, 26].

In summary, β-epitopes located on the protein core hCGβ1-112 are discontinuous in nature, determined by the tertiary protein structure, present on hCGβcf, and arranged in antigenic domains associated with the cystine knot and on the tips of loops 1 + 3. MAbs directed against these epitopes are of adequate affinity and suitable for immunoassay applications.

hCGβ-related epitopes not determined by hCGβcf or core hCGβ_1–112_ are located in two major regions on the hCGβCTP (aa hCGβ113–145). The immunodominant linear antigenic region at the very end of the hCGβCTP consists of aa hCGβ133–144 and encompasses epitope β_8_ that is composed of epitope variants β_8,1_, β_8,2_, and β_8,3_) [29, 55]. It partially seems to be influenced by glycans on Ser132 and/or Ser138 (epitopes β_8,2_ and β_8,3_) [29] [1, 50]. One mAb in this WS (epitope β_8,1_; ISOBM-450) and four mAbs in the First WS recognized nonglycosylated synthetic peptides and glycosylated hCGβ equally [1]. Epitope β_9_ at aa hCGβ113–116 [21, 24] was recognized by two mAbs (Fig. 7a) whereby ISOBM-394 was of high and ISOBM-418 of very low affinity (Fig. 5).

When immunizing with the glycoprotein hCG, the vast majority of antibodies will be generated against composite epitopes on hCGα or the core region of hCGβ (aa 1–112) but only rarely against linear peptide sequences of low structural order like the hCGβCTP. MAbs against hCGβCTP are generally of fairly low affinity. Nevertheless, they are used in diagnostic sandwich-type immunoassays as they do not cross-react with hLH (Fig. 4).

#### hCGα epitopes (α_1_–α_7_)

In the panel of ISOBM-mAbs, 8 of 69 recognize hCGα epitopes. One of these mAbs, ISOBM-404, seems to be specific for free hCGα, and it is speculated that it might recognize the sequence hCGα33-42 on the single loop 2. As no reference hCGα-mAbs (Table 2) were included, a detailed assignment of epitopes was not possible.

#### Epitopes on the hCG αβ-heterodimer (c_1_–c_4_)

At least four epitopes (c_1_–c_4_) are present only on hCG ± hCGn but not on either free subunit or hCGβcf [21, 26, 28]. Detailed analysis of hCG and hCGn recognition by the ISOBM-mAbs was performed by liquid chromatography mass spectrometry (LC-MS/MS) (see accompanying publication by H. Lund). Epitopes c_1_ (reference mAb INN-hCG-10) and c_2_ (reference mAbs INN-hCG-40 and INN-hCG-53) are (1) dependent on intact hCG and thus sensitive to nicking of assembled hCGβ loop 2, (2) not compatible in sandwich-type assays with the cystine knot-related hCGβ epitope β_1_ (aa hCGβArg10 + Arg60 and possibly Gln89) [38], and (3) incompatible with mAbs recognizing epitope cluster α_1_, α_2_, and α_4_ [38] on loop 1 in the region of aa hCGα13-22. Amino acids hCGβ44-48 in loop 2 and hCGα loop 1 have been shown by X-ray crystallography to be in close proximity as the subunits are assembled in a head-to-toe fashion [3]. It is striking that in sandwich assays c_1_-mAbs show identical reactivity patterns as α_1_- and α_2_-mAbs reflecting sterical epitope relatedness [28, 38].

MAbs against epitope c_3_ are sterically related to epitope c_2_, highly specific versus hLH as well as non-combined intact and modified subunits (<1 % cross-reactivity), not influenced by nicking of assembled hCGβ loop 2, and thus recognize hCGn and hCG equally [1, 56](Fig. 4). They are therefore highly suitable for simultaneous measurement hCG and hCGn (Table 6).

**Table 6:**
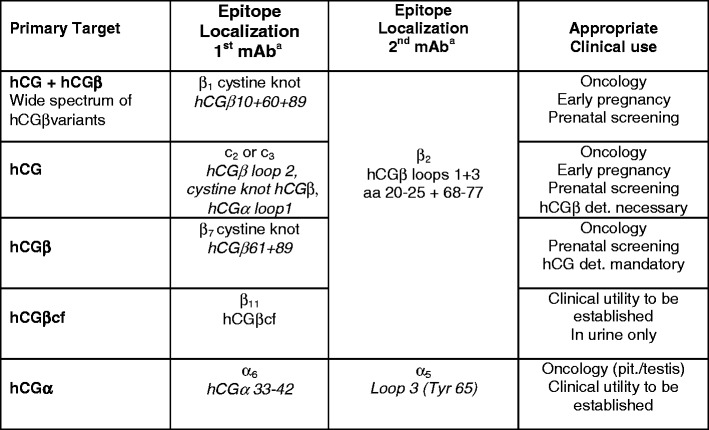
hCG and/or hCG-variants measurements: candidate epitopes for sandwich methods (modified according to [1])

^a^Candidate mAbs for the respective epitopes are listed in Figs. 4, 6, 7, and Appendix 2

The exact molecular localization of epitope c_4_ has not been resolved yet. It is present on hCGn and hLH, remote from and thus sterically compatible with all other c-epitopes and to a minor extent determined by hCGβ as shown by low cross-reactivity [1, 26, 38]. A variant of the c_4_-epitope represented by ISOBM-424 (=ISOBM-279, First ISOBM TD-7 WS) that is not shared with hLH (cross-reactivity <0.1 %) seems to exist. A presumably fifth highly specific c-epitope has been observed in sandwich assays wherein mAb ISOBM-433 is compatible with mAbs to c_1_–c_4_ (Fig. 6b). Its molecular localization is unknown. ISOBM-389 is a c-mAb that could not be epitope typed but, according to its specificity profile analyzed by LC-MS/MS, might be a c_2_ mAb (see accompanying publication by H. Lund).

## Method-specific recognition of hCG and hCG variants

Sandwich-type assays measuring hCG alone or in combination with free hCGβ and metabolites are used for detection of pregnancy, pregnancy-related disorders, trophoblastic disease, and various other female and male tumors [2]. Detailed knowledge of the epitopes recognized by the Abs used facilitates development of assays providing better comparability of the results between methods. It has been suggested that assays that are multifunctional with respect to clinical use should (1) recognize in an equimolar fashion hCG and hCGβ protein backbone and glycosylation variants, (2) not cross-react with hLH or derivatives, and (3) not be prone to signal blunting by non-measured variants, e.g., caused by excess hCGβcf leading to false low results [36]. This is a problem when hCG in urine is measured with sandwich assays utilizing a mAb against core hCGβ1-112 in combination with an anti-hCGβCTP mAb [57].

While assays measuring hCG and all hCGβ-related variants are useful as first line methods, for diagnosis of pregnancy and cancer, it is often advantageous to specifically measure only selected variants [2]. Thus, specific hCGβ assays are used for first trimester Down’s syndrome screening and also for diagnosis of testicular [58, 59] and nontrophoblastic cancers, 20–50 % of which produce only hCGβ but not hCG [60–62]. However, the concentrations are mostly low, and the assays used need to be highly sensitive. Assays for hCGβ that are intended for first trimester screening of Down’s syndrome need to be insensitive to interferences by an approximately 100-fold excess of hCG and tuned to measure fairly high concentrations. They are therefore of limited utility for the diagnosis of nontrophoblastic cancers.

Elevated plasma concentrations of hCGβ are reflected by high levels of hCGβcf in urine [63], and specific assay of this form has been used for diagnosis of nontrophoblastic cancer [62, 64, 65] and for the characterization of the First IRR for hCGβcf [9]. However, commercial assays are not available presently.

### Candidate epitopes for measurement of hCG and hCGβ

Assays specifically recognizing hCG, hCGβ, and related variants can be constructed using a combination of two pan hCGβ mAbs with identical specificity profiles [66], i.e., with one partner directed against epitopes β_2_ or β_4_ (the hCGβloops 1 and 3 domain) combined with a mAb-recognizing epitope β_1_ (the hCGβ cystine knot domain; Table 6).

In the two ISOBM TD-7 WSs, 50 of 96 Abs were shown to recognize hCG + hCGβ and 23 of these did not recognize hLH. Theoretically, any of the five mAbs directed against epitope β_1_ around the cystine knot could be combined with any of the 18 mAbs against epitopes β_2_ or β_4_ on loops 1 + 3 for construction of multifunctional assays. Epitopes β_1_ and β_2/4_ are shared by all important hCG and hCGβ protein backbone variants and glycosylation isoforms including hCG-h and hCGβ-h [17, 18]. MAbs against these two discrete epitopes are highly specific for hCG with <0.1 and <1 % cross-reaction for hLH for epitopes β_1_ and β_2/4_, respectively. No other epitope combination provided assays with equally wide and identical recognition of hCG and hCGβ variants and high specificity versus hLH.

While Abs recognizing these epitopes provide desirable specificity, variable affinity for hCG variants (Fig. 5) may cause nonequimolar recognition of hCG and hCG variants in different methods [6]. Although assay specificity can be predicted on the basis of mAb specificity profiles and epitope recognition [66], ultimate performance can only be evaluated with the final assay. An additional source of method variability in hCG measurement that cannot be fully predicted is that of Ab synergy, which may vary between different Ab pairs [67].

### Alternative epitopes for measurement of hCG and/or hCGβ and variants

Few manufacturers provide information about the epitope specificities of Abs used in their assays, but due to variable recognition of the First IRR preparations for hCG and variants, it is obvious that different epitope combinations are used in the major commercial assays [6]. In addition to the epitope combination β_1_–β_2/4_, other combinations are possible for the construction of assays for hCG and variants, e.g., epitopes β_8,1_ and β_2_, β_1_–α_5_, α_4_–β_2_, etc., but none of them will fulfill all three above-mentioned criteria. However, the frequently used β_8_ and β_2_ combination does not pose problems as long as serum specimen are measured that do not contain hCGβcf, truncated hCG or truncated hCGβ, or clipped hCGβCTP.

For selective measurement of hCG or free hCGβ or hCGβcf certain epitope combinations can be suggested: for hCG (no recognition of hLH or noncombined subunits), a mAb against epitope c_2_ or c_3_ can be combined with one against β_2/4_ (Table 6). Alternatively, β_1_–α_3_ combinations [66] or β_2/4_ combined with a tracer mAb against an α epitope are possible [68]. These designs eliminate cross-reactions with free subunits but are sensitive to interferences by free subunits and hCGβcf. For measurement of free hCGβ, a mAb to epitope β_7_ or β_14_, and for hCGβcf, a mAb to epitope β_11_ can be combined with one to epitope β_2/4_. For hCGα, combinations of mAbs against epitopes α_6_ and α_5_ are recommended [69] (Table 6).

A unique mAb coded B152 is used for the measurement of hCG-h that carries a core-2 glycan on Ser132 located on hCGβCTP [20]. However, the clinical utility of assays using this mAb remains to be established [70].

### Future perspectives: harmonization of hCG and/or hCGβ and variant measurement

Considerable reduction in between-method and between-laboratory variability in results can be achieved by a number of measures: (1) the establishment and usage of a clear nomenclature of hCG and its variants [1, 8]; (2) endorsement of that nomenclature to define what hCG-assay measure [1, 8, 60]; (3) characterization of diagnostic assays with the new six First IRRs calibrated in SI units that were adopted by WHO for immunoassay standardization [6]; (4) standardization of methods with the highly pure new WHO Fifth IS for hCG encoded 07/36,4 which is identical to the First IRR for hCG 99/688; (5) harmonization of mAb epitopes used in diagnostic methods for hCG, hCGβ, and their variants; and (6) the establishment of reference methods for the various forms of hCG [8], which will be supported by the detailed knowledge on Ab epitope recognition reported in the present study.

### Electronic supplementary material

Below is the link to the electronic supplementary material.

ESM 1 (PDF 20194 kb)

## Figures and Tables

**Table 7:**
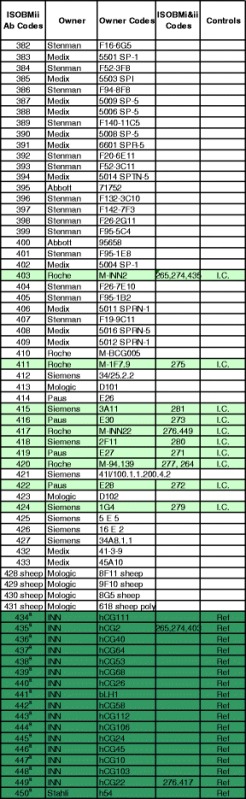
ISOBM-TD-7 Abs submitted to Dr. Kjell Nustad

*I.C.* Internal control

^a^Reference antibodies

**Table 8:**
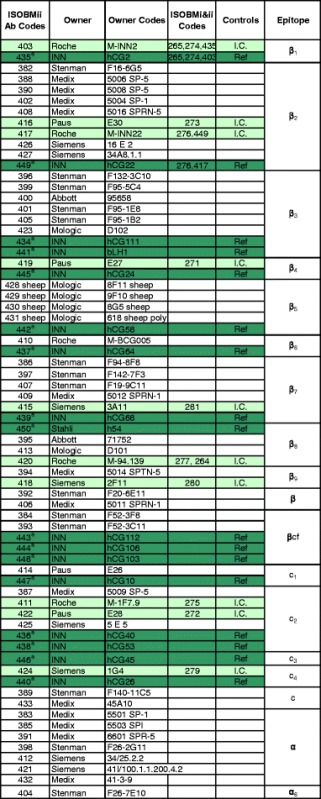
Abs grouped according to epitope recognition

*I.C.* internal control

^a^Reference antibodies
