# Supplementary material for: Candidate epitopes for measurement of hCG and related molecules: the second ISOBM TD-7 workshop
Source: Tumour Biol. 2013 Sep 26;34(6):4033–57. doi: 10.1007/s13277-013-0994-6 (PMC3858614; doi:10.1007/s13277-013-0994-6)
Supplement: Supplementary file 1 — (PDF 20194 kb) [file 13277_2013_994_MOESM1_ESM.pdf]

Candidate Epitopes for Measurement of hCG and Related Molecules: The 2nd ISOBM TD-7 Workshop

Tumor Biology

P Berger*, E Paus, P M Hemken, C Sturgeon, W W Stewart, J P Skinner, L C Harwick, S C Saldana, C S Ramsay, K R Rupprecht, K H Olsen, J-M Bidart , U-H Stenman

*Corresponding author:

Peter Berger, Ph.D., Professor
Institute for Biomedical Aging Research
University of Innsbruck
Rennweg 10
A6020 Innsbruck
Austria

E-mail: peter.berger@uibk.ac.at

Online Resource 1

GPC-HPLC (ABB)

The mAbs were analyzed for purity by gel permeation chromatography using a Waters (Millford, MA) HPLC system. A TSKgel G3000SWxl column (Tosoh Biosciences #08541, King of Prussia, PA) was operated at ambient temperature with a flow rate of 1.0 mL/min using 100 mM phosphate buffer pH 7.2 containing 150 mM NaCl. Antibody samples with concentrations greater than 1.0 mg/ml were diluted to 1.0 mg/mL using the mobile phase and 10 µg of each sample was injected in triplicate. For samples containing less than 1.0 mg/mL, a volume containing 10 µg of mAb was injected in triplicate. Sheep antibody ISOBM-430 was diluted to 0.5 mg/mL and 5 µg injected in triplicate. Of sheep Abs ISOBM-428, 429 and 431, 5 µg of each sample was injected in triplicate. Absorbance was monitored at 280 nm.

Online Resource 2

SDS-PAGE (ABB)

The mAbs were also analyzed for purity by SDS-PAGE under reducing conditions on a Bio-Rad (Hercules, CA) Criterion electrophoresis apparatus operated at 150 V/gel with 12% Bis-Tris Criterion gels (Bio-Rad, #345-0118). Samples were mixed with 4X XT sample buffer (Bio-Rad, #161-0791) containing 2-mercaptoethanol (Bio-Rad, #161-0710) and heated in a boiling water bath for 3 min. Sample loads ranged from 1.5 µg for the sheep antibodies to 3.0 µg for mouse antibodies. The gels were stained with Coomassie Brilliant Blue R-250 (Organic Dyestuff Corp, OrcoAcid, East Providence, RI) and destained in methanol/acetic acid solutions followed by a water wash. Gels were scanned using a Bio-Rad GS-800 Imaging Densitometer. Detection parameters included a 250 rolling disk background, 750 sensitivity, 2.5 width, 0.01 minimum density, 0.5 filter, 5.0 shoulder and a size of 15. Molecular weights were estimated by comparison with the migration rates of broad range standards (Bio-Rad, #161-0317).

Online Resource 3

SDS-PAGE (NRH)

Electrophoresis was performed in Novex precast 4 – 12 % polyacrylamide gels (Novex, SanDiego, USA) containing sodium dodecyl sulphate. Aliquots of each antibody in 1 % SDS (BioRad Laboratories) was boiled for 2 min before loading 8 µg to the gels. After electrophoresis in the

presence of SDS without reducing conditions, gels were stained with Coomassie brilliant blue (Bio-Rad Laboratories, CA, USA).

Online Resource 4

Determination of antibody isotype (ABB, NRH)

The class and subclass of mouse mAbs displaying double heavy or light chains in SDS-PAGE or more than one cluster of bands by IEF analysis were determined using Iso*Strips* (Roche, #1493027, Indianapolis, IN) in accordance with the method provided by the manufacturer (ABB).

Online Resource 5

Isoelectric Focusing (IEF) (ABB)

A Phastsystem™ separation, control and development unit (GE Healthcare, Uppsala, Sweden) and pH 3–9 gradient gels (GE Healthcare, # 17-0543-01) were used for IEF of Abs. Gels were stained with silver nitrate (GE Healthcare, PhastGel Silver Kit Plus One, #17-1150-01) as described by the manufacturer. Sample lanes were bracketed by the appropriate pI markers (GE Healthcare, Broad Range pI calibration Standard, #17-0471-01). Gels were scanned using a Bio-Rad GS-800 Imaging Densitometer.

Online Resource 6

Mass spectrometry (MS) *(ABB)*

MW profiles were determined by MALDI-TOF-MS using PerSeptive Biosystems Voyager-DE. Electro Spray Ionization (ESI-MS) MS was performed with an AB SCIEX API QSTAR Pulsar i LC/MS/MS system to study post-translational modifications of the Abs.

All samples for mass spectrometry analysis were equilibrated at room temperature for 30 min and desalted using centrifuge filter or dialysis prior to MS analysis. For reduction of samples for the analysis of heavy and light chains, TCEP solution (Pierce) was added to a final concentration of 50 mM and incubated for 10 min at room temperature prior to desalting. Deglycosylation of N-glycans was performed with PNGase (Sigma) and O-deglycosylation with O-link enzyme cocktail (Prozyme). Samples were incubated overnight with enzyme at 37°C, prior to reduction and desalting.

MALDI-TOF-MS: Samples (0.4 µL) were applied to a MALDI sample plate and mixed with (0.4 µL) of sinapinic acid matrix solution, air dried, and loaded to MALDI instrument for analysis. The instrument

was calibrated using BSA standard, and operated in the positive, delayed extraction, linear mode using high mass MALDI-TOF-MS parameters.

ESI-MS: Desalted samples (20 µL) were transferred to an autosampler and 8 µL injected for analysis of intact mAbs. For analysis of TCEP-reduced, and/or deglycosylated and desalted samples, 2 µL of sample was injected.

Online Resource 7

Radiolabelling with ^{125}I (NRH)

Aqueous solutions of the carrier-free frozen concentrates of the six 1st IRRs of hCG and hCG-related molecules and hLH-I-1 AFP4345B were iodinated by the indirect IodoGen method (Pierce, Rockford, IL, USA). One to 10 µg of protein or peptide was iodinated with $Na^{125}I$ (Hartman Analytic GmbH, Germany) at an equal molar ratio of protein to iodine. Iodinated proteins were stored at –20°C in 50% ethylene glycol (Fluka Chemie Ag, Buchs, Switzerland). Specific activity was approximately 1200 Ci/mmol (44TBq/mmol) for all antigens.

Online Resource 8

Antibody specificity profiles determined by radioimmunoassay (RIA; NRH)

The main specificities of Abs were determined by DB-RIA with 125I-labeled hormones and hormone variants and with excess Ab (1 µg in 200 µl corresponding to 31.2 x 10^{-9} mol/L). Tubes with 100 µl of ^{125}I-hCG, ^{125}I-hCGn, ^{125}I-hCGβ, ^{125}I-hCGβn or ^{125}I-hCGβcf, (0.1 ng - 0.8 ng, approx. 50,000 cpm) in 0.05 mol/l Tris-HCl containing 0.1 mol/L NaCl and 0.1 % BSA, were incubated overnight at room temperature with 100 µl of increasing concentrations of the ISOBM-Abs (0.1 – 312 x 10^{-10} mol/L) diluted in the same buffer. Free and bound antigen was separated using sheep-anti-mouse antibodies (SAM) coupled to paramagnetic polymer particles (Dynabeads M280, Dynal, Oslo, Norway), by adding 100 µl of a 10 mg/mL solution. After 1 h shaking, the particles were washed three times with PBS containing 0.1% Tween, and bound radioactivity counted for 1 min in an automatic counter (LKB-Wallac). SAM was coupled to non-magnetizable polymer particles, resulting in 16 µg SAM per mg particles (4). The SAM antibodies reacted with all classes of mouse IgG with high specificity for Fc. Assays were performed in duplicate. The fraction bound to each antibody was expressed as percent net binding of the maximum binding obtained with that antigen. Non-specific binding was <900 cpm.

Online Resource 9

Competitive Ligand Analysis (CLA) (NHD)

The specificity of the ISOBM-Abs was analysed by competing the binding between ^{125}I-hCG and serial diluted Abs with fixed concentrations of the six 1st IRRs of hCG and hCG-related molecules and hLH (75/552) in a RIA format.

ISOBM-Abs were serially diluted (1:100 to 1:1.6*10^6) in assay buffer (50mM Phosphate, 50mM EDTA, 0.1% BSA, 0.01% sodium azide, pH 7.2) containing normal mouse serum (1:100) or normal sheep serum (1:400). One hundred µL of Ab and 100 µl of ^{125}I-hCG (10,000 cpm; 2.2 Ci/mmol) were incubated overnight at ambient temperature with 100 µL of competitor at fixed concentrations of 0.5 pmol/mL in assay buffer. Separation of bound and free radioactivity was achieved by anti mouse IgG (100µl, 1:20 in assay buffer) or anti sheep IgG (100µl, 1:40 in assay buffer) (Diagnostic Scotland, Law Hospital, Carluke, Lanarkshire, Scotland) followed by 100µl of 5% PEG 6000 (60 min, ambient temperature). Thereafter, 0.5mL assay buffer was added and the tubes centrifuged at 3000 rpm for 60 min. The supernatant was discarded and the pellet counted (1 min) in a gamma counter (LKB-Wallac). ^{125}I-hCG bound to the Ab at each dilution was expressed as net percentage of the total counts added. Non-specific binding was typically 300 cpm. Antibody dilutions were assayed in duplicate

Online Resource 10

Determination of cross-reactivity with hLH by titration RIA (NRH)

^{125}I-labelled hCG and ^{125}I-labelled LH were incubated overnight with increasing concentrations of ISOBM-Abs (0.05-175 x 10^{-10} mol/L). Free and bound antigen was separated using SAM coupled to paramagnetic polymer particles (Dynabeads). After 1 h incubation with continuous shaking the Dynabeads were washed 3 times.

Cross-inhibition with LH was estimated from the concentration of free antibody (in mol/l) needed to achieve half-saturation of LH vs. hCG, based on the method described by van Heyningen [34]. The actual antibody concentrations were determined at half of maximum binding of labelled antigen (titer), and corrected for the bindable fraction of each antigen. Total radioactivity added was approximately 50,000 cpm and non specific binding <800 cpm.

Online Resource 11

Competitive RIA (NRH)

ISOBM-Abs were diluted in PBS with 0.3 % BSA to achieve approximately 30 - 50 % maximum binding of radiolabeled hormone. In competition experiments, ^{125}I-labelled hCGβ (50,000 cpm in 100 µl) was incubated with mAbs over night in the presence or absence of increasing amounts of competing peptide, either *hCGβ135-145* (10^{-4} to 10^{-6} mol/L), *hCGβ109-145* (10^{-6} to 10^{-9} mol/L) or hCGβ (10^{-7} to 10^{-10} mol/L). Free and bound antigens were separated as described above. Measurements were performed in duplicates. Non-specific binding was <700 cpm.

Online Resource 12

Determination of Ab affinity by Forster Resonance Energy Transfer (FRET) (ABB)

For solution based equilibrium affinity measurements hCG, hCGβ and hCGβcf and hLH were labeled with Alexa488 (Invitrogen, Carlsbad, CA) at a dye to protein ratio of 8 to 1 antigen solution (1 mg/mL). The sample was incubated at room temperature for 3 h before purification using a size exclusion column (NAP-5, GE Healthcare, Bucks, United Kingdom) equilibrated with buffer containing 20 mM sodium phosphate, 150mM NaCl, pH 7.2. Incorporation ratio (IR) was estimated based on absorbance at 280 nm and 495 nm on a Cary 3G Spectrophotometer (Varian, Palo Alto, CA). An extinction coefficient of ε_{280} = 11,600 (M cm)$^{-1}$ was used for intact mAbs. For hCGβ and hCGβcf an extinction coefficient of ε_{280} = 5,280 (M cm)$^{-1}$ was used. A correction factor $\varepsilon_{280}/\varepsilon_{495}$ = 0.16 was used to account for absorbance of Alexa488 at 280nm. An extinction coefficient of ε_{495} = 71,000 (M cm)$^{-1}$ was used to determine the concentration of Alexa488. The incorporation ratio for intact hCG was 2.7, 2.5 for hCGβ and 1.0 for hCGβcf. Labeling of hLH (Fitzgerald Industries International, Acton, MA) with Alexa488 was performed following the same procedures. An extinction coefficient of ε_{280} = 21,700 (M cm)$^{-1}$ was used for LH. The IR for the labeled hLH was 1.0.

All Abs were labeled with black hole quencher (BHQ) dye (BHQ-10, Biosearch Technologies, Novato, CA) following the techniques for Alexa488 as described above. The incorporation ratio was determined by measuring absorbance at 280 nm and 518 nm. The extinction coefficient for Abs was ε_{280} = 217,500 (M cm)$^{-1}$ and for BHQ ε_{518} = 30,000 (M cm)$^{-1}$. The absorbance at 280 nm was corrected for BHQ absorbance using a factor $\varepsilon_{280}/\varepsilon_{518}$ = 0.67. Average IR for Abs labeled with BHQ was 3.3, the range 2 - 10, and for most Abs 2 – 6. ISOBM-431 was the only Ab with a ratio as high as 10.

Quenching and dissociation were determined according to Ruan, et. al. [35]. Briefly, each Ab was

serially diluted using a constant concentration of 50 pM Alexa488-labeled antigen at a starting concentration of 25 nM. Doubling Ab dilutions were made to prepare sixteen 1-mL samples for a final dilution factor of 2^{15} in the last sample (7 pM). The samples were incubated for 20 min in the dark at room temperature before measurement of fluorescence intensity. The quenching was calculated and used to determine the fraction of antigen bound and the number of free sites in each sample. A fit to the fraction bound as a function of free sites determines the reported dissociation constant K_d. The upper limit of detection is 50 nM, which is defined by the number of antibody binding sites in the first point of the titration experiment. Repeated measurements using this method indicate that dissociation constants are correct to within a factor of two.

Online Resource 13

Determination of Ab affinity by BIAcore® (NHD)

hCG (99/688), hCGβ (99/650) and hCGβcf (99/708) were diluted in acetate buffer, pH 4 (GE Healthcare, UK) and covalently bound to a BIAcore® CM5 sensor chip, which consists of a gold film on a glass support coated with a dextran film providing a hydrophilic environment, using an amine coupling kit (GE Healthcare UK). The continuous flow of antigens over the activated dextran was stopped at 400 resonance units (RU). Excess activated groups were blocked with ethanolamine. The second flow cell on the chip was likewise activated, not coated with antigen but blocked with ethanolamine and used as a blank control. ISOBM-Abs were diluted in HBS-EP running buffer (GE Healthcare UK) and six concentrations (0.5 to 5 µg/mL), passed over the antigen coated chip. This was followed by a timed interval to allow dissociation measurement. Binding responses were expressed in RU and were proportional to the molecular mass on the chip surface. Affinity constants for each of the Abs were calculated using the Biaevaluation software (GE Healthcare UK). Regeneration of the chips was achieved by injecting glycine buffer pH 2.5 until the baseline returned to the level before binding of Ab.

Online Resource 14

Compatibility of Antibody Pairs in Sandwich RIA (NRH)

Antibodies (100 µl, 0.5 µg /well) were adsorbed to Maxi Breakapart microtiter plates (Nunc, Roskilde, Denmark), in 0.1 mol/l sodium dihydrogen phosphate buffer pH 4.3. The microtiter plates were incubated under humidified conditions at 37 ^{o}C for 20 h, washed twice with PBS containing 0.05 %

Tween 20, and blocked for 20 h at room temperature under humidified conditions with 300 µl blocking buffer (0.05 mol/l Tris-HCl buffer pH 7.0 containing 10 g/l BSA, 60 g/l D-Sorbitol, and 0.5 g/l NaN_3, (Sigma Chemical, St.Louis, MO). After incubation the plates were aspirated and kept dry at 4 °C until use.

hCG (99/688) or hCGβ (99/650) (1 nmol/L each) in PBS containing 0.3 % BSA (100 µl,) was added to the MaxiSorp plates coated with Abs and incubated with shaking for 1 h. The wells were washed three times before incubating with 100 µl of ^{125}I-labelled mAbs in PBS-0.3% w/v BSA (50,000 cpm) for 1 h. Wells were washed 3 times and bound radioactivity counted.

Online Resource 15

ISOBM-Abs: Analytical Analyses by GPC-HPLC Tosoh TSK G3000 SWxl

Sixteen mAbs (ISOBM-411, 417, 428, 434, 435, 436, 438, 439, 440, 441, 442, 444, 445, 446, 448 and 450) contained additional peaks with an elution volume similar to that of albumin. ISOBM-431 and 442 had low signals, and consequently high background noise, so data for these samples should be treated with caution. Twenty of the Abs (ISOBM-382, 386, 392, 393, 399, 405 (Figure 3A), 407, 413, 414, 428, 435 (Figure 3E), 436, 437, 438, 439, 441, 442, 443, 445 and 449) exhibited greater than 5% aggregation. Eighteen Abs (ISOBM-391, 393, 394, 406, 408, 409, 411, 414, 416, 419, 422, 428, 434, 440, 441, 442, 448 and 450) exhibited greater than 5% combined low molecular weight peaks. Purities of <90% were observed for 22 Abs (ISOBM-386, 393, 394, 399, 406, 408, 409, 414, 416, 419, 422, 428, 431, 434, 436, 437, 438, 441, 442, 445, 449 and 450).

SAMPLE INFORMATION

Sample Name:	Albumin	Acquired By:	harw ilc
Sample Type:	Unknow n	Date Acquired:	8/26/2008 2:40:04 PM
Vial:	1:A,2	Acq. Method Set:	GPC3000 LCH Dual Wavelength
Injection #:	1	Date Processed:	8/28/2008 11:39:06 AM
Injection Volume:	10.00 ul	Processing Method:	ISOBM hCG Samples 082708
Run Time:	20.0 Minutes	Channel Name:	2487Channel 1
Sample Set Name:	GPC 082608 LCH Final	Proc. Chnl. Descr.:	280 nm

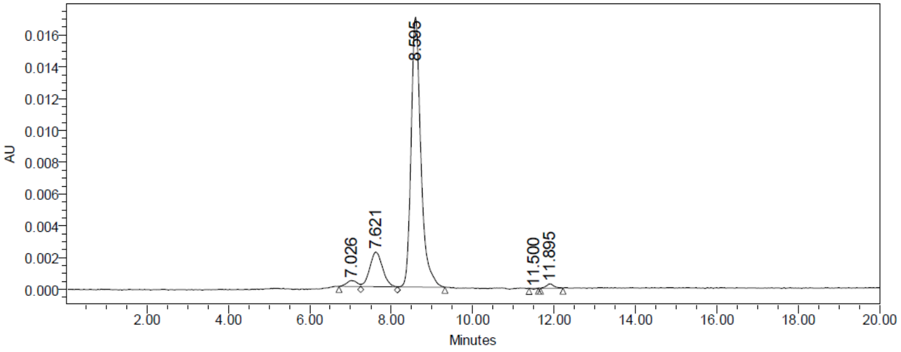

SAMPLE INFORMATION

Sample Name:	GFS	Acquired By:	harw ilc
Sample Type:	Unknow n	Date Acquired:	8/26/2008 3:42:45 PM
Vial:	1:A,3	Acq. Method Set:	GPC3000 LCH Dual Wavelength
Injection #:	1	Date Processed:	8/28/2008 11:39:06 AM
Injection Volume:	10.00 ul	Processing Method:	ISOBM hCG Samples 082708
Run Time:	20.0 Minutes	Channel Name:	2487Channel 1
Sample Set Name:	GPC 082608 LCH Final	Proc. Chnl. Descr.:	280 nm

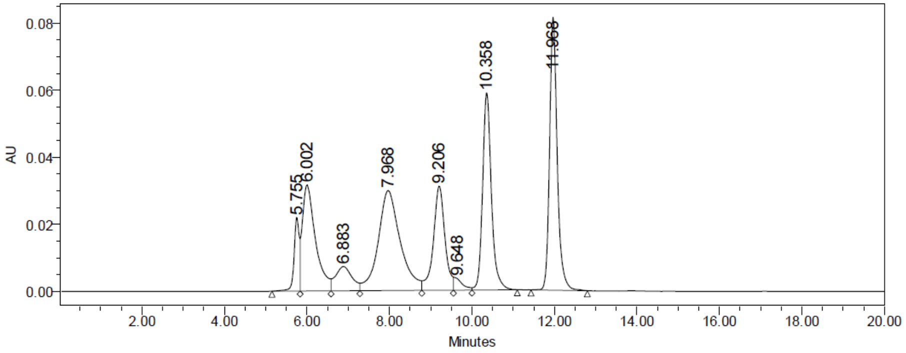

SAMPLE INFORMATION

Sample Name:	382	Acquired By:	harw ilc
Sample Type:	Unknow n	Date Acquired:	8/26/2008 5:06:27 PM
Vial:	1:A,4	Acq. Method Set:	GPC3000 LCH Dual Wavelength
Injection #:	1	Date Processed:	8/28/2008 11:39:07 AM
Injection Volume:	10.60 ul	Processing Method:	ISOBM hCG Samples 082708
Run Time:	20.0 Minutes	Channel Name:	2487Channel 1
Sample Set Name:	GPC 082608 LCH Final	Proc. Chnl. Descr.:	280 nm

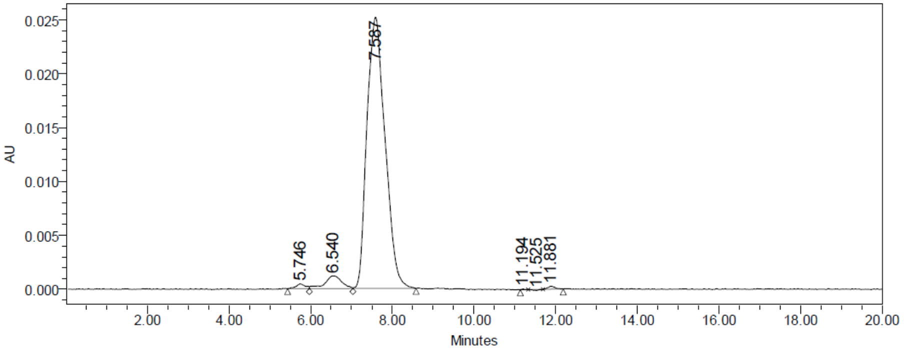

SAMPLE INFORMATION

Sample Name:	383	Acquired By:	harw ilc
Sample Type:	Unknow n	Date Acquired:	8/26/2008 6:09:12 PM
Vial:	1:A,5	Acq. Method Set:	GPC3000 LCH Dual Wavelength
Injection #:	1	Date Processed:	8/28/2008 11:39:07 AM
Injection Volume:	10.90 ul	Processing Method:	ISOBM hCG Samples 082708
Run Time:	20.0 Minutes	Channel Name:	2487Channel 1
Sample Set Name:	GPC 082608 LCH Final	Proc. Chnl. Descr.:	280 nm

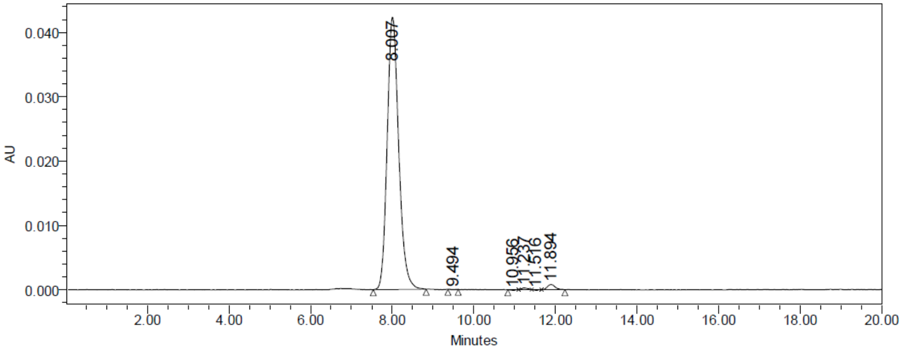

SAMPLE INFORMATION

Sample Name:	384	Acquired By:	harw ilc
Sample Type:	Unknow n	Date Acquired:	8/26/2008 7:11:57 PM
Vial:	1:A,6	Acq. Method Set:	GPC3000 LCH Dual Wavelength
Injection #:	1	Date Processed:	8/28/2008 11:39:07 AM
Injection Volume:	10.10 ul	Processing Method:	ISOBM hCG Samples 082708
Run Time:	20.0 Minutes	Channel Name:	2487Channel 1
Sample Set Name:	GPC 082608 LCH Final	Proc. Chnl. Descr.:	280 nm

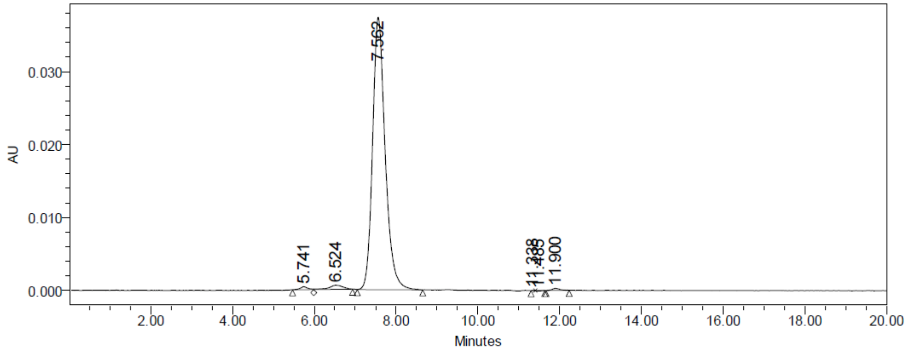

SAMPLE INFORMATION

Sample Name:	385	Acquired By:	harw ilc
Sample Type:	Unknow n	Date Acquired:	8/26/2008 8:14:47 PM
Vial:	1:A,7	Acq. Method Set:	GPC3000 LCH Dual Wavelength
Injection #:	1	Date Processed:	8/28/2008 11:39:07 AM
Injection Volume:	10.30 ul	Processing Method:	ISOBM hCG Samples 082708
Run Time:	20.0 Minutes	Channel Name:	2487Channel 1
Sample Set Name:	GPC 082608 LCH Final	Proc. Chnl. Descr.:	280 nm

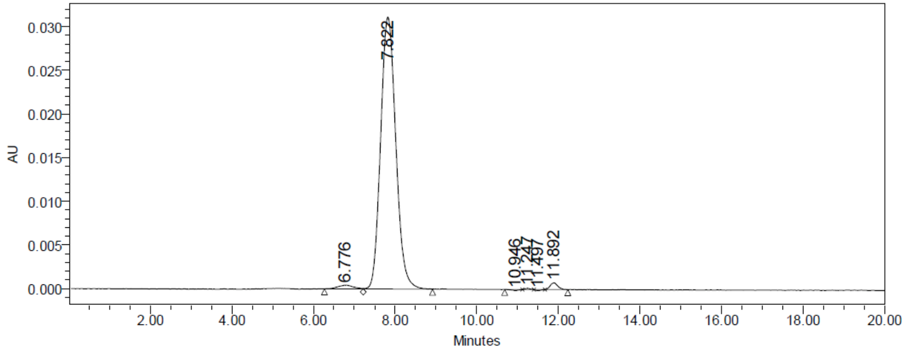

SAMPLE INFORMATION

Sample Name:	386	Acquired By:	harw ilc
Sample Type:	Unknow n	Date Acquired:	8/26/2008 9:17:34 PM
Vial:	1:A,8	Acq. Method Set:	GPC3000 LCH Dual Wavelength
Injection #:	1	Date Processed:	8/28/2008 11:39:07 AM
Injection Volume:	10.80 ul	Processing Method:	ISOBM hCG Samples 082708
Run Time:	20.0 Minutes	Channel Name:	2487Channel 1
Sample Set Name:	GPC 082608 LCH Final	Proc. Chnl. Descr.:	280 nm

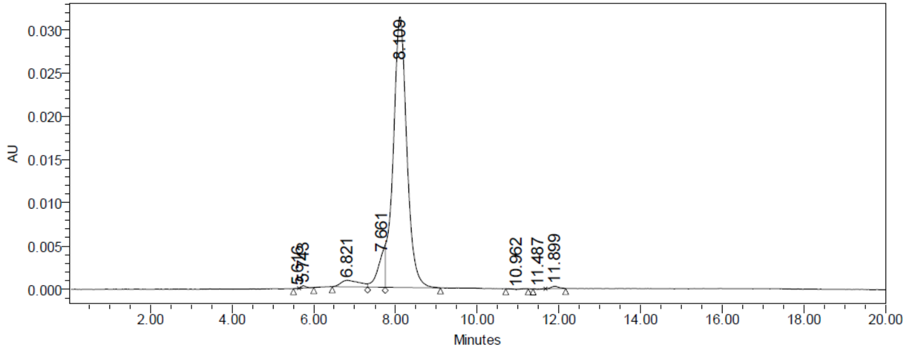

SAMPLE INFORMATION

Sample Name:	387	Acquired By:	harw ilc
Sample Type:	Unknow n	Date Acquired:	8/26/2008 10:20:20 PM
Vial:	1:B,1	Acq. Method Set:	GPC3000 LCH Dual Wavelength
Injection #:	1	Date Processed:	8/28/2008 11:39:07 AM
Injection Volume:	10.00 ul	Processing Method:	ISOBM hCG Samples 082708
Run Time:	20.0 Minutes	Channel Name:	2487Channel 1
Sample Set Name:	GPC 082608 LCH Final	Proc. Chnl. Descr.:	280 nm

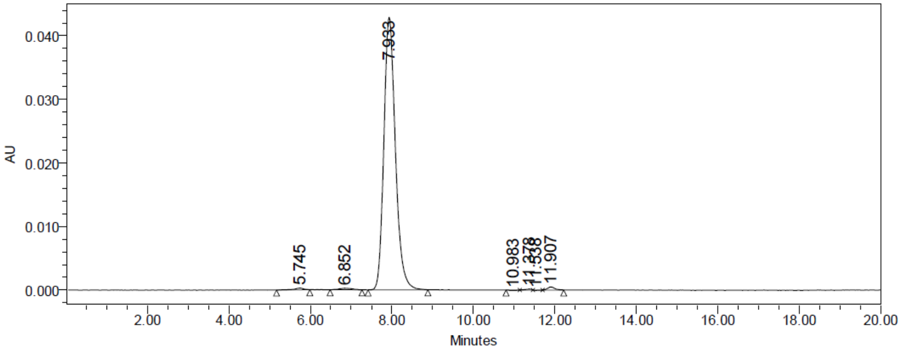

SAMPLE INFORMATION

Sample Name:	388	Acquired By:	harw ilc
Sample Type:	Unknow n	Date Acquired:	8/26/2008 11:23:00 PM
Vial:	1:B,2	Acq. Method Set:	GPC3000 LCH Dual Wavelength
Injection #:	1	Date Processed:	8/28/2008 11:39:08 AM
Injection Volume:	10.30 ul	Processing Method:	ISOBM hCG Samples 082708
Run Time:	20.0 Minutes	Channel Name:	2487Channel 1
Sample Set Name:	GPC 082608 LCH Final	Proc. Chnl. Descr.:	280 nm

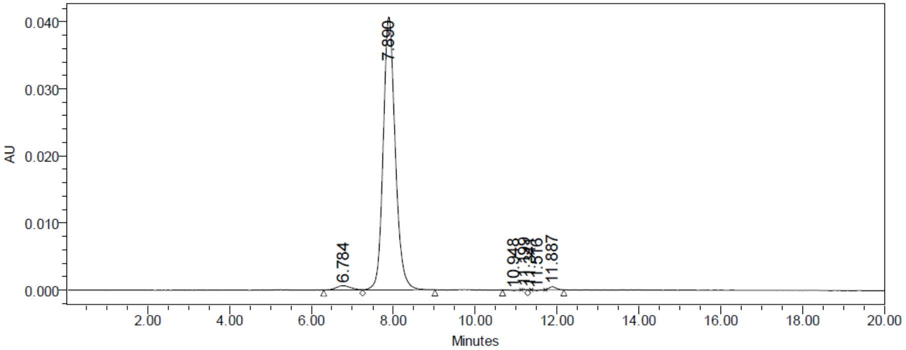

SAMPLE INFORMATION

Sample Name:	389	Acquired By:	harw ilc
Sample Type:	Unknow n	Date Acquired:	8/27/2008 12:25:44 AM
Vial:	1:B,3	Acq. Method Set:	GPC3000 LCH Dual Wavelength
Injection #:	1	Date Processed:	8/28/2008 11:39:08 AM
Injection Volume:	10.10 ul	Processing Method:	ISOBM hCG Samples 082708
Run Time:	20.0 Minutes	Channel Name:	2487Channel 1
Sample Set Name:	GPC 082608 LCH Final	Proc. Chnl. Descr.:	280 nm

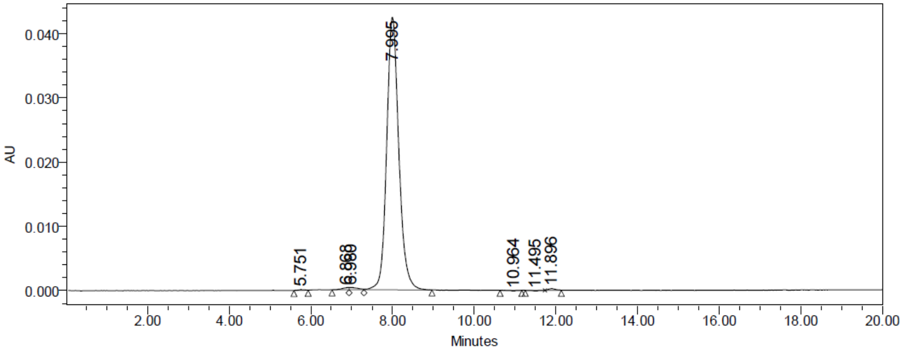

SAMPLE INFORMATION

Sample Name:	390	Acquired By:	harw ilc
Sample Type:	Unknow n	Date Acquired:	8/27/2008 1:28:26 AM
Vial:	1:B,4	Acq. Method Set:	GPC3000 LCH Dual Wavelength
Injection #:	1	Date Processed:	8/28/2008 11:39:08 AM
Injection Volume:	11.90 ul	Processing Method:	ISOBM hCG Samples 082708
Run Time:	20.0 Minutes	Channel Name:	2487Channel 1
Sample Set Name:	GPC 082608 LCH Final	Proc. Chnl. Descr.:	280 nm

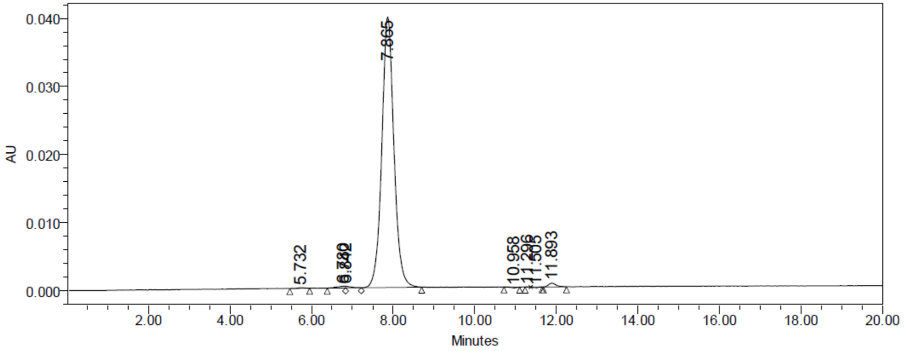

SAMPLE INFORMATION

Sample Name:	391	Acquired By:	harw ilc
Sample Type:	Unknow n	Date Acquired:	8/27/2008 2:31:10 AM
Vial:	1:B,5	Acq. Method Set:	GPC3000 LCH Dual Wavelength
Injection #:	1	Date Processed:	8/28/2008 11:39:08 AM
Injection Volume:	10.10 ul	Processing Method:	ISOBM hCG Samples 082708
Run Time:	20.0 Minutes	Channel Name:	2487Channel 1
Sample Set Name:	GPC 082608 LCH Final	Proc. Chnl. Descr.:	280 nm

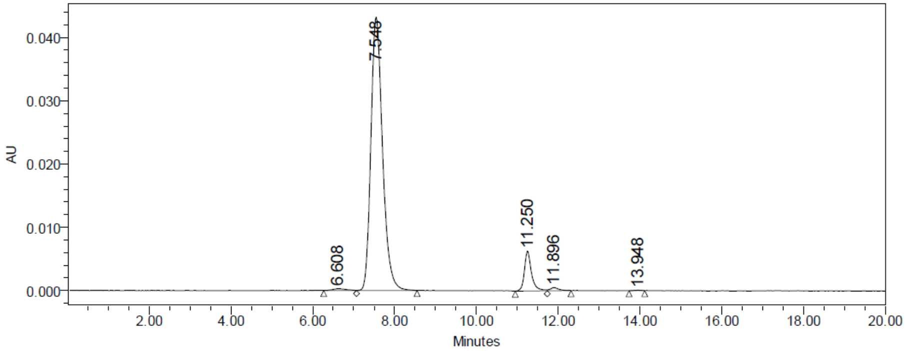

SAMPLE INFORMATION

Sample Name:	392	Acquired By:	harwilc
Sample Type:	Unknown	Date Acquired:	8/27/2008 3:33:55 AM
Vial:	1:B,6	Acq. Method Set:	GPC3000 LCH Dual Wavelength
Injection #:	1	Date Processed:	8/28/2008 11:39:08 AM
Injection Volume:	12.80 ul	Processing Method:	ISOBM hCG Samples 082708
Run Time:	20.0 Minutes	Channel Name:	2487Channel 1
Sample Set Name:	GPC 082608 LCH Final	Proc. Chnl. Descr.:	280 nm

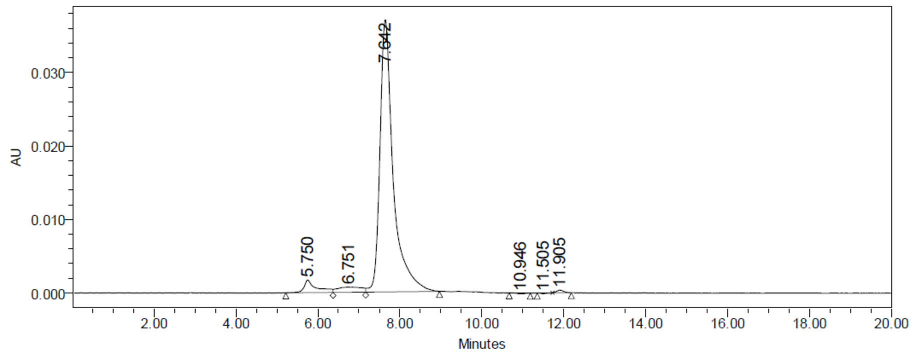

SAMPLE INFORMATION

Sample Name:	393	Acquired By:	harwilc
Sample Type:	Unknown	Date Acquired:	8/29/2008 4:32:21 PM
Vial:	2:D,1	Acq. Method Set:	GPC3000 LCH Dual Wavelength
Injection #:	1	Date Processed:	9/2/2008 10:17:52 AM
Injection Volume:	10.80 ul	Processing Method:	ISOBM hCG Samples 082708
Run Time:	20.0 Minutes	Channel Name:	2487Channel 1
Sample Set Name:	GPC 082608 LCH Final	Proc. Chnl. Descr.:	280 nm

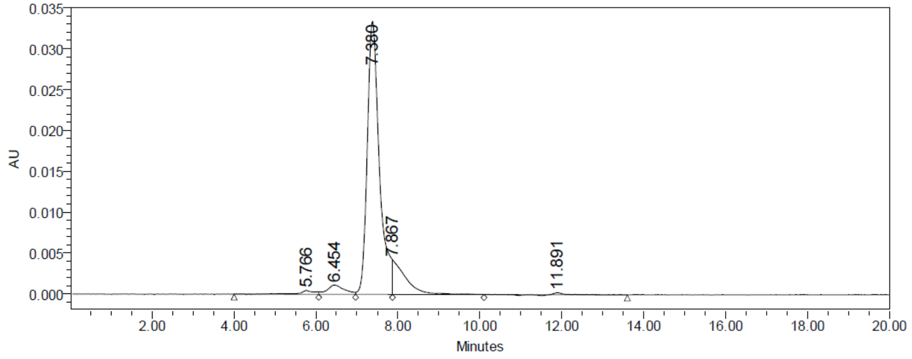

SAMPLE INFORMATION

Sample Name:	394	Acquired By:	harwilc
Sample Type:	Unknown	Date Acquired:	8/27/2008 4:57:43 AM
Vial:	1:B,8	Acq. Method Set:	GPC3000 LCH Dual Wavelength
Injection #:	1	Date Processed:	8/28/2008 11:39:08 AM
Injection Volume:	10.00 ul	Processing Method:	ISOBM hCG Samples 082708
Run Time:	20.0 Minutes	Channel Name:	2487Channel 1
Sample Set Name:	GPC 082608 LCH Final	Proc. Chnl. Descr.:	280 nm

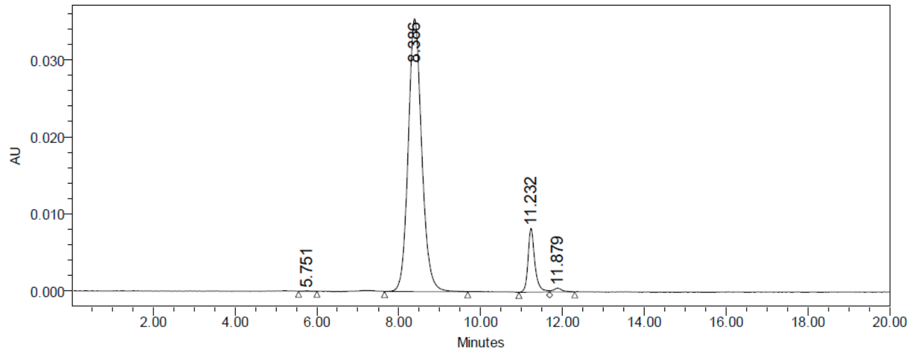

SAMPLE INFORMATION

Sample Name:	395	Acquired By:	harwilc
Sample Type:	Unknown	Date Acquired:	8/27/2008 6:00:26 AM
Vial:	1:C,1	Acq. Method Set:	GPC3000 LCH Dual Wavelength
Injection #:	1	Date Processed:	8/28/2008 1:14:15 PM
Injection Volume:	10.00 ul	Processing Method:	ISOBM hCG Samples 082708
Run Time:	20.0 Minutes	Channel Name:	2487Channel 1
Sample Set Name:	GPC 082608 LCH Final	Proc. Chnl. Descr.:	280 nm

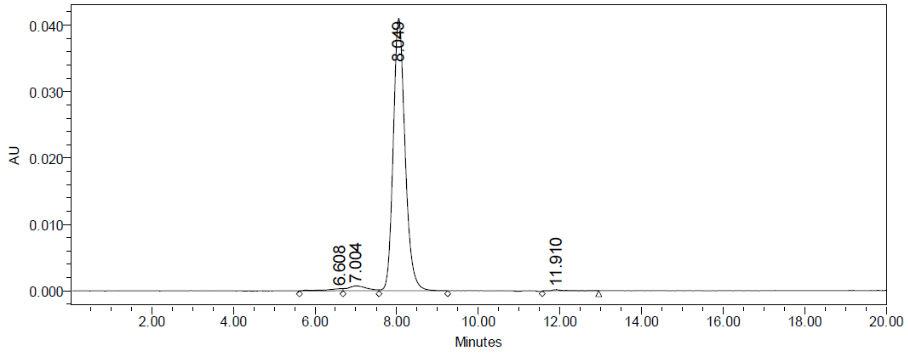

SAMPLE INFORMATION

Sample Name:	396	Acquired By:	harw ilc
Sample Type:	Unknow n	Date Acquired:	8/27/2008 7:03:06 AM
Vial:	1:C,2	Acq. Method Set:	GPC3000 LCH Dual Wavelength
Injection #:	1	Date Processed:	8/28/2008 11:39:09 AM
Injection Volume:	10.30 ul	Processing Method:	ISOBM hCG Samples 082708
Run Time:	20.0 Minutes	Channel Name:	2487Channel 1
Sample Set Name:	GPC 082608 LCH Final	Proc. Chnl. Descr.:	280 nm

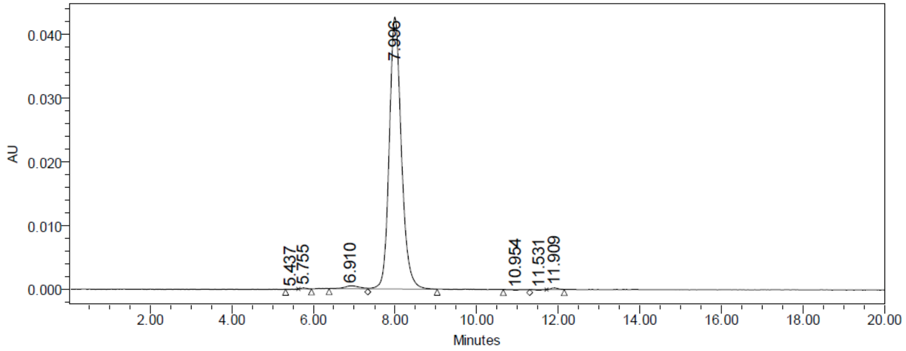

SAMPLE INFORMATION

Sample Name:	397	Acquired By:	harw ilc
Sample Type:	Unknow n	Date Acquired:	8/27/2008 8:05:45 AM
Vial:	1:C,3	Acq. Method Set:	GPC3000 LCH Dual Wavelength
Injection #:	1	Date Processed:	8/28/2008 11:39:09 AM
Injection Volume:	10.80 ul	Processing Method:	ISOBM hCG Samples 082708
Run Time:	20.0 Minutes	Channel Name:	2487Channel 1
Sample Set Name:	GPC 082608 LCH Final	Proc. Chnl. Descr.:	280 nm

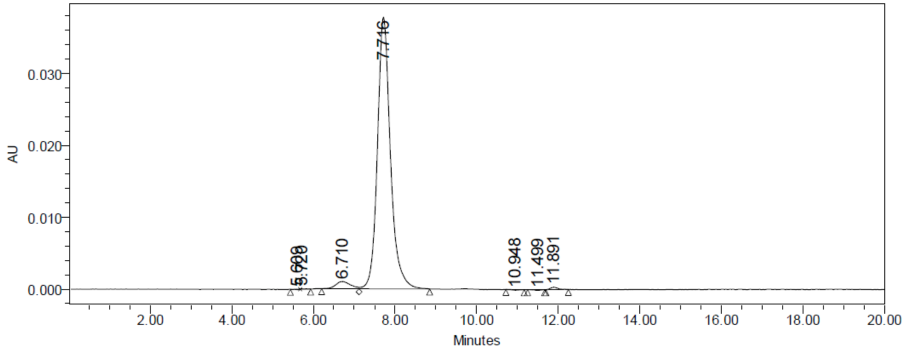

SAMPLE INFORMATION

Sample Name:	398	Acquired By:	harw ilc
Sample Type:	Unknow n	Date Acquired:	8/27/2008 9:08:26 AM
Vial:	1:C,4	Acq. Method Set:	GPC3000 LCH Dual Wavelength
Injection #:	1	Date Processed:	8/28/2008 11:39:09 AM
Injection Volume:	10.80 ul	Processing Method:	ISOBM hCG Samples 082708
Run Time:	20.0 Minutes	Channel Name:	2487Channel 1
Sample Set Name:	GPC 082608 LCH Final	Proc. Chnl. Descr.:	280 nm

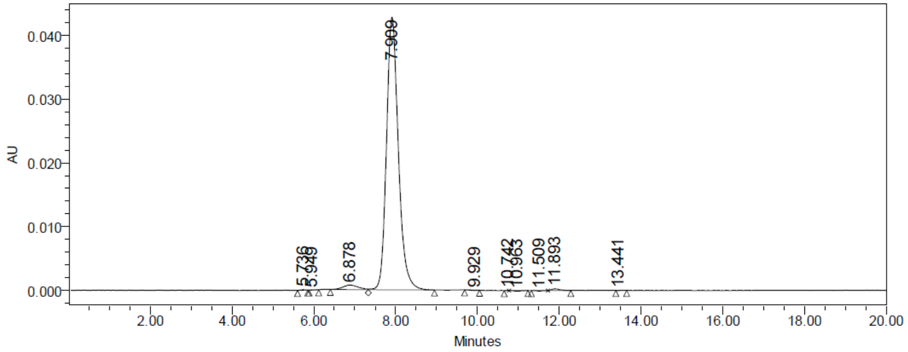

SAMPLE INFORMATION

Sample Name:	399	Acquired By:	harw ilc
Sample Type:	Unknow n	Date Acquired:	8/27/2008 10:11:11 AM
Vial:	1:C,5	Acq. Method Set:	GPC3000 LCH Dual Wavelength
Injection #:	1	Date Processed:	8/28/2008 11:39:09 AM
Injection Volume:	10.00 ul	Processing Method:	ISOBM hCG Samples 082708
Run Time:	20.0 Minutes	Channel Name:	2487Channel 1
Sample Set Name:	GPC 082608 LCH Final	Proc. Chnl. Descr.:	280 nm

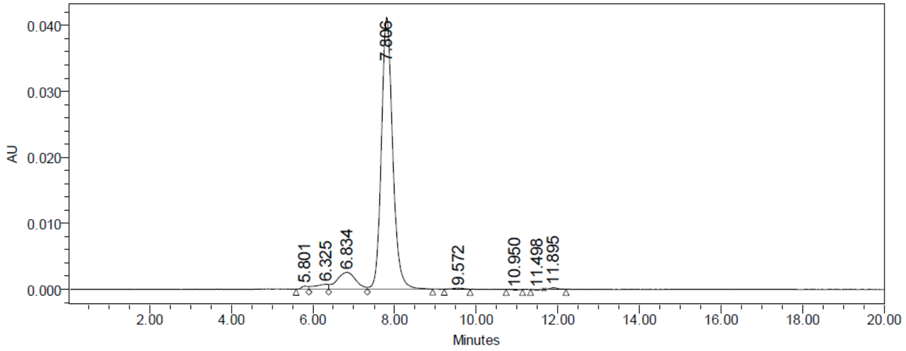

SAMPLE INFORMATION

Sample Name:	400	Acquired By:	harwilc
Sample Type:	Unknown	Date Acquired:	8/27/2008 11:13:57 AM
Vial:	1:C,6	Acq. Method Set:	GPC3000 LCH Dual Wavelength
Injection #:	1	Date Processed:	8/28/2008 11:39:09 AM
Injection Volume:	10.00 ul	Processing Method:	ISOBM hCG Samples 082708
Run Time:	20.0 Minutes	Channel Name:	2487Channel 1
Sample Set Name:	GPC 082608 LCH Final	Proc. Chnl. Descr.:	280 nm

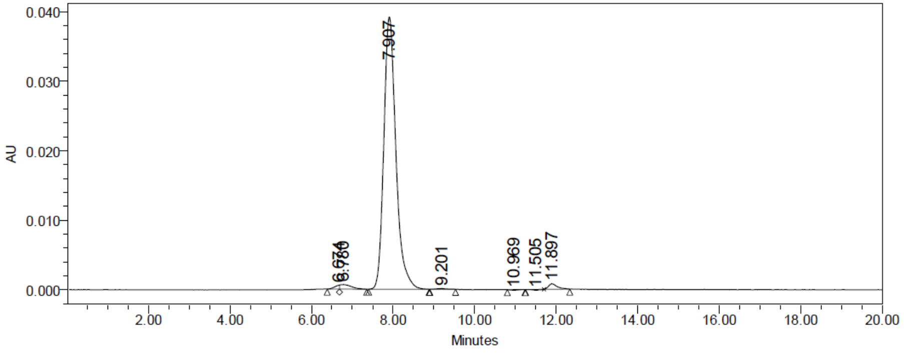

SAMPLE INFORMATION

Sample Name:	401	Acquired By:	harwilc
Sample Type:	Unknown	Date Acquired:	8/27/2008 12:16:41 PM
Vial:	1:C,7	Acq. Method Set:	GPC3000 LCH Dual Wavelength
Injection #:	1	Date Processed:	8/28/2008 11:39:09 AM
Injection Volume:	11.10 ul	Processing Method:	ISOBM hCG Samples 082708
Run Time:	20.0 Minutes	Channel Name:	2487Channel 1
Sample Set Name:	GPC 082608 LCH Final	Proc. Chnl. Descr.:	280 nm

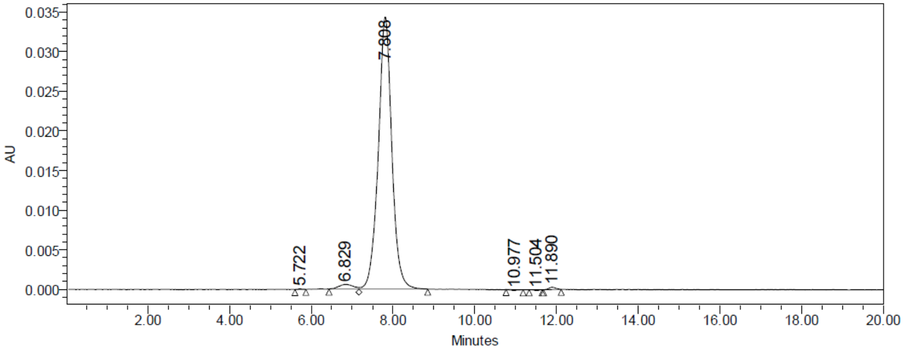

SAMPLE INFORMATION

Sample Name:	402	Acquired By:	harwilc
Sample Type:	Unknown	Date Acquired:	8/27/2008 1:19:26 PM
Vial:	1:C,8	Acq. Method Set:	GPC3000 LCH Dual Wavelength
Injection #:	1	Date Processed:	8/28/2008 11:39:10 AM
Injection Volume:	10.30 ul	Processing Method:	ISOBM hCG Samples 082708
Run Time:	20.0 Minutes	Channel Name:	2487Channel 1
Sample Set Name:	GPC 082608 LCH Final	Proc. Chnl. Descr.:	280 nm

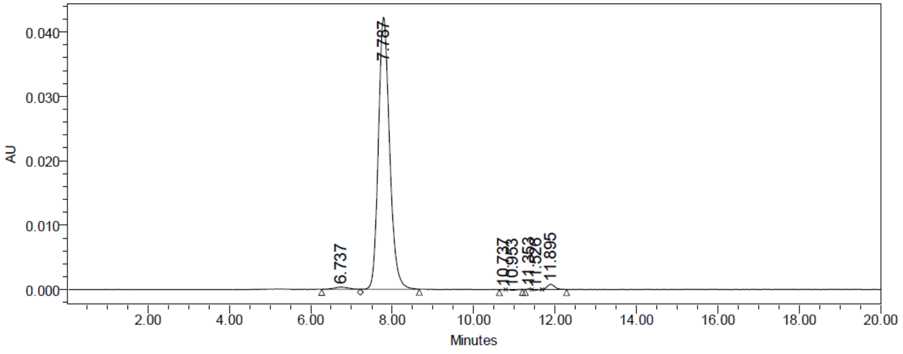

SAMPLE INFORMATION

Sample Name:	403	Acquired By:	harwilc
Sample Type:	Unknown	Date Acquired:	8/27/2008 2:22:10 PM
Vial:	1:D,1	Acq. Method Set:	GPC3000 LCH Dual Wavelength
Injection #:	1	Date Processed:	8/28/2008 11:39:10 AM
Injection Volume:	10.10 ul	Processing Method:	ISOBM hCG Samples 082708
Run Time:	20.0 Minutes	Channel Name:	2487Channel 1
Sample Set Name:	GPC 082608 LCH Final	Proc. Chnl. Descr.:	280 nm

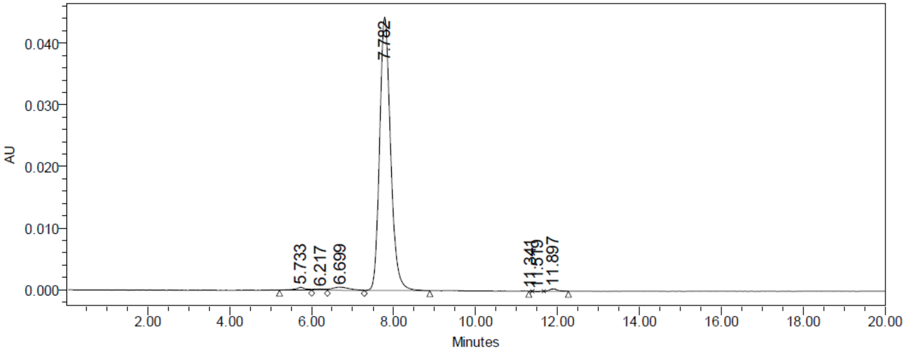

SAMPLE INFORMATION

Sample Name:	404	Acquired By:	harwilc
Sample Type:	Unknown	Date Acquired:	8/27/2008 3:24:46 PM
Vial:	1:D,2	Acq. Method Set:	GPC3000 LCH Dual Wavelength
Injection #:	1	Date Processed:	8/28/2008 11:39:10 AM
Injection Volume:	10.10 ul	Processing Method:	ISOBM hCG Samples 082708
Run Time:	20.0 Minutes	Channel Name:	2487Channel 1
Sample Set Name:	GPC 082608 LCH Final	Proc. Chnl. Descr.:	280 nm

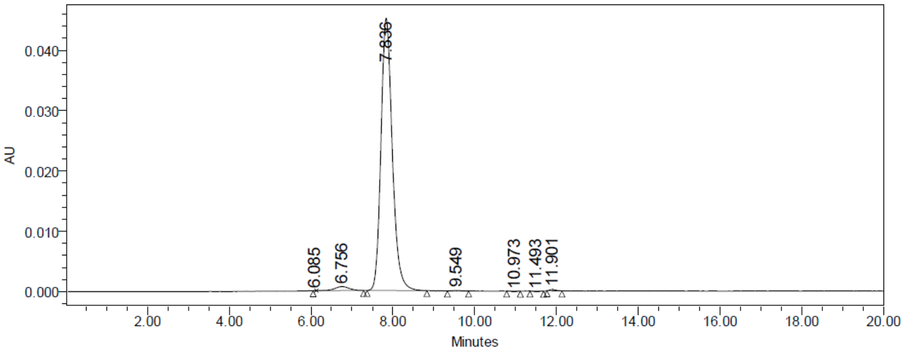

SAMPLE INFORMATION

Sample Name:	405	Acquired By:	harwilc
Sample Type:	Unknown	Date Acquired:	8/27/2008 4:27:25 PM
Vial:	1:D,3	Acq. Method Set:	GPC3000 LCH Dual Wavelength
Injection #:	1	Date Processed:	8/28/2008 11:39:10 AM
Injection Volume:	11.10 ul	Processing Method:	ISOBM hCG Samples 082708
Run Time:	20.0 Minutes	Channel Name:	2487Channel 1
Sample Set Name:	GPC 082608 LCH Final	Proc. Chnl. Descr.:	280 nm

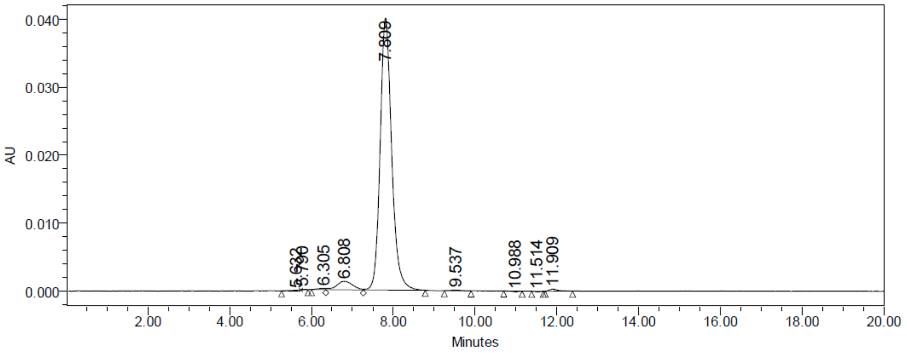

SAMPLE INFORMATION

Sample Name:	406	Acquired By:	harwilc
Sample Type:	Unknown	Date Acquired:	8/27/2008 5:50:57 PM
Vial:	1:D,4	Acq. Method Set:	GPC3000 LCH Dual Wavelength
Injection #:	2	Date Processed:	8/28/2008 11:39:11 AM
Injection Volume:	10.80 ul	Processing Method:	ISOBM hCG Samples 082708
Run Time:	20.0 Minutes	Channel Name:	2487Channel 1
Sample Set Name:	GPC 082608 LCH Final	Proc. Chnl. Descr.:	280 nm

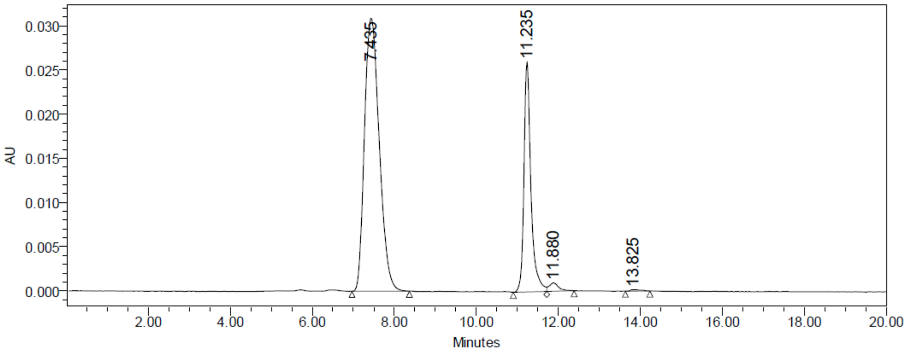

SAMPLE INFORMATION

Sample Name:	407	Acquired By:	harwilc
Sample Type:	Unknown	Date Acquired:	8/27/2008 6:32:46 PM
Vial:	1:D,5	Acq. Method Set:	GPC3000 LCH Dual Wavelength
Injection #:	1	Date Processed:	8/28/2008 11:39:12 AM
Injection Volume:	10.00 ul	Processing Method:	ISOBM hCG Samples 082708
Run Time:	20.0 Minutes	Channel Name:	2487Channel 1
Sample Set Name:	GPC 082608 LCH Final	Proc. Chnl. Descr.:	280 nm

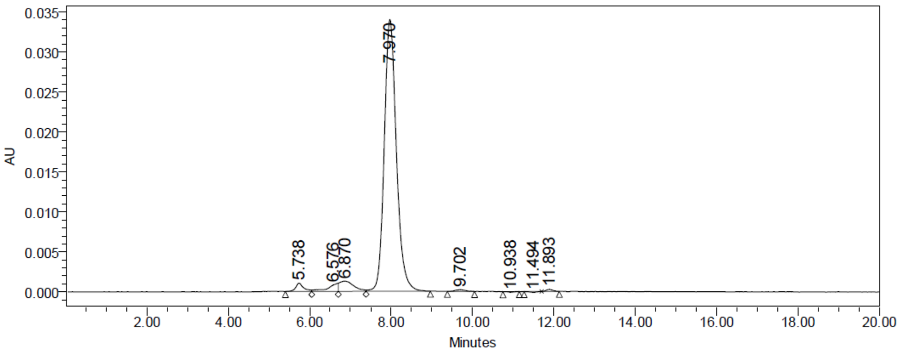

SAMPLE INFORMATION

Sample Name:	408	Acquired By:	harwilc
Sample Type:	Unknown	Date Acquired:	8/27/2008 7:35:27 PM
Vial:	1:D,6	Acq. Method Set:	GPC3000 LCH Dual Wavelength
Injection #:	1	Date Processed:	8/28/2008 11:39:12 AM
Injection Volume:	10.00 ul	Processing Method:	ISOBM hCG Samples 082708
Run Time:	20.0 Minutes	Channel Name:	2487Channel 1
Sample Set Name:	GPC 082608 LCH Final	Proc. Chnl. Descr.:	280 nm

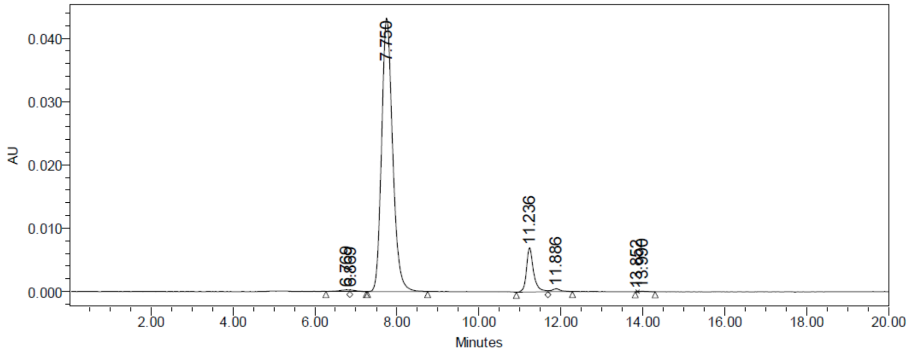

SAMPLE INFORMATION

Sample Name:	409	Acquired By:	harwilc
Sample Type:	Unknown	Date Acquired:	8/27/2008 8:38:12 PM
Vial:	1:D,7	Acq. Method Set:	GPC3000 LCH Dual Wavelength
Injection #:	1	Date Processed:	8/28/2008 11:39:12 AM
Injection Volume:	10.90 ul	Processing Method:	ISOBM hCG Samples 082708
Run Time:	20.0 Minutes	Channel Name:	2487Channel 1
Sample Set Name:	GPC 082608 LCH Final	Proc. Chnl. Descr.:	280 nm

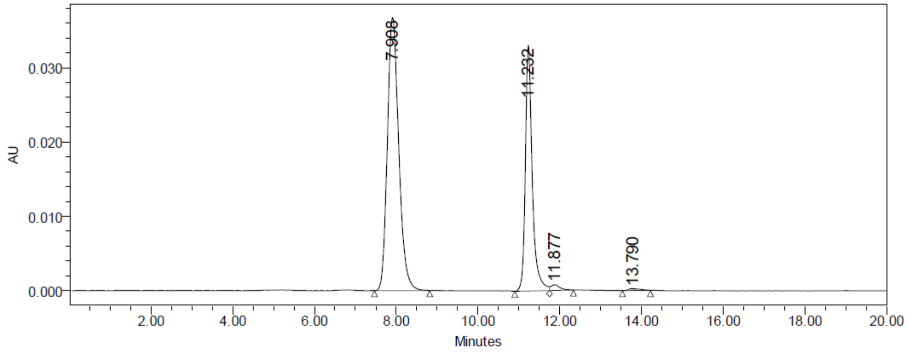

SAMPLE INFORMATION

Sample Name:	410	Acquired By:	harwilc
Sample Type:	Unknown	Date Acquired:	8/27/2008 9:40:57 PM
Vial:	1:D,8	Acq. Method Set:	GPC3000 LCH Dual Wavelength
Injection #:	1	Date Processed:	8/28/2008 11:39:12 AM
Injection Volume:	10.00 ul	Processing Method:	ISOBM hCG Samples 082708
Run Time:	20.0 Minutes	Channel Name:	2487Channel 1
Sample Set Name:	GPC 082608 LCH Final	Proc. Chnl. Descr.:	280 nm

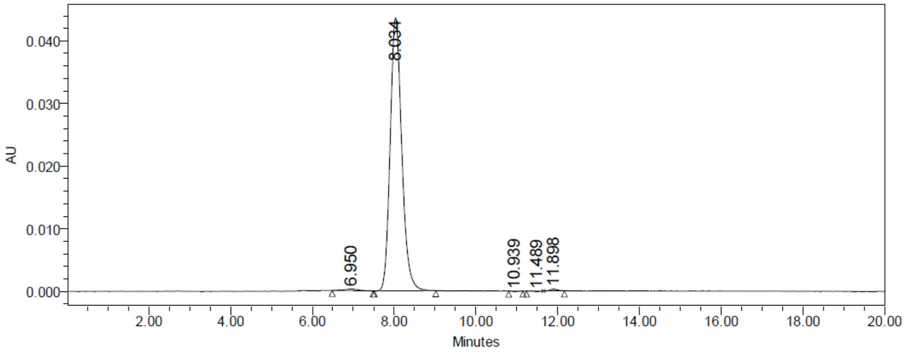

SAMPLE INFORMATION

Sample Name:	411	Acquired By:	harwilc
Sample Type:	Unknown	Date Acquired:	8/27/2008 10:43:40 PM
Vial:	1:E,1	Acq. Method Set:	GPC3000 LCH Dual Wavelength
Injection #:	1	Date Processed:	8/28/2008 3:37:42 PM
Injection Volume:	10.00 ul	Processing Method:	ISOBM hCG Samples 082708
Run Time:	20.0 Minutes	Channel Name:	2487Channel 1
Sample Set Name:	GPC 082608 LCH Final	Proc. Chnl. Descr.:	280 nm

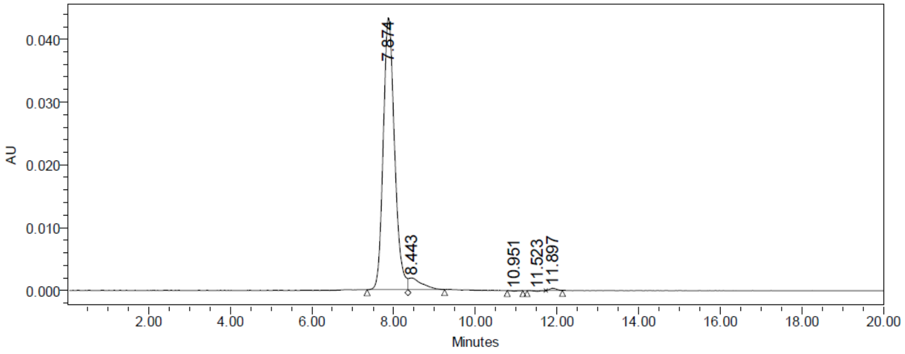

SAMPLE INFORMATION

Sample Name:	412	Acquired By:	harwilc
Sample Type:	Unknown	Date Acquired:	8/27/2008 11:46:16 PM
Vial:	1:E,2	Acq. Method Set:	GPC3000 LCH Dual Wavelength
Injection #:	1	Date Processed:	8/28/2008 3:37:43 PM
Injection Volume:	13.50 ul	Processing Method:	ISOBM hCG Samples 082708
Run Time:	20.0 Minutes	Channel Name:	2487Channel 1
Sample Set Name:	GPC 082608 LCH Final	Proc. Chnl. Descr.:	280 nm

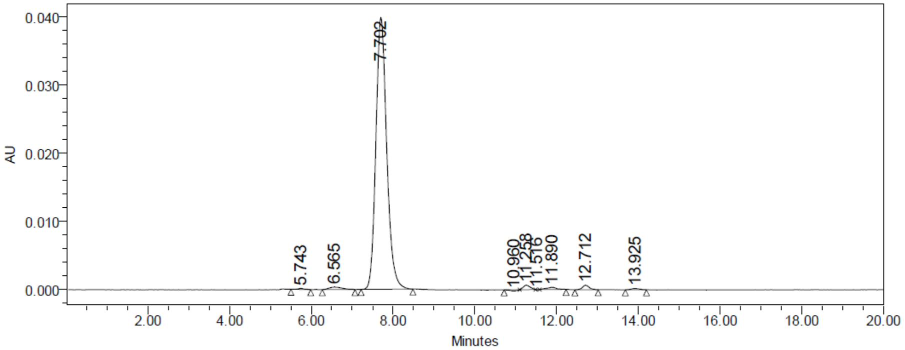

SAMPLE INFORMATION

Sample Name:	413	Acquired By:	harwilc
Sample Type:	Unknown	Date Acquired:	8/28/2008 12:48:59 AM
Vial:	1:E,3	Acq. Method Set:	GPC3000 LCH Dual Wavelength
Injection #:	1	Date Processed:	8/28/2008 3:37:43 PM
Injection Volume:	10.00 ul	Processing Method:	ISOBM hCG Samples 082708
Run Time:	20.0 Minutes	Channel Name:	2487Channel 1
Sample Set Name:	GPC 082608 LCH Final	Proc. Chnl. Descr.:	280 nm

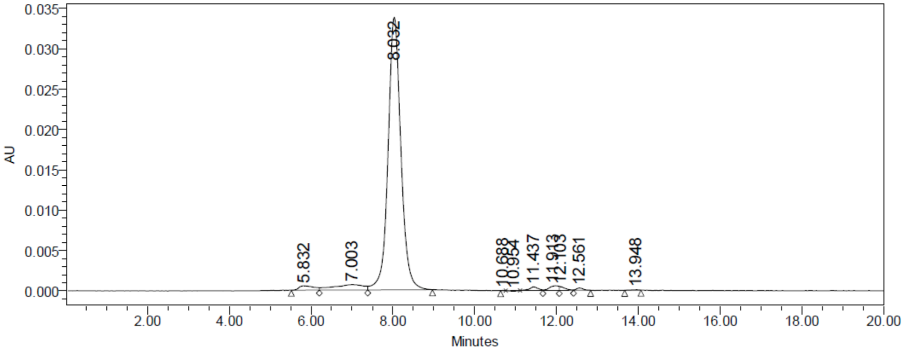

SAMPLE INFORMATION

Sample Name:	414	Acquired By:	harwilc
Sample Type:	Unknown	Date Acquired:	8/28/2008 1:51:39 AM
Vial:	1:E,4	Acq. Method Set:	GPC3000 LCH Dual Wavelength
Injection #:	1	Date Processed:	8/28/2008 3:37:43 PM
Injection Volume:	10.40 ul	Processing Method:	ISOBM hCG Samples 082708
Run Time:	20.0 Minutes	Channel Name:	2487Channel 1
Sample Set Name:	GPC 082608 LCH Final	Proc. Chnl. Descr.:	280 nm

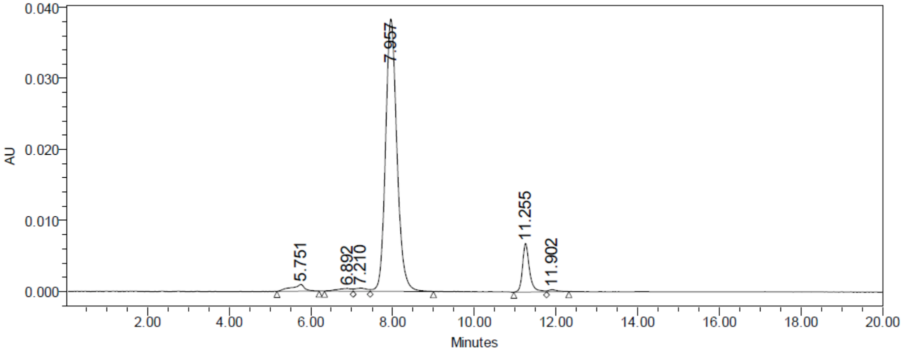

SAMPLE INFORMATION

Sample Name:	415	Acquired By:	harwilc
Sample Type:	Unknown	Date Acquired:	8/28/2008 2:54:17 AM
Vial:	1:E,5	Acq. Method Set:	GPC3000 LCH Dual Wavelength
Injection #:	1	Date Processed:	8/28/2008 3:37:44 PM
Injection Volume:	10.00 ul	Processing Method:	ISOBM hCG Samples 082708
Run Time:	20.0 Minutes	Channel Name:	2487Channel 1
Sample Set Name:	GPC 082608 LCH Final	Proc. Chnl. Descr.:	280 nm

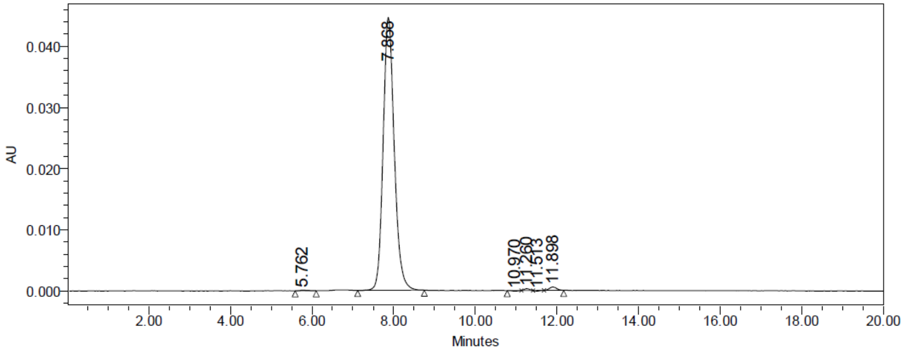

SAMPLE INFORMATION

Sample Name:	416	Acquired By:	harw ilc
Sample Type:	Unknow n	Date Acquired:	8/28/2008 4:17:50 AM
Vial:	1:E,6	Acq. Method Set:	GPC3000 LCH Dual Wavelength
Injection #:	2	Date Processed:	8/28/2008 3:37:44 PM
Injection Volume:	10.00 ul	Processing Method:	ISOBM hCG Samples 082708
Run Time:	20.0 Minutes	Channel Name:	2487Channel 1
Sample Set Name:	GPC 082608 LCH Final	Proc. Chnl. Descr.:	280 nm

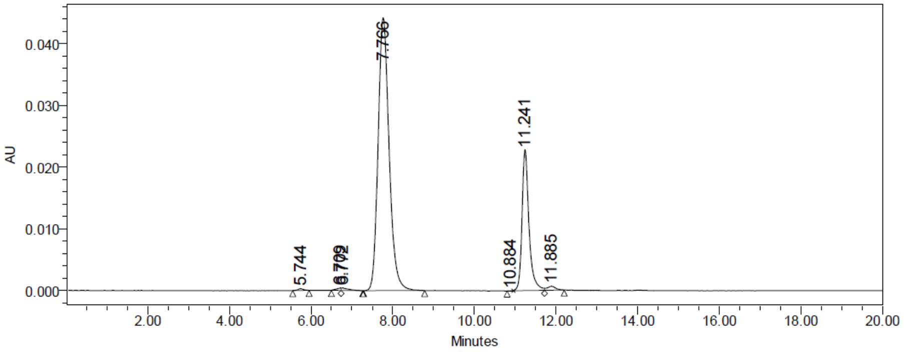

SAMPLE INFORMATION

Sample Name:	417	Acquired By:	harw ilc
Sample Type:	Unknow n	Date Acquired:	8/28/2008 4:59:39 AM
Vial:	1:E,7	Acq. Method Set:	GPC3000 LCH Dual Wavelength
Injection #:	1	Date Processed:	8/28/2008 3:37:44 PM
Injection Volume:	10.10 ul	Processing Method:	ISOBM hCG Samples 082708
Run Time:	20.0 Minutes	Channel Name:	2487Channel 1
Sample Set Name:	GPC 082608 LCH Final	Proc. Chnl. Descr.:	280 nm

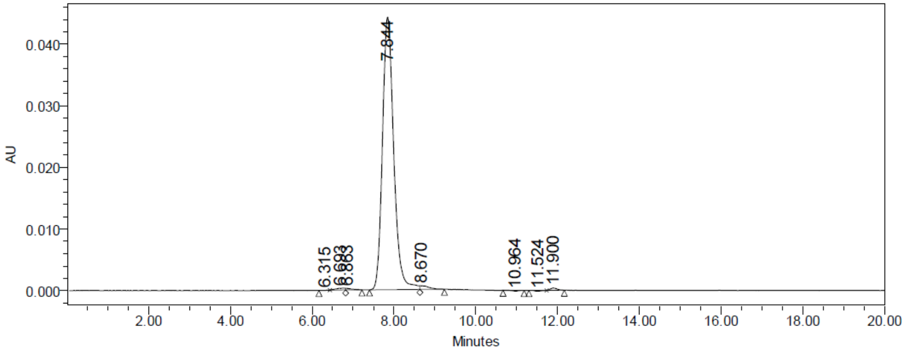

SAMPLE INFORMATION

Sample Name:	418	Acquired By:	harw ilc
Sample Type:	Unknow n	Date Acquired:	8/28/2008 6:02:25 AM
Vial:	1:E,8	Acq. Method Set:	GPC3000 LCH Dual Wavelength
Injection #:	1	Date Processed:	8/28/2008 3:37:44 PM
Injection Volume:	10.00 ul	Processing Method:	ISOBM hCG Samples 082708
Run Time:	20.0 Minutes	Channel Name:	2487Channel 1
Sample Set Name:	GPC 082608 LCH Final	Proc. Chnl. Descr.:	280 nm

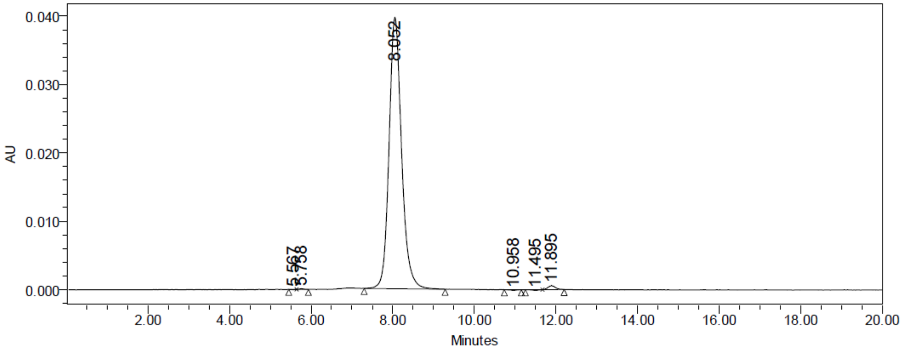

SAMPLE INFORMATION

Sample Name:	419	Acquired By:	harw ilc
Sample Type:	Unknow n	Date Acquired:	8/28/2008 7:05:09 AM
Vial:	1:F,1	Acq. Method Set:	GPC3000 LCH Dual Wavelength
Injection #:	1	Date Processed:	8/28/2008 3:37:44 PM
Injection Volume:	10.00 ul	Processing Method:	ISOBM hCG Samples 082708
Run Time:	20.0 Minutes	Channel Name:	2487Channel 1
Sample Set Name:	GPC 082608 LCH Final	Proc. Chnl. Descr.:	280 nm

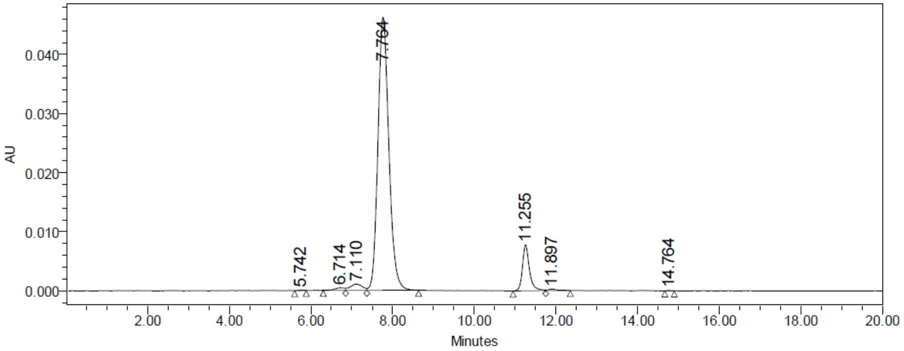

SAMPLE INFORMATION

Sample Name:	420	Acquired By:	harw ilc
Sample Type:	Unknow n	Date Acquired:	8/28/2008 8:07:44 AM
Vial:	1:F,2	Acq. Method Set:	GPC3000 LCH Dual Wavelength
Injection #:	1	Date Processed:	8/28/2008 3:37:44 PM
Injection Volume:	10.90 ul	Processing Method:	ISOBM hCG Samples 082708
Run Time:	20.0 Minutes	Channel Name:	2487Channel 1
Sample Set Name:	GPC 082608 LCH Final	Proc. Chnl. Descr.:	280 nm

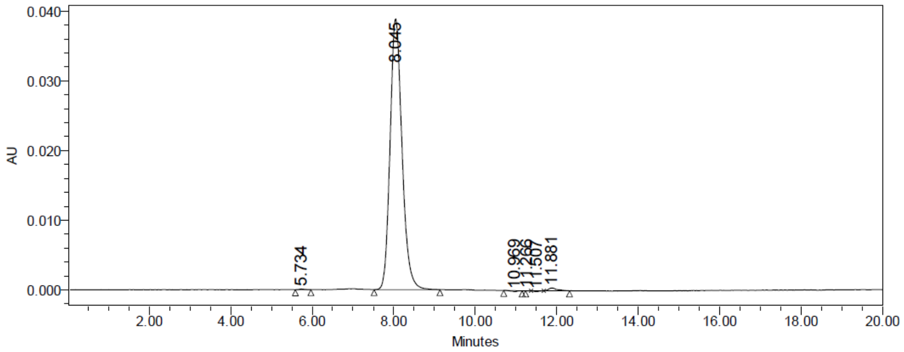

SAMPLE INFORMATION

Sample Name:	421	Acquired By:	harw ilc
Sample Type:	Unknow n	Date Acquired:	8/28/2008 9:10:23 AM
Vial:	1:F,3	Acq. Method Set:	GPC3000 LCH Dual Wavelength
Injection #:	1	Date Processed:	8/28/2008 3:37:44 PM
Injection Volume:	10.90 ul	Processing Method:	ISOBM hCG Samples 082708
Run Time:	20.0 Minutes	Channel Name:	2487Channel 1
Sample Set Name:	GPC 082608 LCH Final	Proc. Chnl. Descr.:	280 nm

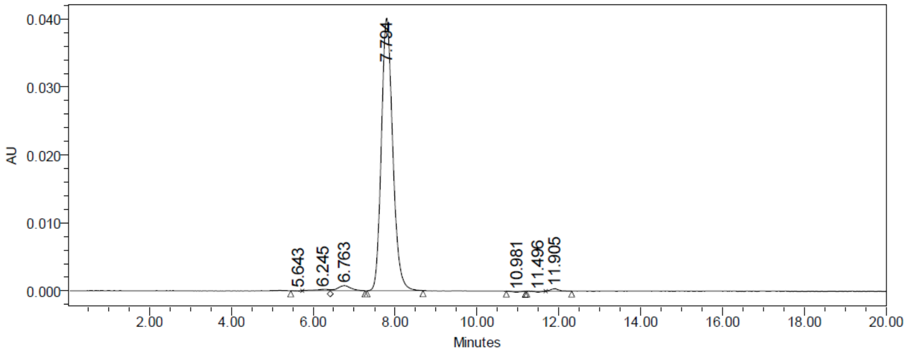

SAMPLE INFORMATION

Sample Name:	422	Acquired By:	harw ilc
Sample Type:	Unknow n	Date Acquired:	8/28/2008 10:13:00 AM
Vial:	1:F,4	Acq. Method Set:	GPC3000 LCH Dual Wavelength
Injection #:	1	Date Processed:	8/28/2008 3:37:45 PM
Injection Volume:	10.00 ul	Processing Method:	ISOBM hCG Samples 082708
Run Time:	20.0 Minutes	Channel Name:	2487Channel 1
Sample Set Name:	GPC 082608 LCH Final	Proc. Chnl. Descr.:	280 nm

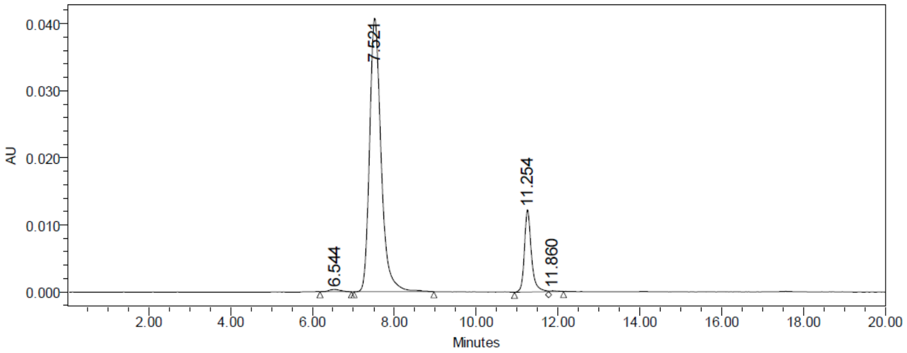

SAMPLE INFORMATION

Sample Name:	423	Acquired By:	harw ilc
Sample Type:	Unknow n	Date Acquired:	8/28/2008 11:15:39 AM
Vial:	1:F,5	Acq. Method Set:	GPC3000 LCH Dual Wavelength
Injection #:	1	Date Processed:	8/28/2008 3:37:45 PM
Injection Volume:	10.00 ul	Processing Method:	ISOBM hCG Samples 082708
Run Time:	20.0 Minutes	Channel Name:	2487Channel 1
Sample Set Name:	GPC 082608 LCH Final	Proc. Chnl. Descr.:	280 nm

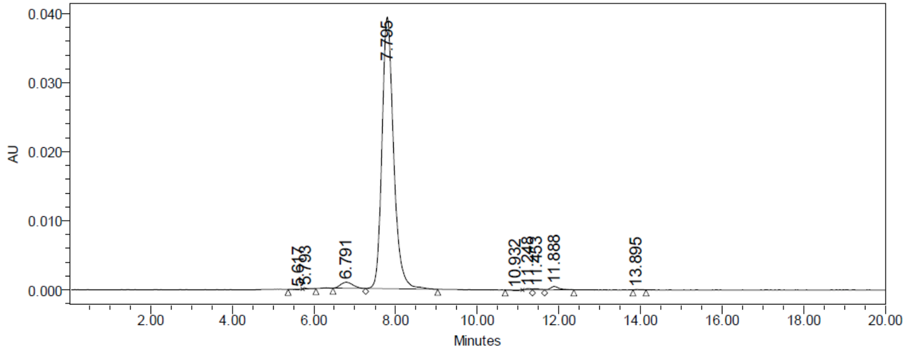

SAMPLE INFORMATION

Sample Name:	424	Acquired By:	harw ilc
Sample Type:	Unknow n	Date Acquired:	8/28/2008 12:18:18 PM
Vial:	1:F,6	Acq. Method Set:	GPC3000 LCH Dual Wavelength
Injection #:	1	Date Processed:	8/28/2008 3:37:45 PM
Injection Volume:	10.00 ul	Processing Method:	ISOBM hCG Samples 082708
Run Time:	20.0 Minutes	Channel Name:	2487Channel 1
Sample Set Name:	GPC 082608 LCH Final	Proc. Chnl. Descr.:	280 nm

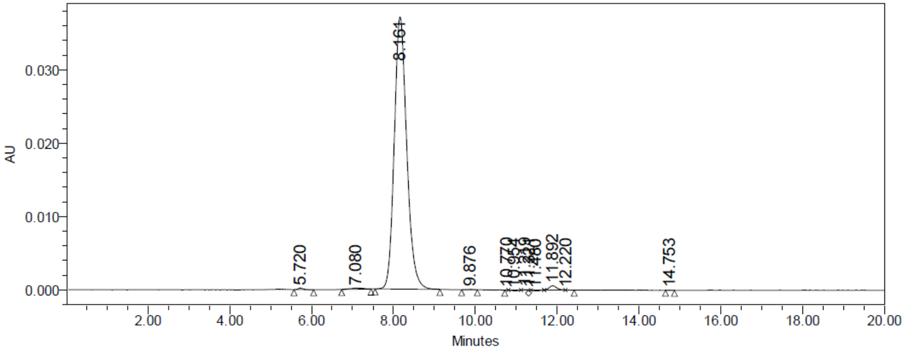

SAMPLE INFORMATION

Sample Name:	425	Acquired By:	harw ilc
Sample Type:	Unknow n	Date Acquired:	8/28/2008 1:20:58 PM
Vial:	1:F,7	Acq. Method Set:	GPC3000 LCH Dual Wavelength
Injection #:	1	Date Processed:	8/28/2008 3:37:45 PM
Injection Volume:	10.00 ul	Processing Method:	ISOBM hCG Samples 082708
Run Time:	20.0 Minutes	Channel Name:	2487Channel 1
Sample Set Name:	GPC 082608 LCH Final	Proc. Chnl. Descr.:	280 nm

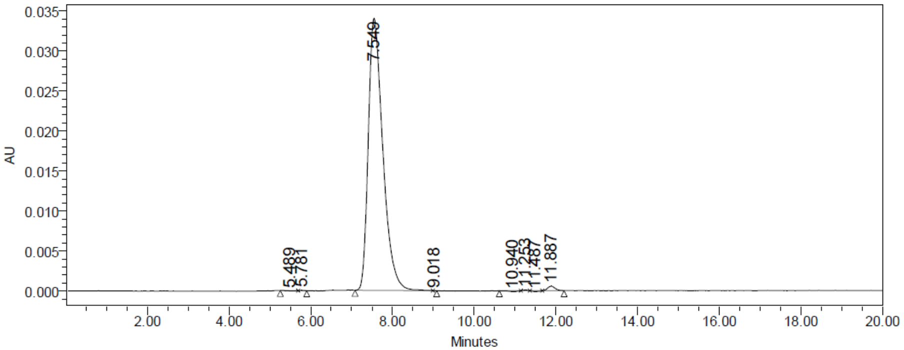

SAMPLE INFORMATION

Sample Name:	426	Acquired By:	harw ilc
Sample Type:	Unknow n	Date Acquired:	8/28/2008 2:23:42 PM
Vial:	1:F,8	Acq. Method Set:	GPC3000 LCH Dual Wavelength
Injection #:	1	Date Processed:	8/28/2008 3:37:45 PM
Injection Volume:	10.00 ul	Processing Method:	ISOBM hCG Samples 082708
Run Time:	20.0 Minutes	Channel Name:	2487Channel 1
Sample Set Name:	GPC 082608 LCH Final	Proc. Chnl. Descr.:	280 nm

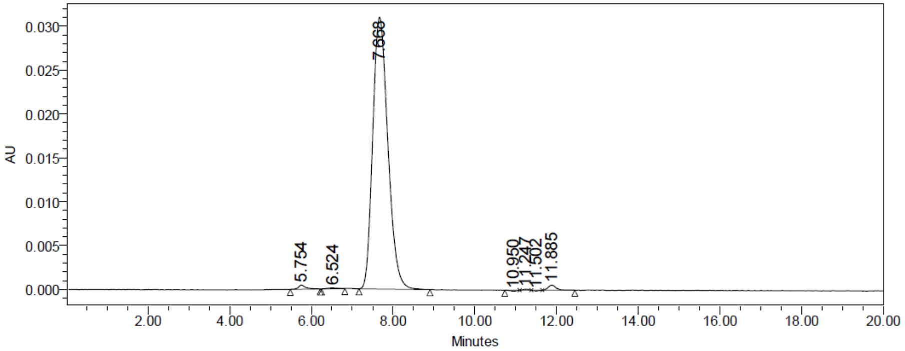

SAMPLE INFORMATION

Sample Name:	427	Acquired By:	harw ilc
Sample Type:	Unknow n	Date Acquired:	8/28/2008 3:26:28 PM
Vial:	2:A,1	Acq. Method Set:	GPC3000 LCH Dual Wavelength
Injection #:	1	Date Processed:	8/29/2008 9:09:11 AM
Injection Volume:	10.00 ul	Processing Method:	ISOBM hCG Samples 082708
Run Time:	20.0 Minutes	Channel Name:	2487Channel 1
Sample Set Name:	GPC 082608 LCH Final	Proc. Chnl. Descr.:	280 nm

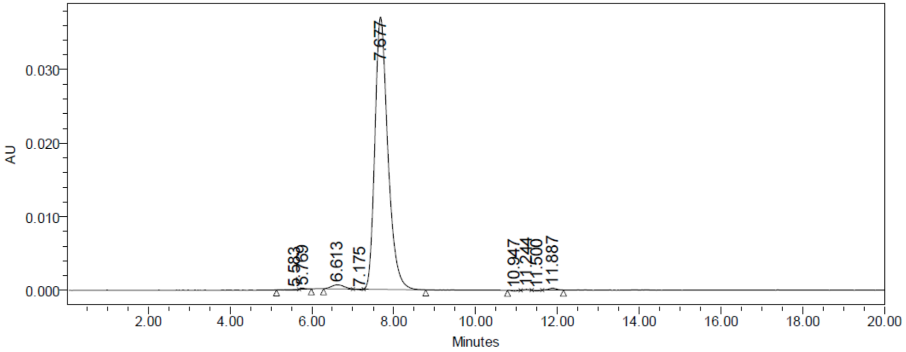

SAMPLE INFORMATION

Sample Name:	428	Acquired By:	harwilc
Sample Type:	Unknown	Date Acquired:	8/28/2008 4:29:08 PM
Vial:	2:A,2	Acq. Method Set:	GPC3000 LCH Dual Wavelength
Injection #:	1	Date Processed:	8/29/2008 9:09:12 AM
Injection Volume:	12.50 ul	Processing Method:	ISOBM hCG Samples 082708
Run Time:	20.0 Minutes	Channel Name:	2487Channel 1
Sample Set Name:	GPC 082608 LCH Final	Proc. Chnl. Descr.:	280 nm

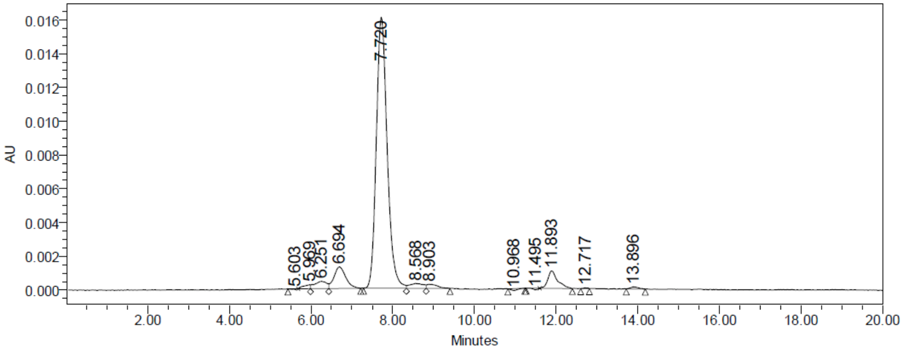

SAMPLE INFORMATION

Sample Name:	429	Acquired By:	harwilc
Sample Type:	Unknown	Date Acquired:	8/28/2008 5:31:53 PM
Vial:	2:A,3	Acq. Method Set:	GPC3000 LCH Dual Wavelength
Injection #:	1	Date Processed:	8/29/2008 9:09:12 AM
Injection Volume:	11.60 ul	Processing Method:	ISOBM hCG Samples 082708
Run Time:	20.0 Minutes	Channel Name:	2487Channel 1
Sample Set Name:	GPC 082608 LCH Final	Proc. Chnl. Descr.:	280 nm

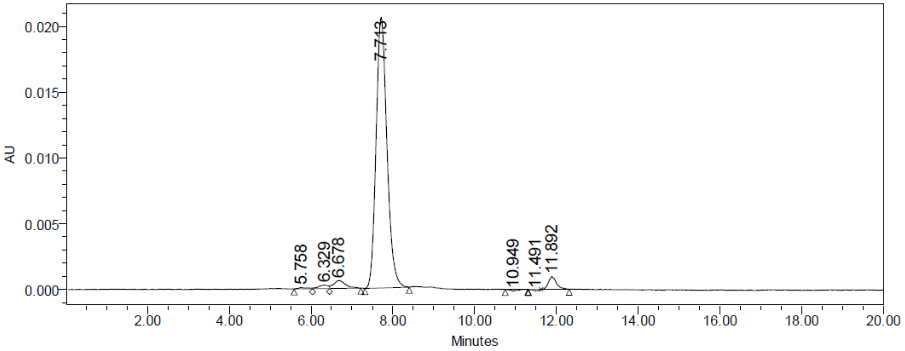

SAMPLE INFORMATION

Sample Name:	430	Acquired By:	harwilc
Sample Type:	Unknown	Date Acquired:	8/28/2008 6:34:37 PM
Vial:	2:A,4	Acq. Method Set:	GPC3000 LCH Dual Wavelength
Injection #:	1	Date Processed:	8/29/2008 9:09:12 AM
Injection Volume:	5.60 ul	Processing Method:	ISOBM hCG Samples 082708
Run Time:	20.0 Minutes	Channel Name:	2487Channel 1
Sample Set Name:	GPC 082608 LCH Final	Proc. Chnl. Descr.:	280 nm

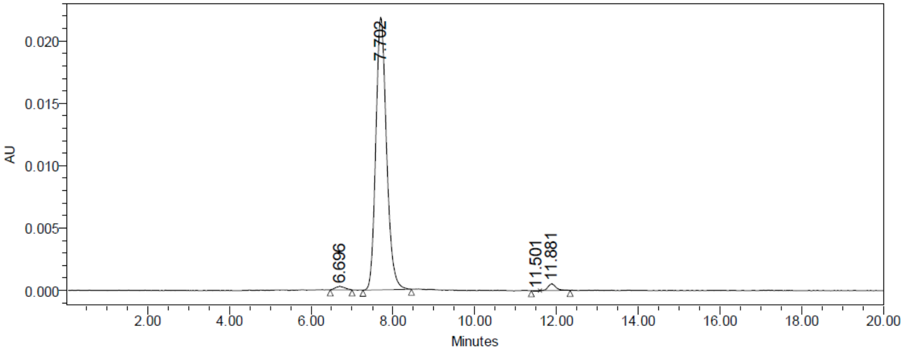

SAMPLE INFORMATION

Sample Name:	431	Acquired By:	harwilc
Sample Type:	Unknown	Date Acquired:	8/28/2008 7:37:23 PM
Vial:	2:A,5	Acq. Method Set:	GPC3000 LCH Dual Wavelength
Injection #:	1	Date Processed:	8/29/2008 9:09:13 AM
Injection Volume:	16.70 ul	Processing Method:	ISOBM hCG Samples 082708
Run Time:	20.0 Minutes	Channel Name:	2487Channel 1
Sample Set Name:	GPC 082608 LCH Final	Proc. Chnl. Descr.:	280 nm

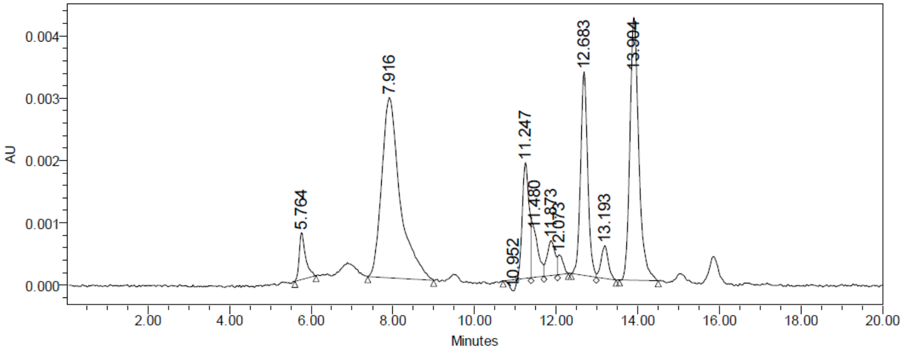

SAMPLE INFORMATION

Sample Name:	432	Acquired By:	harw ilc
Sample Type:	Unknow n	Date Acquired:	8/28/2008 8:40:09 PM
Vial:	2:A,6	Acq. Method Set:	GPC3000 LCH Dual Wavelength
Injection #:	1	Date Processed:	8/29/2008 9:09:13 AM
Injection Volume:	10.10 ul	Processing Method:	ISOBM hCG Samples 082708
Run Time:	20.0 Minutes	Channel Name:	2487Channel 1
Sample Set Name:	GPC 082608 LCH Final	Proc. Chnl. Descr.:	280 nm

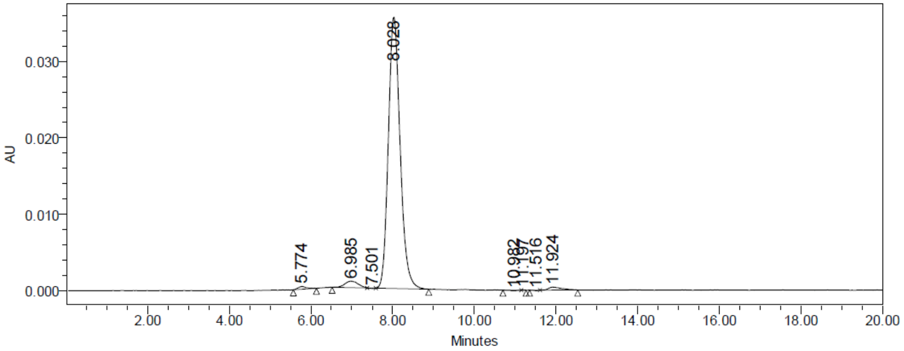

SAMPLE INFORMATION

Sample Name:	433	Acquired By:	harw ilc
Sample Type:	Unknow n	Date Acquired:	8/28/2008 9:42:57 PM
Vial:	2:A,7	Acq. Method Set:	GPC3000 LCH Dual Wavelength
Injection #:	1	Date Processed:	8/29/2008 9:09:13 AM
Injection Volume:	10.00 ul	Processing Method:	ISOBM hCG Samples 082708
Run Time:	20.0 Minutes	Channel Name:	2487Channel 1
Sample Set Name:	GPC 082608 LCH Final	Proc. Chnl. Descr.:	280 nm

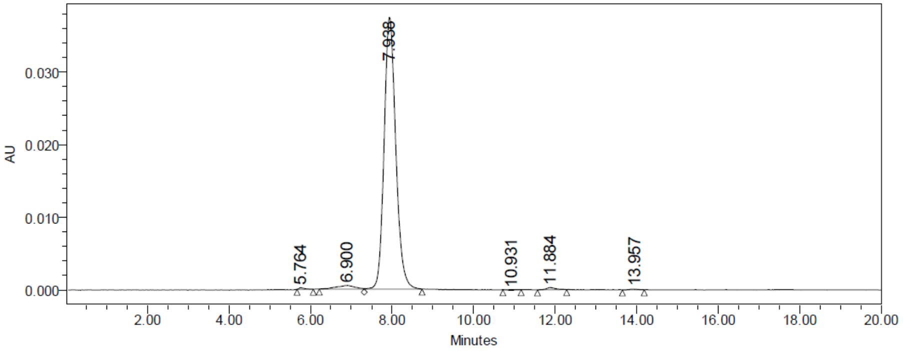

SAMPLE INFORMATION

Sample Name:	434	Acquired By:	harw ilc
Sample Type:	Unknow n	Date Acquired:	8/28/2008 10:45:48 PM
Vial:	2:A,8	Acq. Method Set:	GPC3000 LCH Dual Wavelength
Injection #:	1	Date Processed:	8/29/2008 9:09:13 AM
Injection Volume:	11.10 ul	Processing Method:	ISOBM hCG Samples 082708
Run Time:	20.0 Minutes	Channel Name:	2487Channel 1
Sample Set Name:	GPC 082608 LCH Final	Proc. Chnl. Descr.:	280 nm

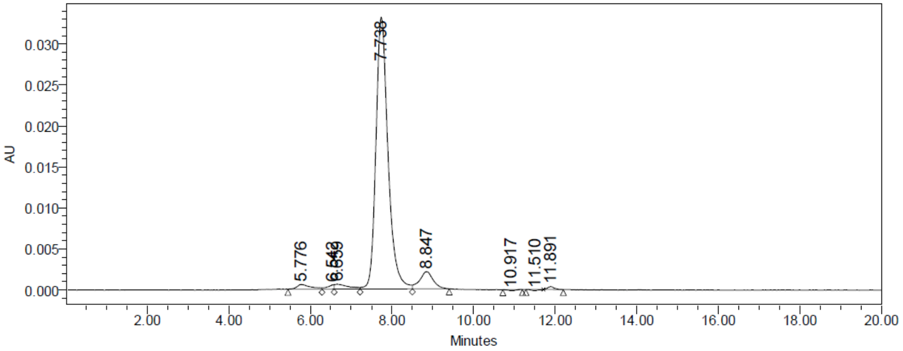

SAMPLE INFORMATION

Sample Name:	435	Acquired By:	harw ilc
Sample Type:	Unknow n	Date Acquired:	8/28/2008 11:48:34 PM
Vial:	2:B,1	Acq. Method Set:	GPC3000 LCH Dual Wavelength
Injection #:	1	Date Processed:	8/29/2008 9:09:13 AM
Injection Volume:	11.50 ul	Processing Method:	ISOBM hCG Samples 082708
Run Time:	20.0 Minutes	Channel Name:	2487Channel 1
Sample Set Name:	GPC 082608 LCH Final	Proc. Chnl. Descr.:	280 nm

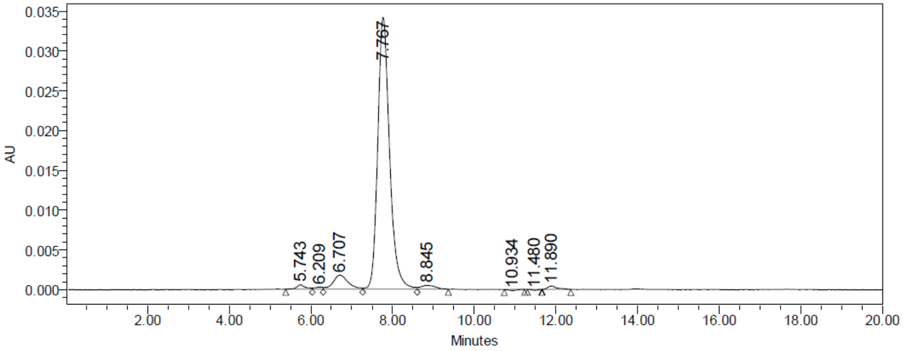

SAMPLE INFORMATION

Sample Name:	436	Acquired By:	harw ilc
Sample Type:	Unknow n	Date Acquired:	8/29/2008 12:51:15 AM
Vial:	2:B,2	Acq. Method Set:	GPC3000 LCH Dual Wavelength
Injection #:	1	Date Processed:	8/29/2008 9:09:14 AM
Injection Volume:	10.00 ul	Processing Method:	ISOBM hCG Samples 082708
Run Time:	20.0 Minutes	Channel Name:	2487Channel 1
Sample Set Name:	GPC 082608 LCH Final	Proc. Chnl. Descr.:	280 nm

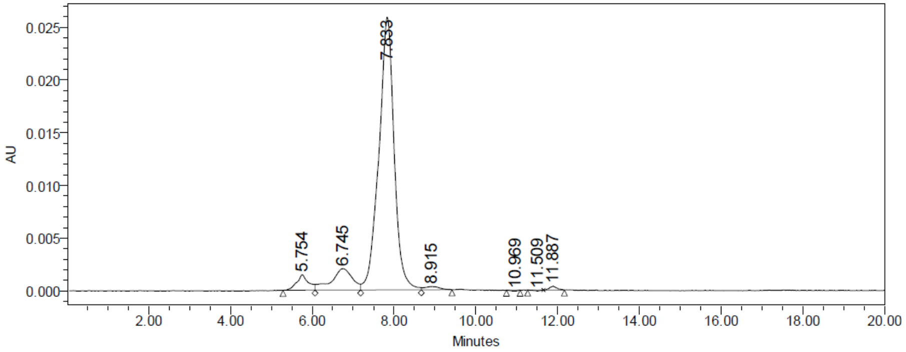

SAMPLE INFORMATION

Sample Name:	437	Acquired By:	harw ilc
Sample Type:	Unknow n	Date Acquired:	8/29/2008 1:53:55 AM
Vial:	2:B,3	Acq. Method Set:	GPC3000 LCH Dual Wavelength
Injection #:	1	Date Processed:	8/29/2008 9:09:14 AM
Injection Volume:	10.00 ul	Processing Method:	ISOBM hCG Samples 082708
Run Time:	20.0 Minutes	Channel Name:	2487Channel 1
Sample Set Name:	GPC 082608 LCH Final	Proc. Chnl. Descr.:	280 nm

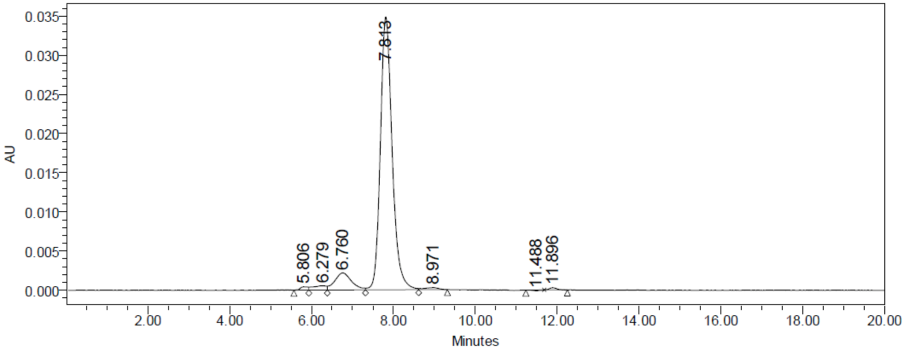

SAMPLE INFORMATION

Sample Name:	438	Acquired By:	harw ilc
Sample Type:	Unknow n	Date Acquired:	8/29/2008 2:56:37 AM
Vial:	2:B,4	Acq. Method Set:	GPC3000 LCH Dual Wavelength
Injection #:	1	Date Processed:	8/29/2008 9:09:14 AM
Injection Volume:	10.00 ul	Processing Method:	ISOBM hCG Samples 082708
Run Time:	20.0 Minutes	Channel Name:	2487Channel 1
Sample Set Name:	GPC 082608 LCH Final	Proc. Chnl. Descr.:	280 nm

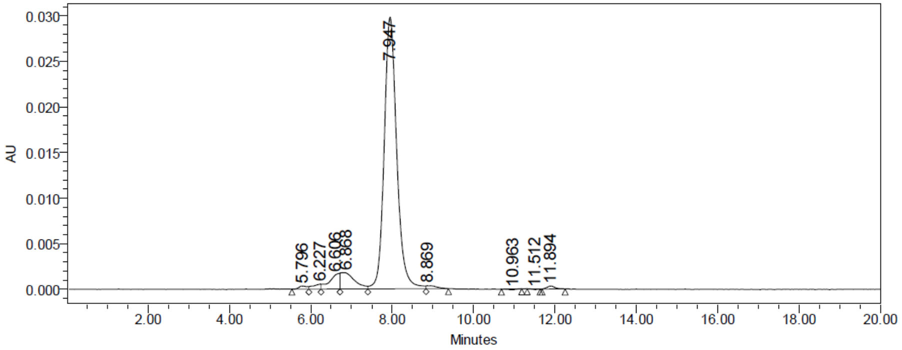

SAMPLE INFORMATION

Sample Name:	439	Acquired By:	harw ilc
Sample Type:	Unknow n	Date Acquired:	8/29/2008 3:59:23 AM
Vial:	2:B,5	Acq. Method Set:	GPC3000 LCH Dual Wavelength
Injection #:	1	Date Processed:	8/29/2008 9:09:14 AM
Injection Volume:	12.80 ul	Processing Method:	ISOBM hCG Samples 082708
Run Time:	20.0 Minutes	Channel Name:	2487Channel 1
Sample Set Name:	GPC 082608 LCH Final	Proc. Chnl. Descr.:	280 nm

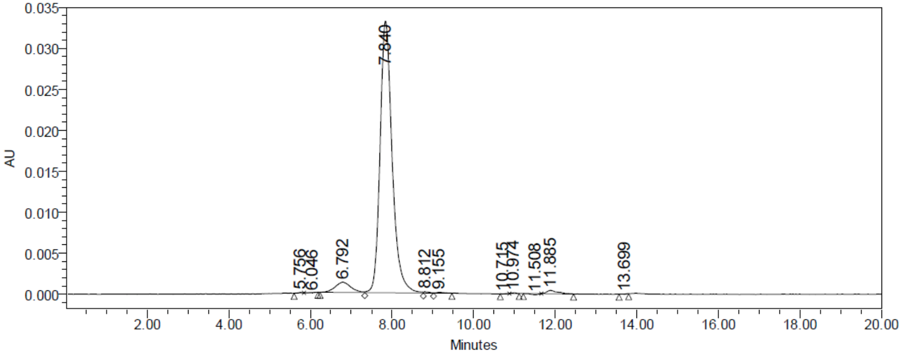

SAMPLE INFORMATION

Sample Name:	440	Acquired By:	harwilc
Sample Type:	Unknown	Date Acquired:	8/29/2008 5:02:09 AM
Vial:	2:B,6	Acq. Method Set:	GPC3000 LCH Dual Wavelength
Injection #:	1	Date Processed:	8/29/2008 9:09:14 AM
Injection Volume:	10.00 ul	Processing Method:	ISOBM hCG Samples 082708
Run Time:	20.0 Minutes	Channel Name:	2487Channel 1
Sample Set Name:	GPC 082608 LCH Final	Proc. Chnl. Descr.:	280 nm

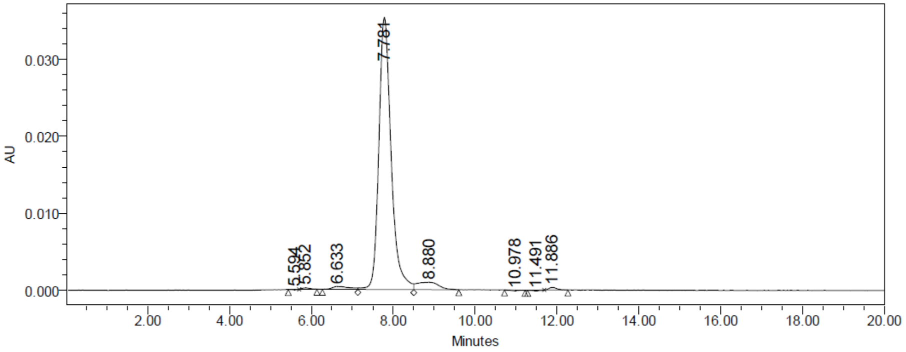

SAMPLE INFORMATION

Sample Name:	441	Acquired By:	harwilc
Sample Type:	Unknown	Date Acquired:	8/29/2008 6:04:55 AM
Vial:	2:B,7	Acq. Method Set:	GPC3000 LCH Dual Wavelength
Injection #:	1	Date Processed:	8/29/2008 9:09:15 AM
Injection Volume:	10.60 ul	Processing Method:	ISOBM hCG Samples 082708
Run Time:	20.0 Minutes	Channel Name:	2487Channel 1
Sample Set Name:	GPC 082608 LCH Final	Proc. Chnl. Descr.:	280 nm

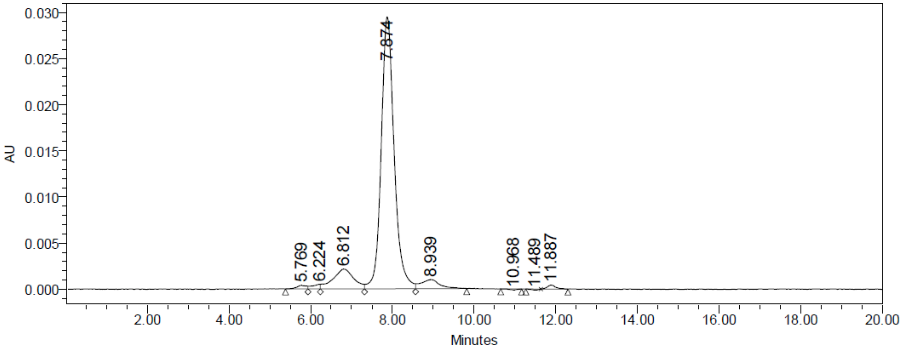

SAMPLE INFORMATION

Sample Name:	442	Acquired By:	harwilc
Sample Type:	Unknown	Date Acquired:	8/29/2008 7:07:44 AM
Vial:	2:B,8	Acq. Method Set:	GPC3000 LCH Dual Wavelength
Injection #:	1	Date Processed:	8/29/2008 9:09:15 AM
Injection Volume:	10.00 ul	Processing Method:	ISOBM hCG Samples 082708
Run Time:	20.0 Minutes	Channel Name:	2487Channel 1
Sample Set Name:	GPC 082608 LCH Final	Proc. Chnl. Descr.:	280 nm

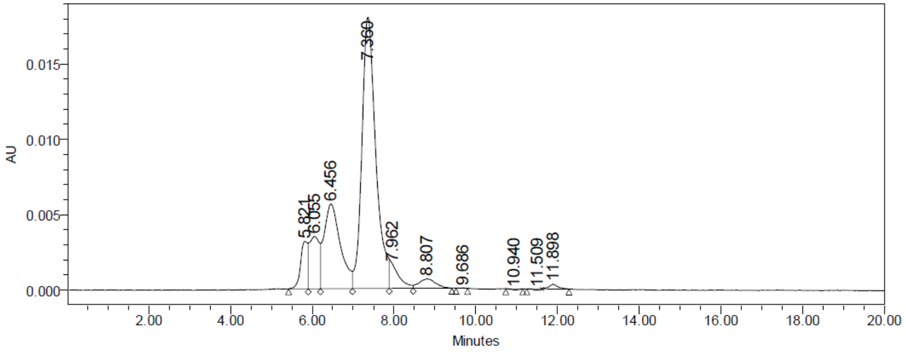

SAMPLE INFORMATION

Sample Name:	443	Acquired By:	harwilc
Sample Type:	Unknown	Date Acquired:	8/29/2008 8:10:29 AM
Vial:	2:C,1	Acq. Method Set:	GPC3000 LCH Dual Wavelength
Injection #:	1	Date Processed:	8/29/2008 9:09:15 AM
Injection Volume:	10.00 ul	Processing Method:	ISOBM hCG Samples 082708
Run Time:	20.0 Minutes	Channel Name:	2487Channel 1
Sample Set Name:	GPC 082608 LCH Final	Proc. Chnl. Descr.:	280 nm

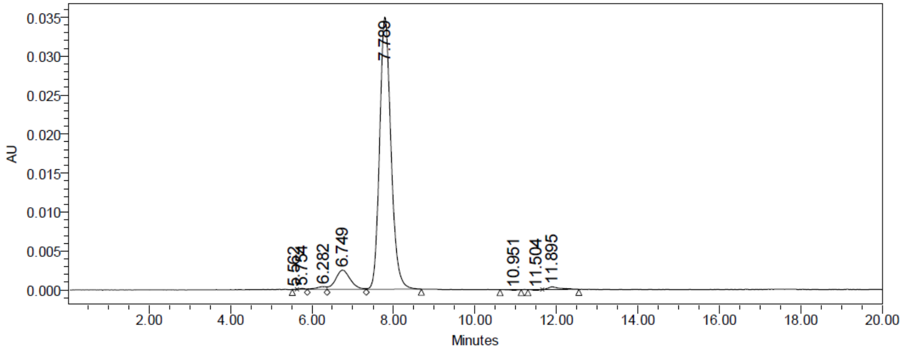

SAMPLE INFORMATION

Sample Name:	444	Acquired By:	harwilc
Sample Type:	Unknown	Date Acquired:	8/29/2008 9:13:09 AM
Vial:	2:C,2	Acq. Method Set:	GPC3000 LCH Dual Wavelength
Injection #:	1	Date Processed:	9/2/2008 9:12:50 AM
Injection Volume:	10.10 ul	Processing Method:	ISOBM hCG Samples 082708
Run Time:	20.0 Minutes	Channel Name:	2487Channel 1
Sample Set Name:	GPC 082608 LCH Final	Proc. Chnl. Descr.:	280 nm

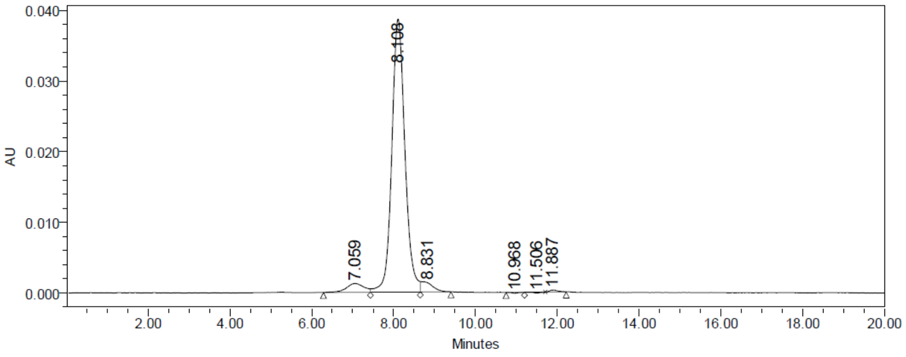

SAMPLE INFORMATION

Sample Name:	445	Acquired By:	harwilc
Sample Type:	Unknown	Date Acquired:	8/29/2008 10:15:47 AM
Vial:	2:C,3	Acq. Method Set:	GPC3000 LCH Dual Wavelength
Injection #:	1	Date Processed:	9/2/2008 9:12:50 AM
Injection Volume:	11.90 ul	Processing Method:	ISOBM hCG Samples 082708
Run Time:	20.0 Minutes	Channel Name:	2487Channel 1
Sample Set Name:	GPC 082608 LCH Final	Proc. Chnl. Descr.:	280 nm

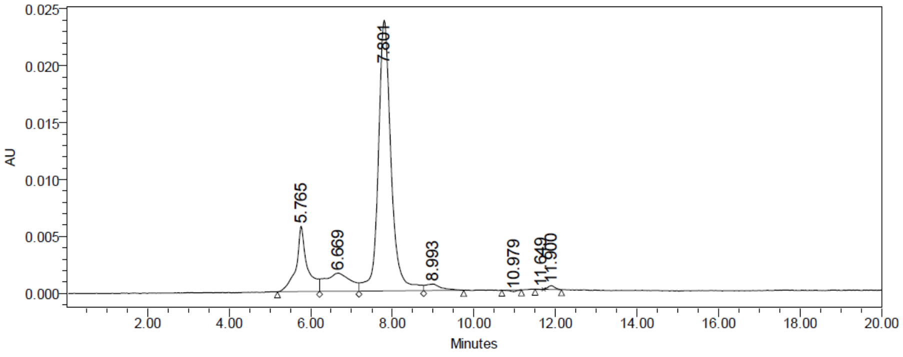

SAMPLE INFORMATION

Sample Name:	446	Acquired By:	harwilc
Sample Type:	Unknown	Date Acquired:	8/29/2008 11:18:31 AM
Vial:	2:C,4	Acq. Method Set:	GPC3000 LCH Dual Wavelength
Injection #:	1	Date Processed:	9/2/2008 9:12:50 AM
Injection Volume:	10.00 ul	Processing Method:	ISOBM hCG Samples 082708
Run Time:	20.0 Minutes	Channel Name:	2487Channel 1
Sample Set Name:	GPC 082608 LCH Final	Proc. Chnl. Descr.:	280 nm

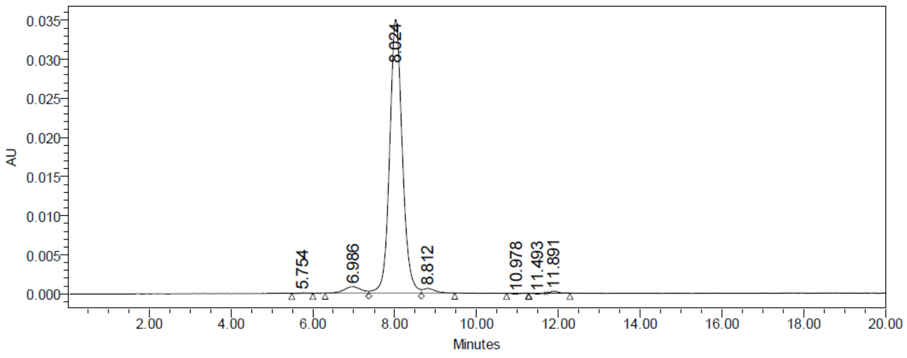

SAMPLE INFORMATION

Sample Name:	447	Acquired By:	harwilc
Sample Type:	Unknown	Date Acquired:	8/29/2008 12:21:15 PM
Vial:	2:C,5	Acq. Method Set:	GPC3000 LCH Dual Wavelength
Injection #:	1	Date Processed:	9/2/2008 9:12:51 AM
Injection Volume:	11.80 ul	Processing Method:	ISOBM hCG Samples 082708
Run Time:	20.0 Minutes	Channel Name:	2487Channel 1
Sample Set Name:	GPC 082608 LCH Final	Proc. Chnl. Descr.:	280 nm

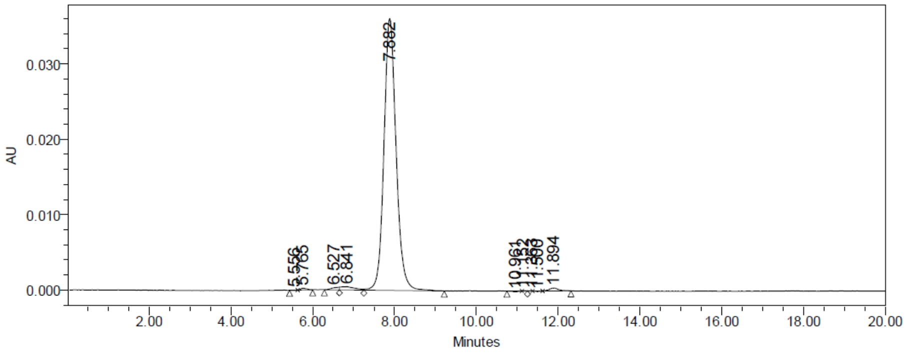

SAMPLE INFORMATION

Sample Name:	448	Acquired By:	harwilc
Sample Type:	Unknown	Date Acquired:	8/29/2008 1:23:56 PM
Vial:	2:C,6	Acq. Method Set:	GPC3000 LCH Dual Wavelength
Injection #:	1	Date Processed:	9/2/2008 9:12:51 AM
Injection Volume:	10.00 ul	Processing Method:	ISOBM hCG Samples 082708
Run Time:	20.0 Minutes	Channel Name:	2487Channel 1
Sample Set Name:	GPC 082608 LCH Final	Proc. Chnl. Descr.:	280 nm

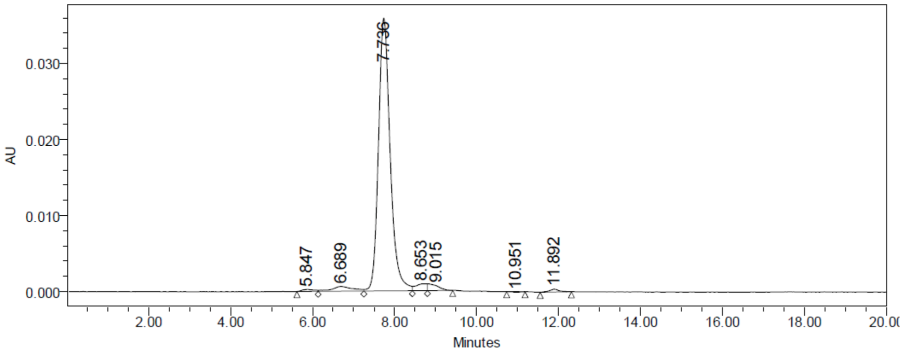

SAMPLE INFORMATION

Sample Name:	449	Acquired By:	harwilc
Sample Type:	Unknown	Date Acquired:	8/29/2008 2:26:49 PM
Vial:	2:C,7	Acq. Method Set:	GPC3000 LCH Dual Wavelength
Injection #:	1	Date Processed:	9/2/2008 9:12:51 AM
Injection Volume:	10.00 ul	Processing Method:	ISOBM hCG Samples 082708
Run Time:	20.0 Minutes	Channel Name:	2487Channel 1
Sample Set Name:	GPC 082608 LCH Final	Proc. Chnl. Descr.:	280 nm

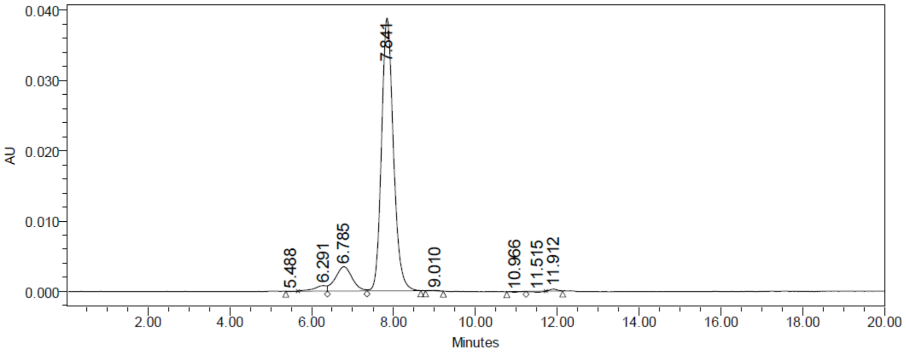

SAMPLE INFORMATION

Sample Name:	450	Acquired By:	harwilc
Sample Type:	Unknown	Date Acquired:	8/29/2008 3:29:36 PM
Vial:	2:C,8	Acq. Method Set:	GPC3000 LCH Dual Wavelength
Injection #:	1	Date Processed:	9/2/2008 9:12:51 AM
Injection Volume:	11.10 ul	Processing Method:	ISOBM hCG Samples 082708
Run Time:	20.0 Minutes	Channel Name:	2487Channel 1
Sample Set Name:	GPC 082608 LCH Final	Proc. Chnl. Descr.:	280 nm

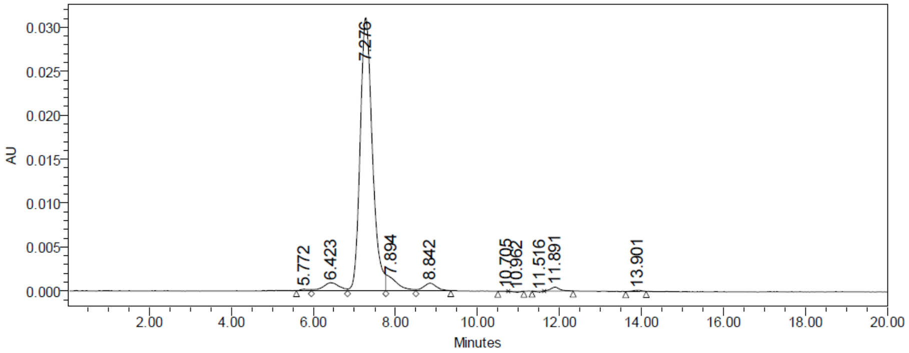

SAMPLE INFORMATION

Sample Name:	0.1% DTT	Acquired By:	harwilc
Sample Type:	Unknown	Date Acquired:	8/29/2008 5:56:11 PM
Vial:	2:D,2	Acq. Method Set:	GPC3000 LCH Dual Wavelength
Injection #:	1	Date Processed:	9/2/2008 9:12:52 AM
Injection Volume:	10.00 ul	Processing Method:	ISOBM hCG Samples 082708
Run Time:	20.0 Minutes	Channel Name:	2487Channel 1
Sample Set Name:	GPC 082608 LCH Final	Proc. Chnl. Descr.:	280 nm

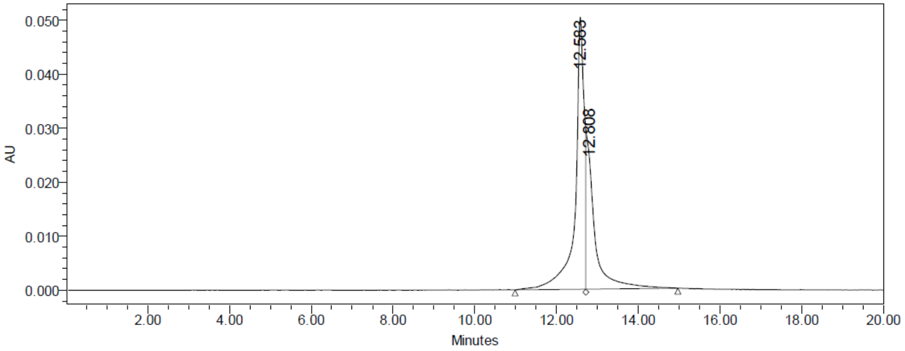

SAMPLE INFORMATION

Sample Name:	0.1% Sodium Azide	Acquired By:	harw ilc
Sample Type:	Unknow n	Date Acquired:	8/29/2008 6:17:09 PM
Vial:	2:D,3	Acq. Method Set:	GPC3000 LCH Dual Wavelength
Injection #:	1	Date Processed:	9/2/2008 9:12:52 AM
Injection Volume:	10.00 ul	Processing Method:	ISOBM hCG Samples 082708
Run Time:	20.0 Minutes	Channel Name:	2487Channel 1
Sample Set Name:	GPC 082608 LCH Final	Proc. Chnl. Descr.:	280 nm

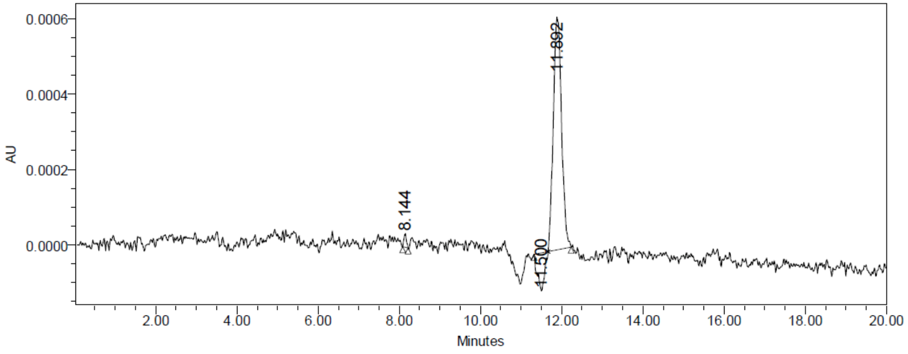

Online Resource 16b

ISOBM-Abs: Biochemical characterization by non-reducing SDS-PAGE (NRH)

SDS-PAGE gradient gels 4-12%. 5µg antibody per lane.

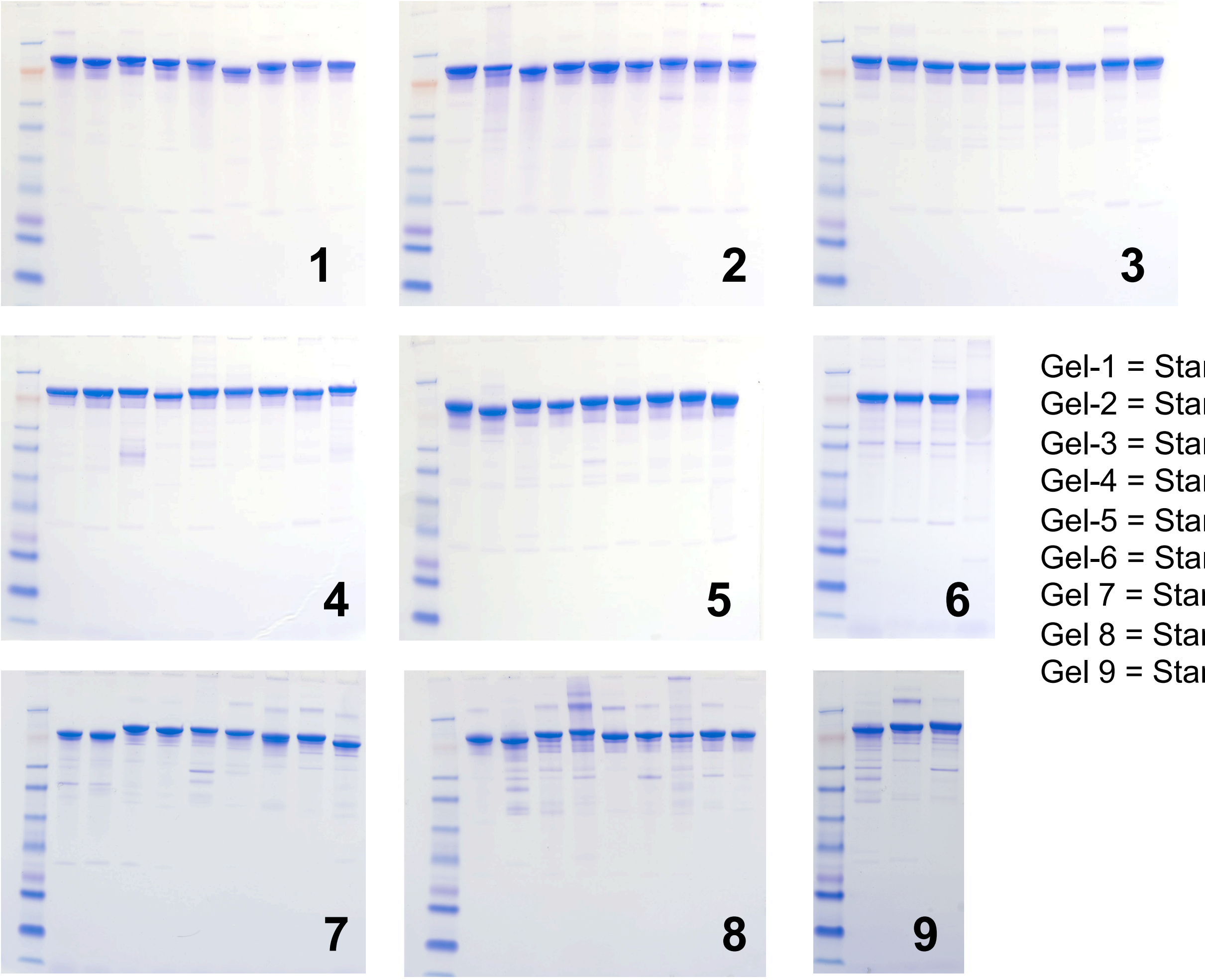

Gel-1 = Standard, ISOBM 382-390,
Gel-2 = Standard, ISOBM 391-399,
Gel-3 = Standard, ISOBM 400-408,
Gel-4 = Standard, ISOBM 409-417,
Gel-5 = Standard, ISOBM 418-421, 423-427,
Gel-6 = Standard, ISOBM 428-431
Gel 7 = Standard, ISOBM 428 – 429 + 432 - 438
Gel 8 = Standard, ISOBM 439 - 447
Gel 9 = Standard, ISOBM 448 – 450

Note!
428-430 are sheep monoclonal antibodies
431 is a affinity purified sheep polyclonal antibody

Online Resource 16b

ISOBM-Abs: Biochemical characterization by reducing SDS-PAGE (ABB)

Three mAbs (ISOBM-385, 426 and 448) have double heavy chains, seven Abs (ISOBM-382, 388, 400, 406, 440, 442 and 450) have double light chains and one Ab (ISOBM-436) has both double light and double heavy chains.

Fourteen Abs (ISOBM-411, 417, 428, 434, 435 Figure 3F, 436, 438, 439, 440, 441, 442, 444, 445 and 448 seem to have some albumin impurities.

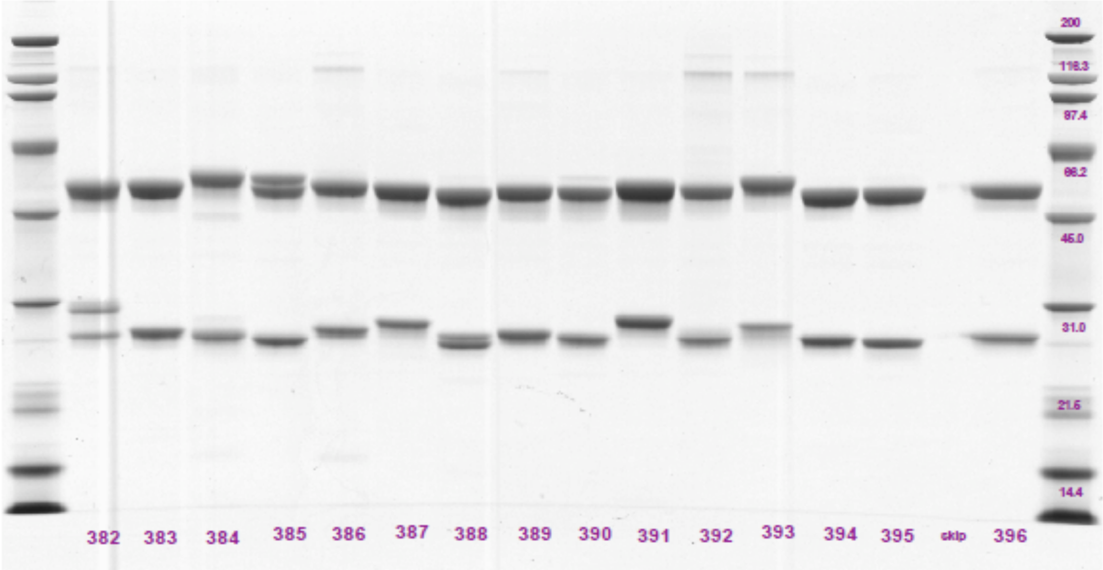
200
116.3
97.4
66.2
45.0
31.0
21.5
14.4
382
383
384
385
386
387
388
389
390
391
392
393
394
395
skip
396

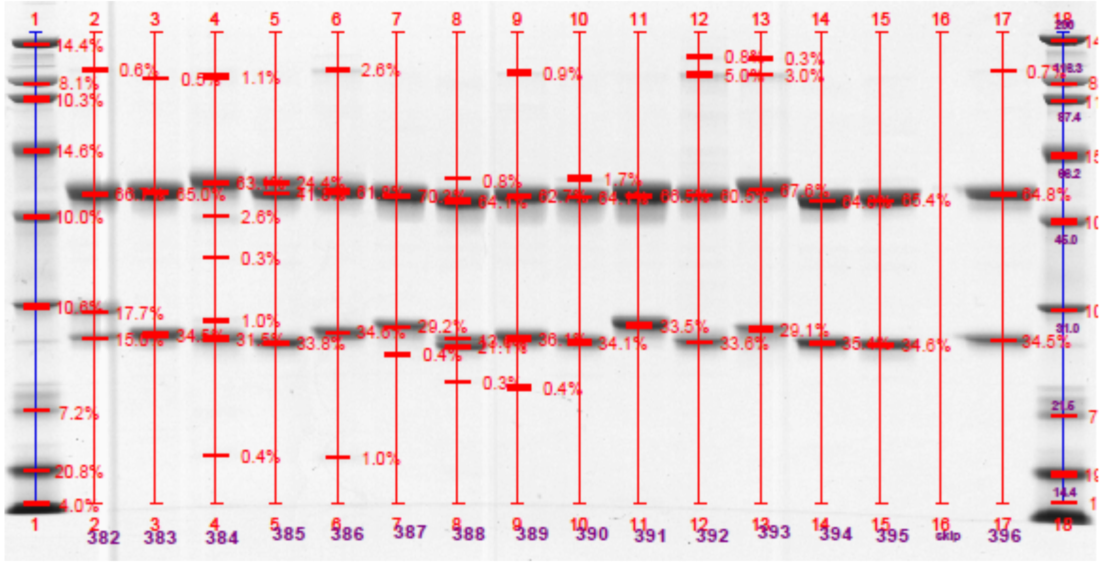
382
383
384
385
386
387
388
389
390
391
392
393
394
395
skip
396

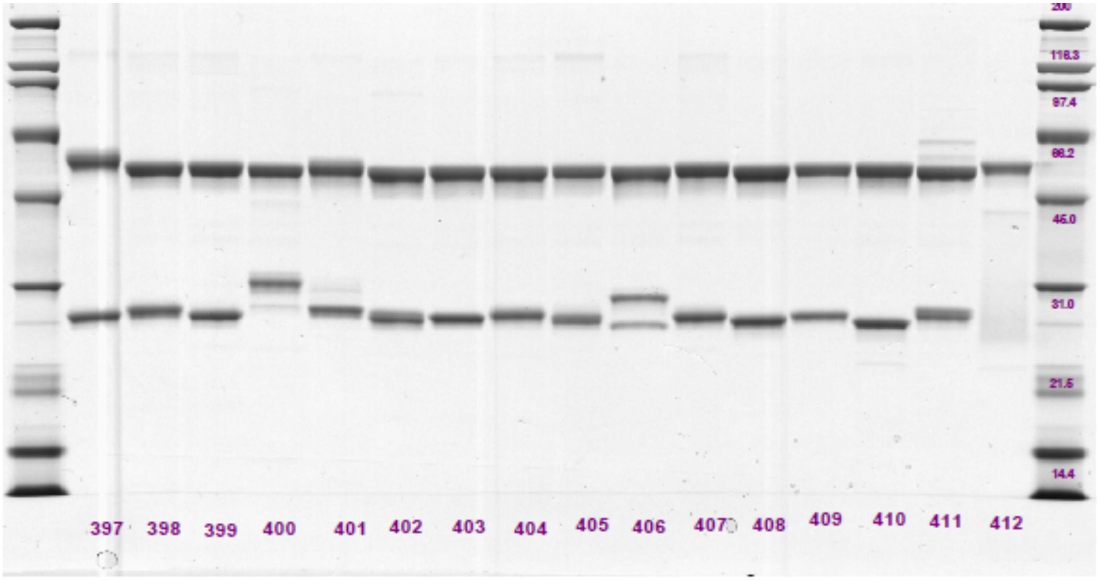
200
116.3
97.4
66.2
45.0
31.0
21.5
14.4
397 398 399 400 401 402 403 404 405 406 407 408 409 410 411 412

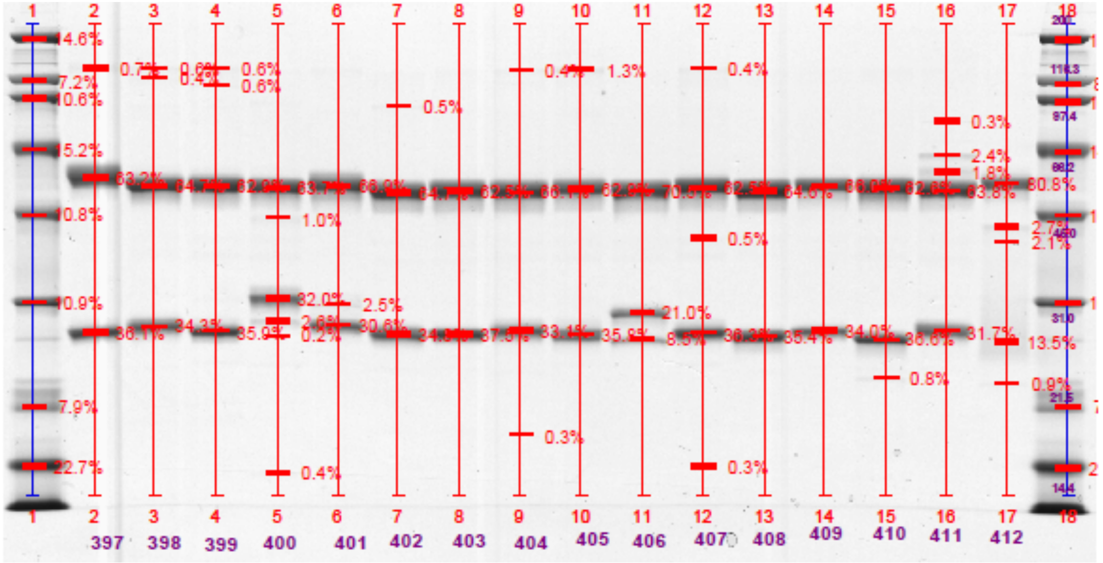
1 2 3 4 5 6 7 8 9 10 11 12 13 14 15 16 17 18
397 398 399 400 401 402 403 404 405 406 407 408 409 410 411 412

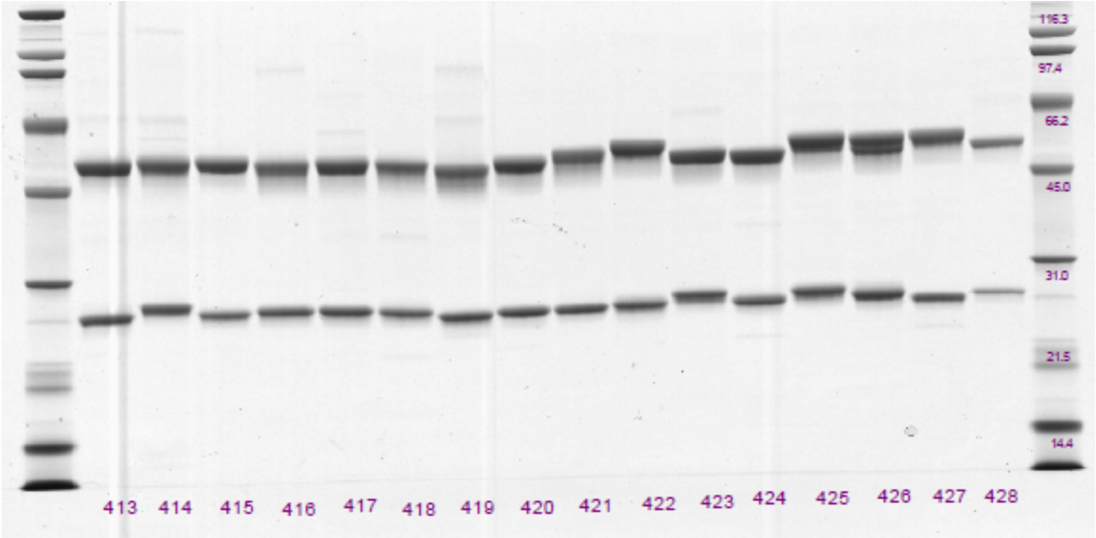
116.3
97.4
66.2
45.0
31.0
21.5
14.4
413
414
415
416
417
418
419
420
421
422
423
424
425
426
427
428

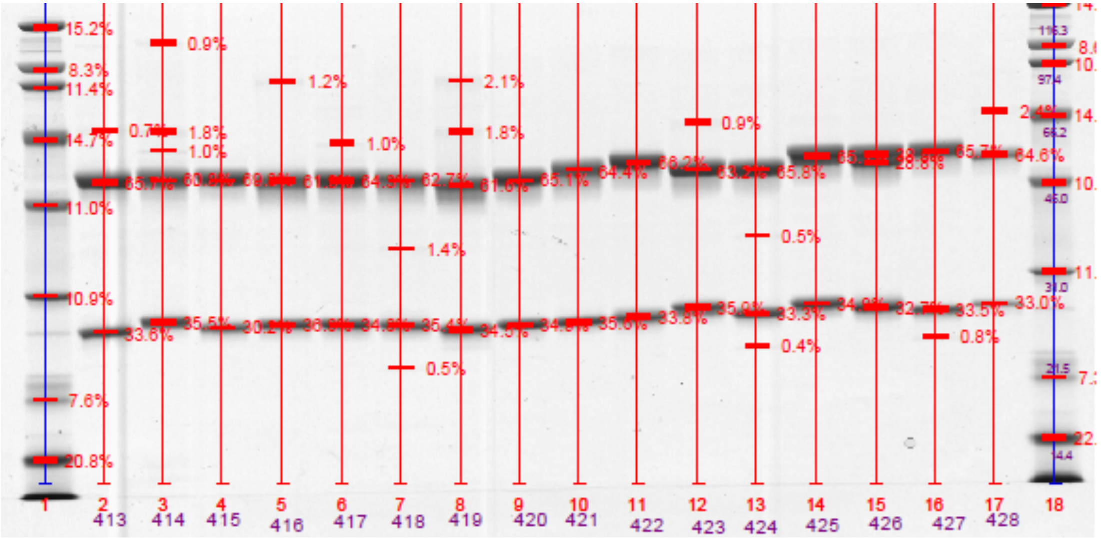
15.2%
8.3%
11.4%
14.7%
11.0%
10.9%
7.6%
20.8%
0.9%
1.8%
1.0%
1.2%
1.0%
1.4%
0.5%
2.1%
1.8%
0.9%
0.5%
0.4%
0.8%
33.6%
35.5%
33.0%
1 2 3 4 5 6 7 8 9 10 11 12 13 14 15 16 17 18
413 414 415 416 417 418 419 420 421 422 423 424 425 426 427 428

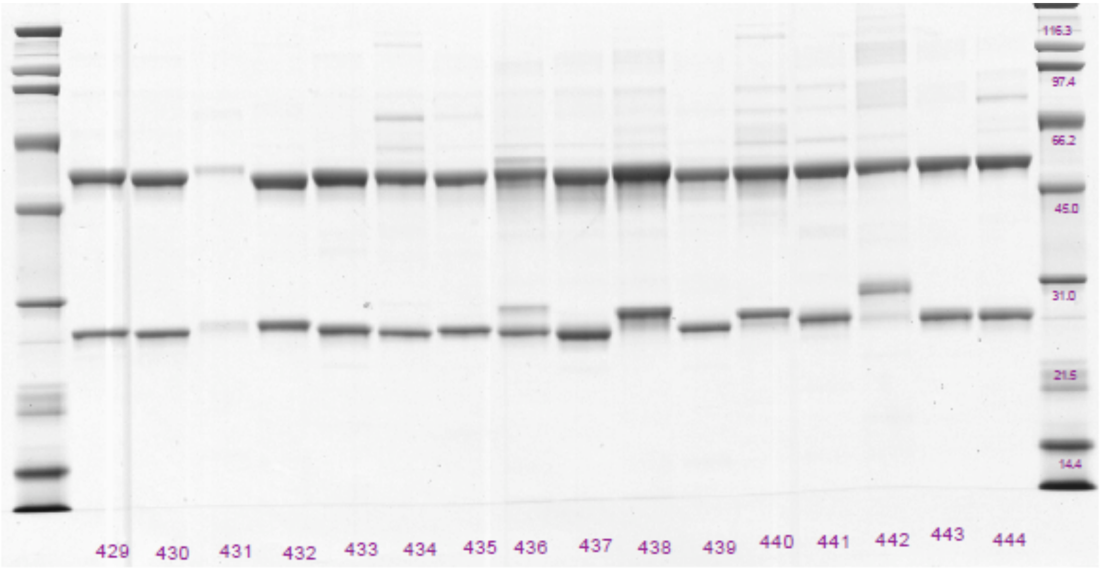
116.3
97.4
66.2
45.0
31.0
21.5
14.4
429
430
431
432
433
434
435
436
437
438
439
440
441
442
443
444

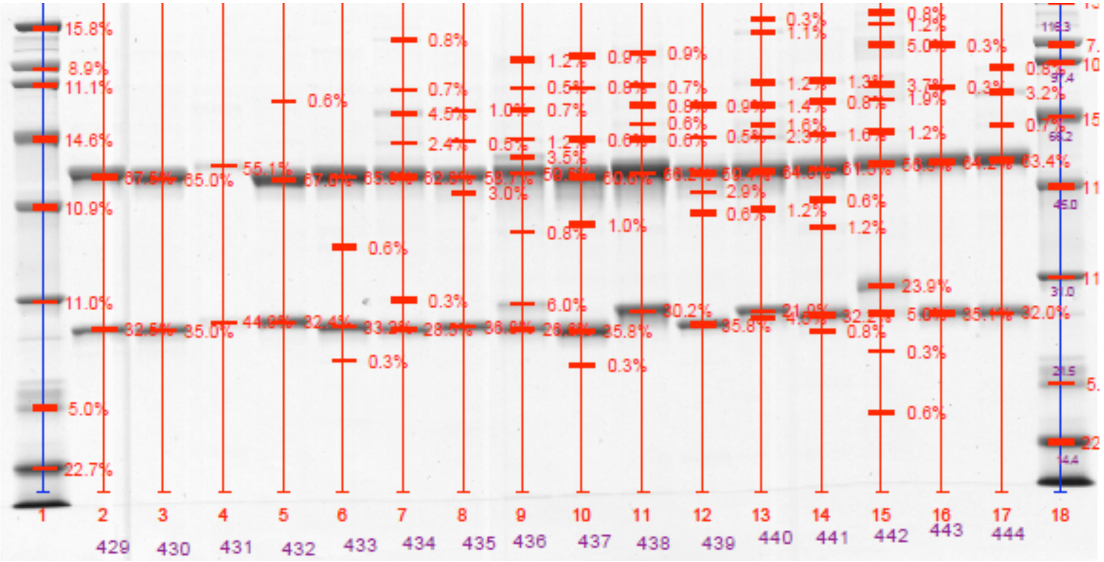
1
2
3
4
5
6
7
8
9
10
11
12
13
14
15
16
17
18
429
430
431
432
433
434
435
436
437
438
439
440
441
442
443
444

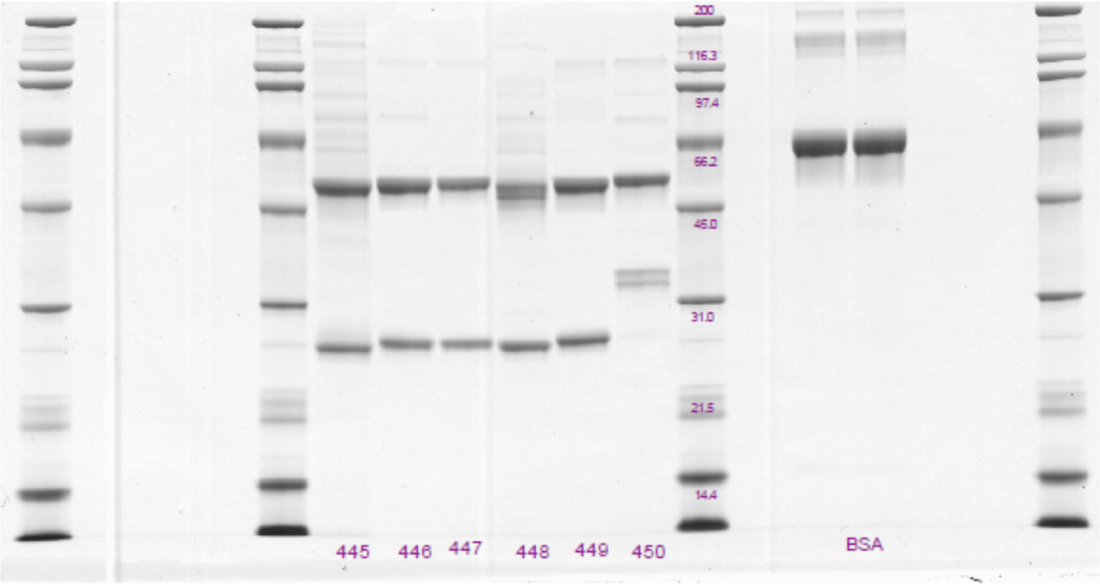
200
116.3
97.4
66.2
45.0
31.0
21.5
14.4
445
446
447
448
449
450
BSA

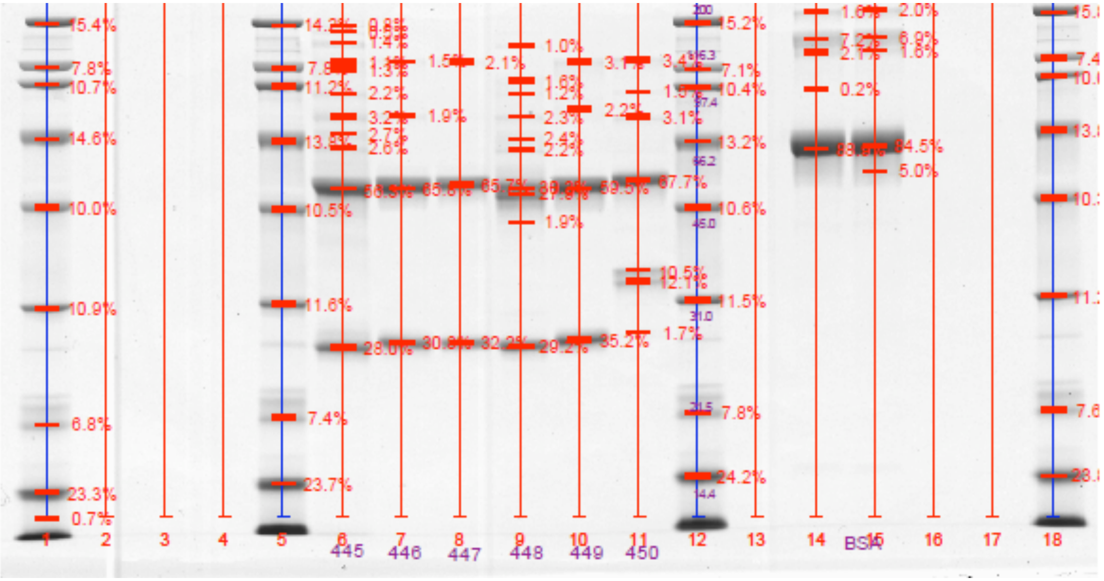
1
2
3
4
5
6
7
8
9
10
11
12
13
14
15
16
17
18
445
446
447
448
449
450
BSA
200
116.3
97.4
66.2
45.0
31.0
21.5
14.4

Online Resource 17

ISOBM-Abs: Analytical Analyses by Phastsystem Isoelectric Focusing

Acidic Positions:

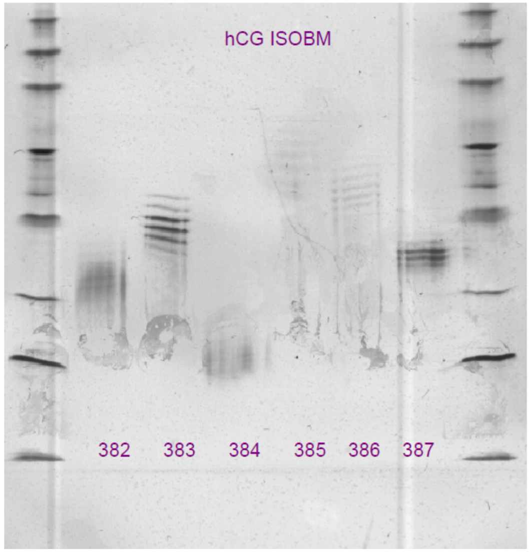

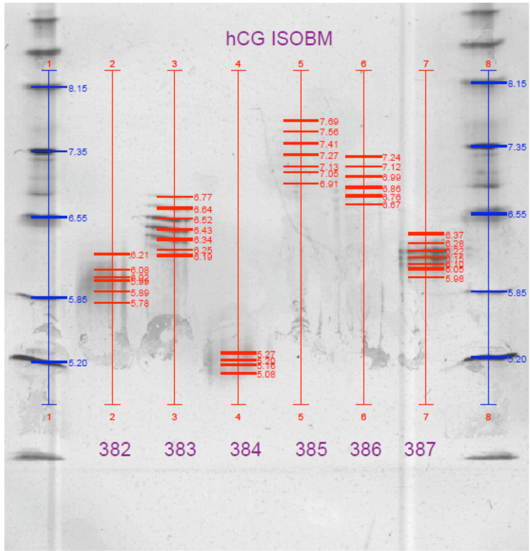

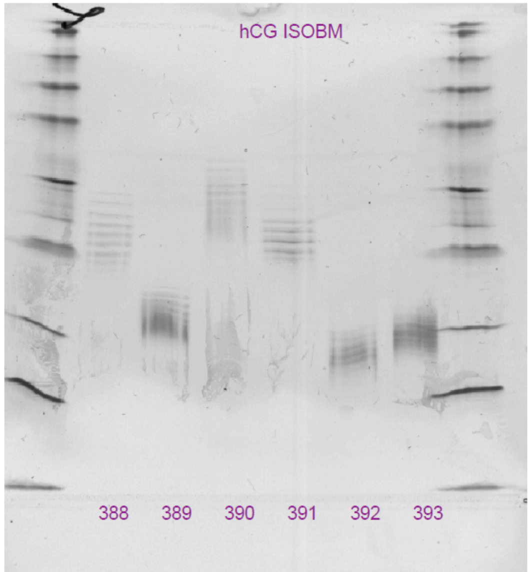

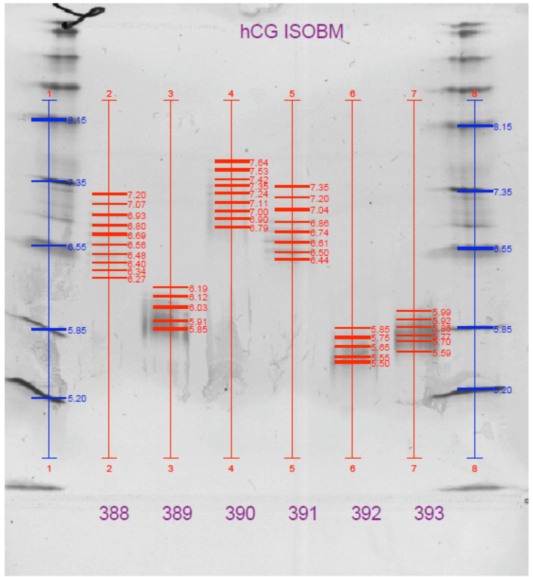

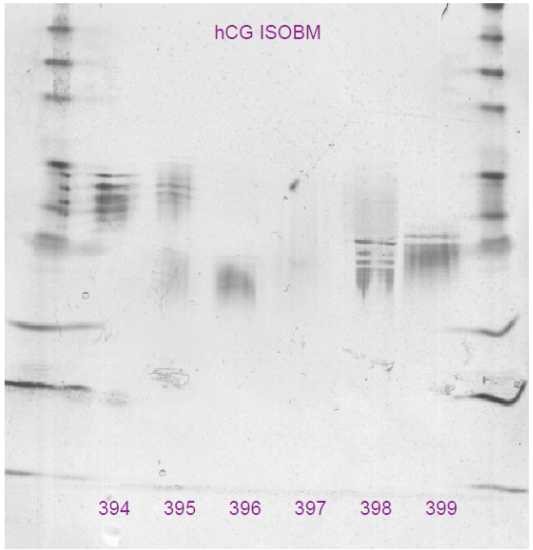

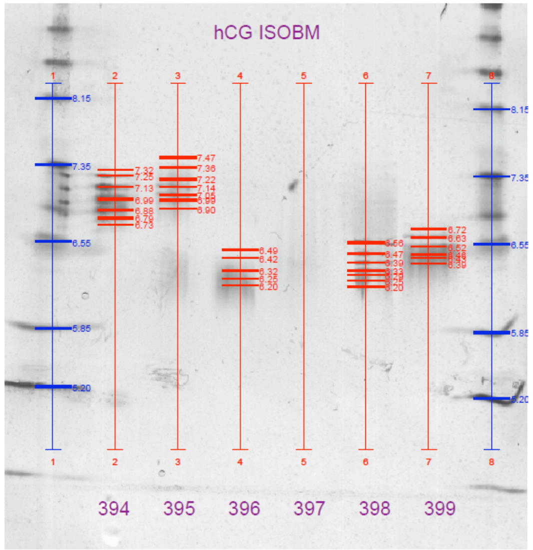

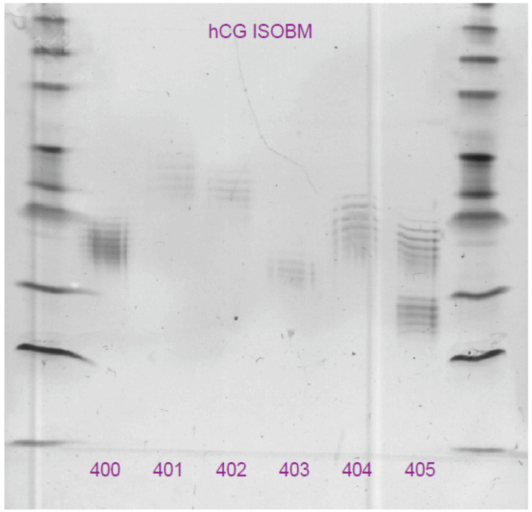
hCG ISOBM
400 401 402 403 404 405

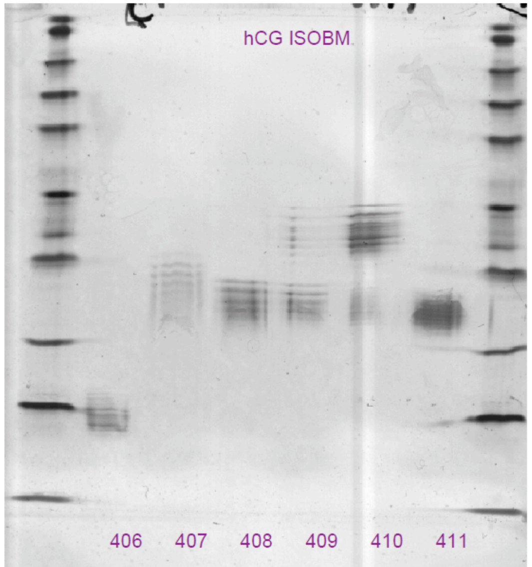
hCG ISOBM
406 407 408 409 410 411

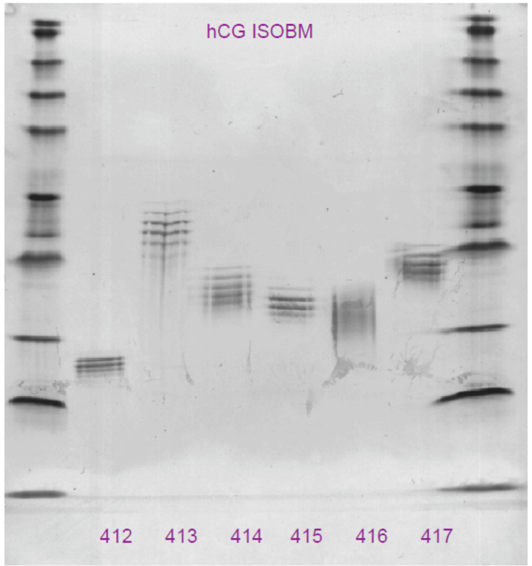
hCG ISOBM
412 413 414 415 416 417

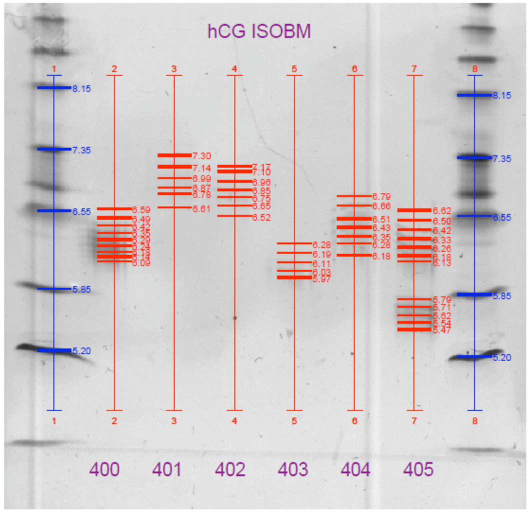
hCG ISOBM
400 401 402 403 404 405

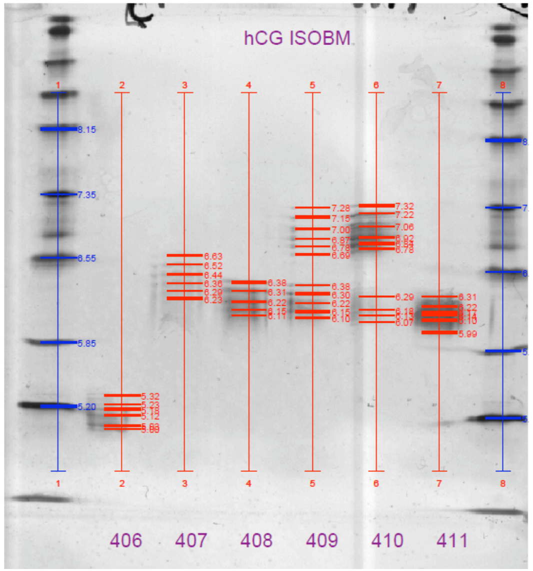
hCG ISOBM
406 407 408 409 410 411

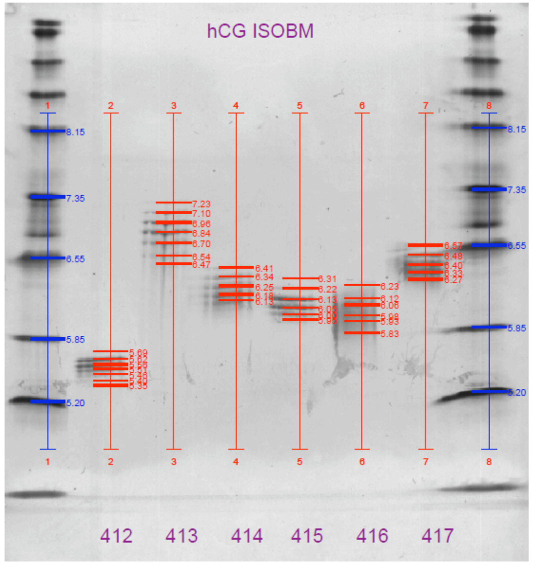
hCG ISOBM
412 413 414 415 416 417

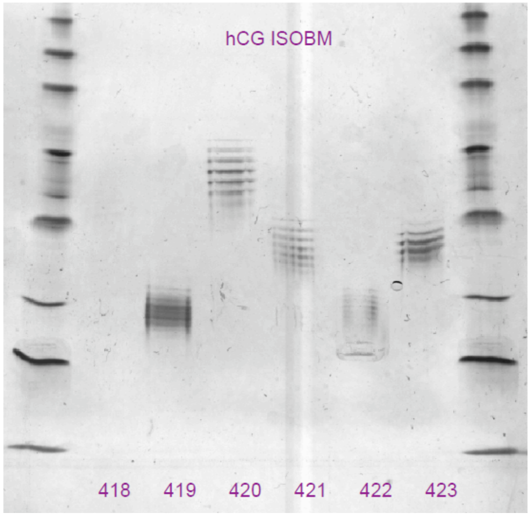
hCG ISOBM
418 419 420 421 422 423

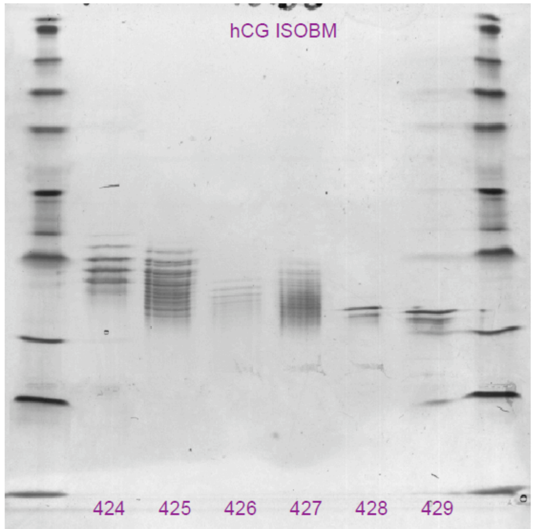
hCG ISOBM
424 425 426 427 428 429

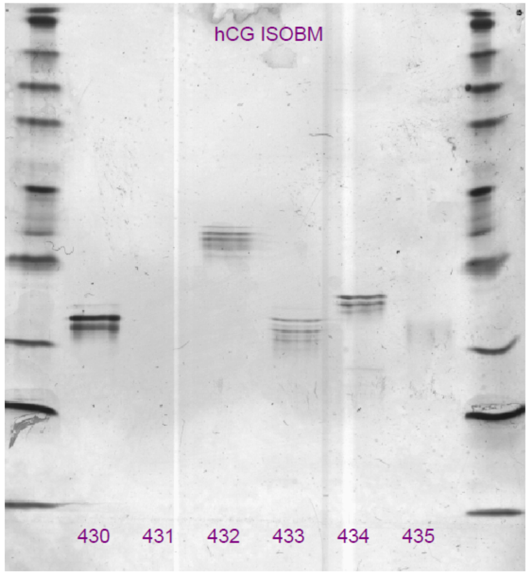
hCG ISOBM
430 431 432 433 434 435

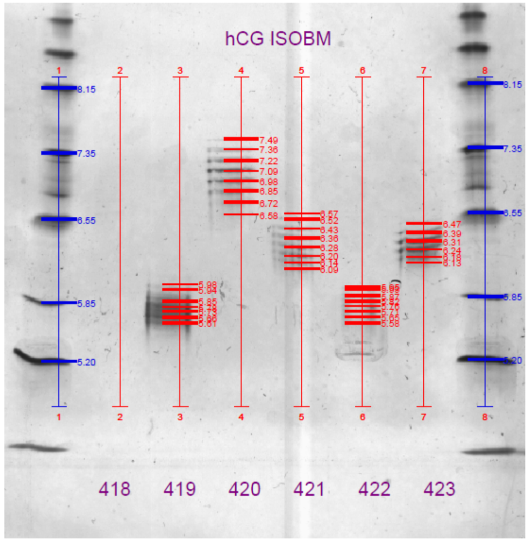
hCG ISOBM
418 419 420 421 422 423

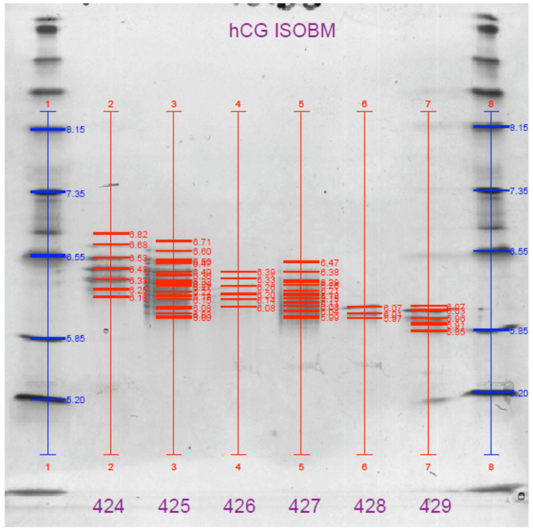
hCG ISOBM
424 425 426 427 428 429

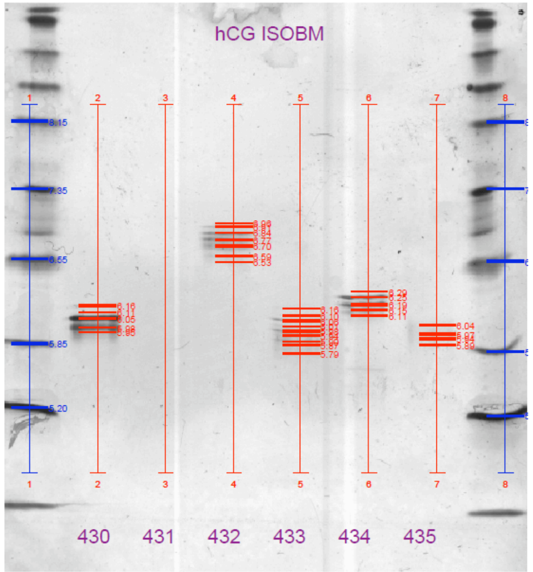
hCG ISOBM
430 431 432 433 434 435

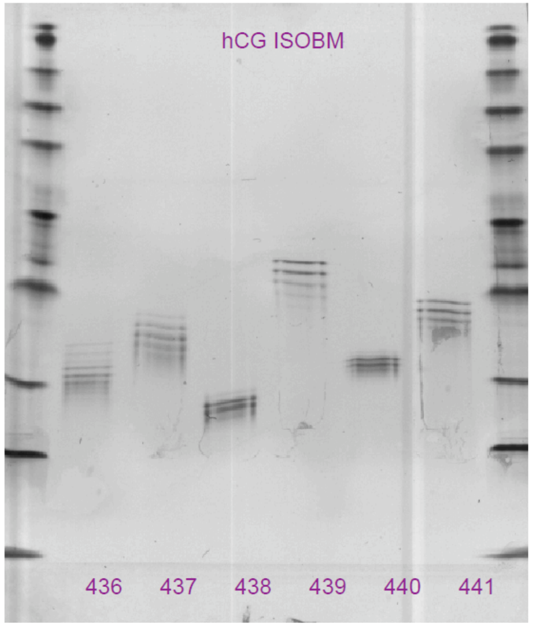
hCG ISOBM
436 437 438 439 440 441

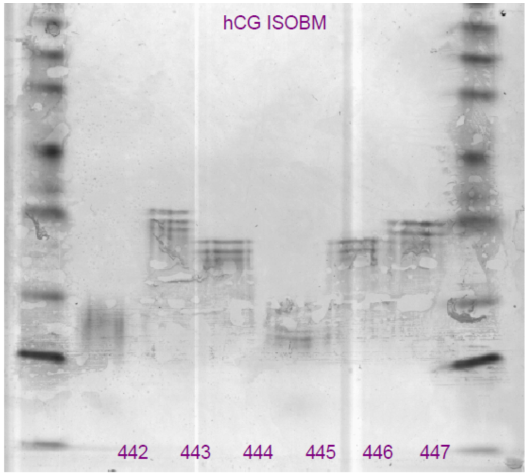
hCG ISOBM
442 443 444 445 446 447

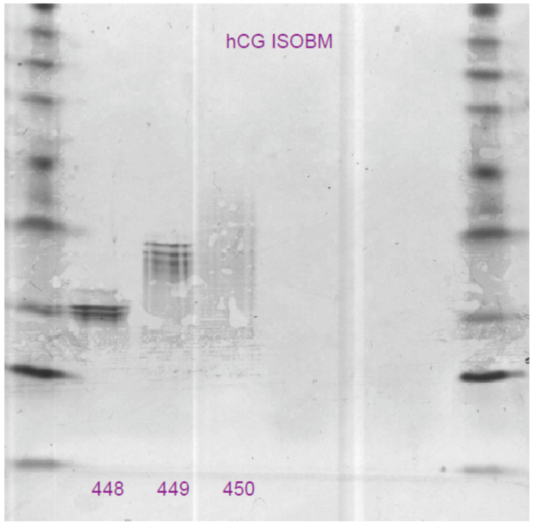
hCG ISOBM
448 449 450

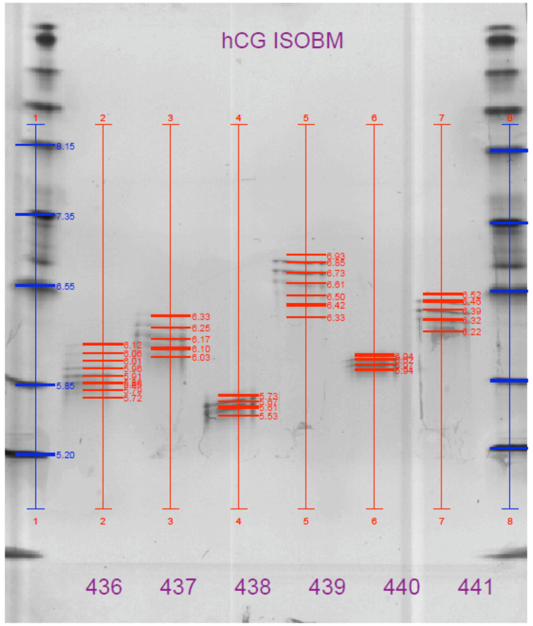
hCG ISOBM
436 437 438 439 440 441

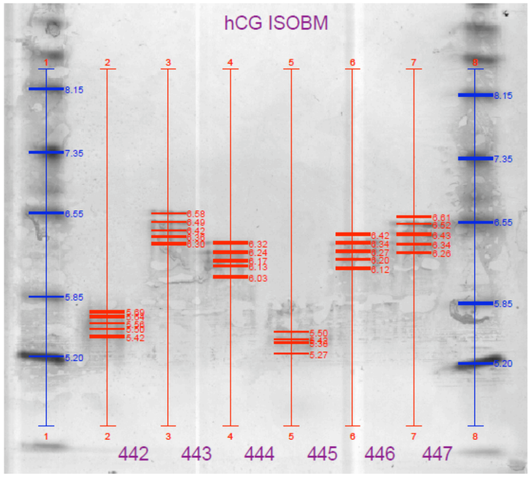
hCG ISOBM
442 443 444 445 446 447

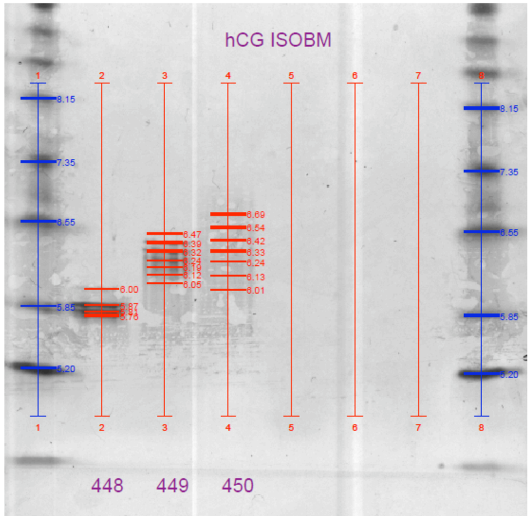
hCG ISOBM
448 449 450

Select samples
run in the basic position:

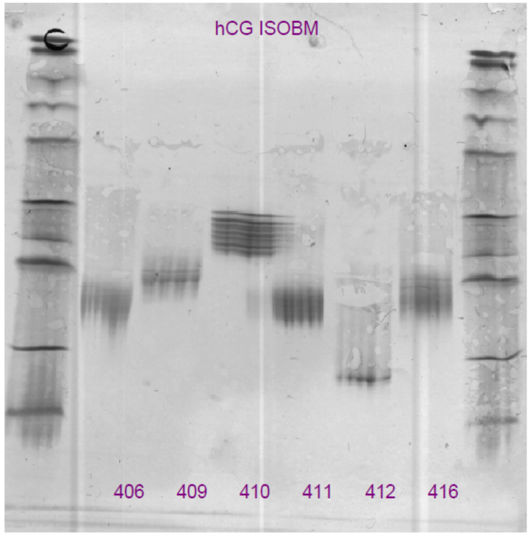

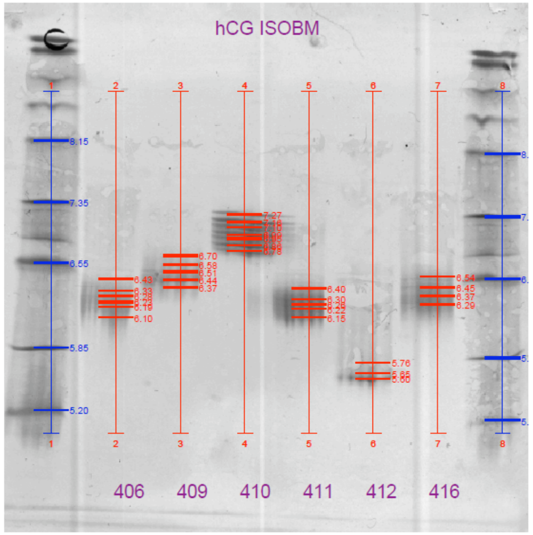

Online Resource 18

ISOBM-mAbs: MS analysis

Sample list for MS analysis

Sample ID	SDS-PAGE Reduced Testing*
382	Double light chain
385	Double heavy chain
388	Double light Chain
400	Double light chain
406	Double light chain
420	Highest purity
426	Double heavy chain
450	Double light chain

* SDS-PAGE results obtained by L. Harwick, D-09A1

MS analysis of hCG Ab 382 (TCEP reduced)

MALDI-TOF-MS

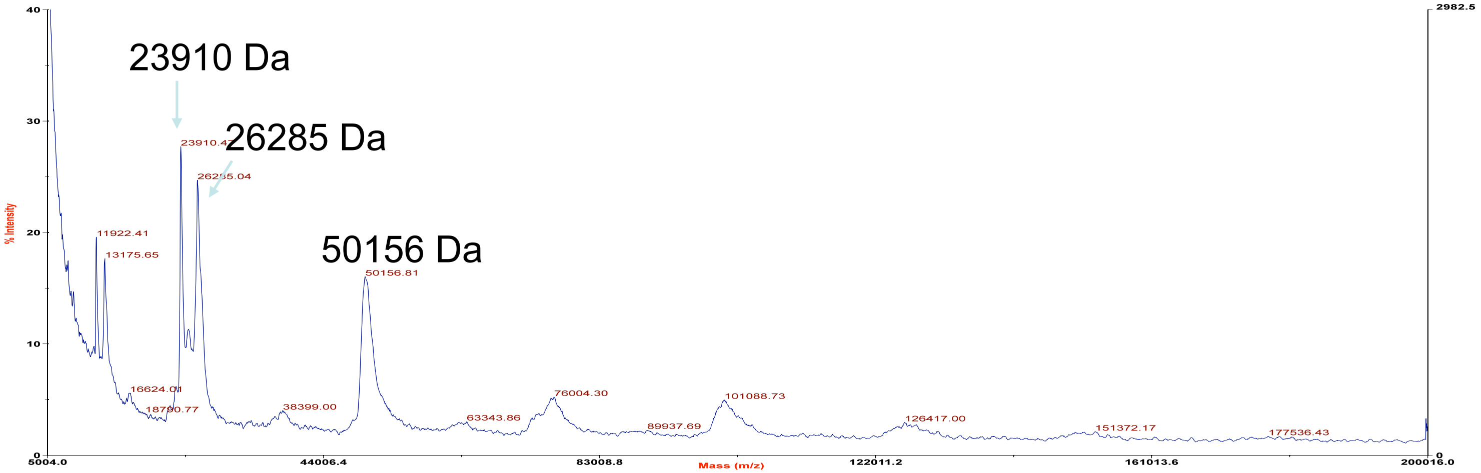

ESI-MS

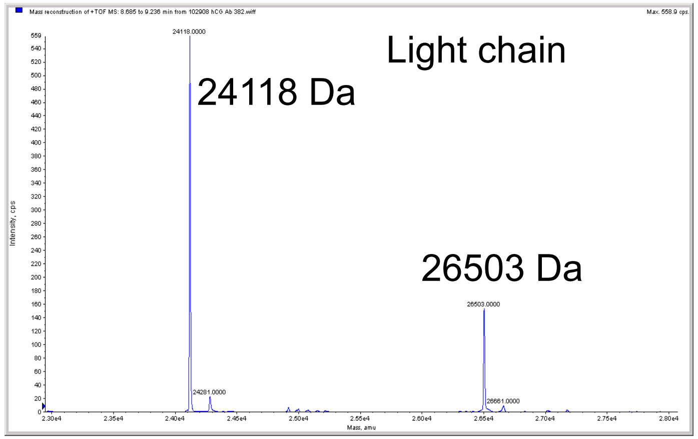

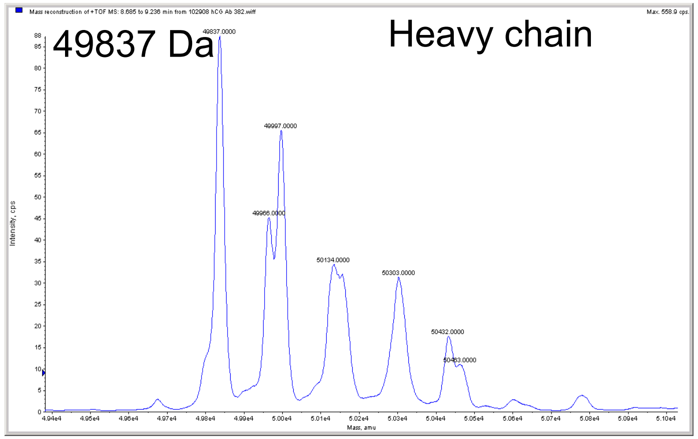

ESI-MS analysis of hCG Ab 382 (deglycosylated)

Light chain | Heavy chain

N-degly

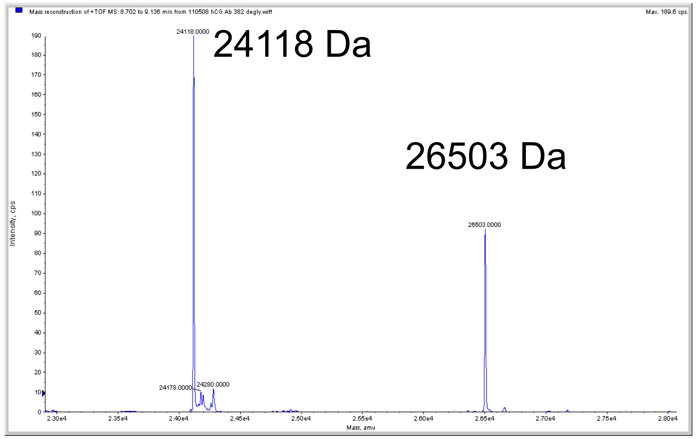

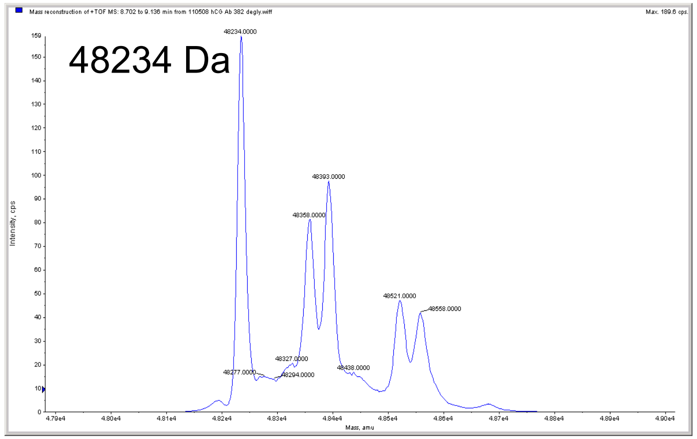

N&O-degly

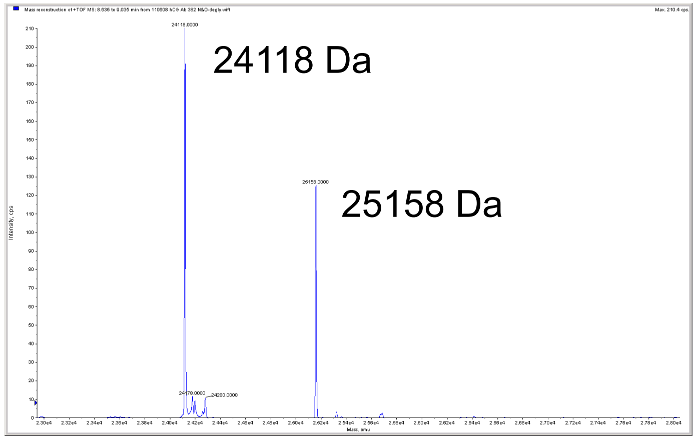

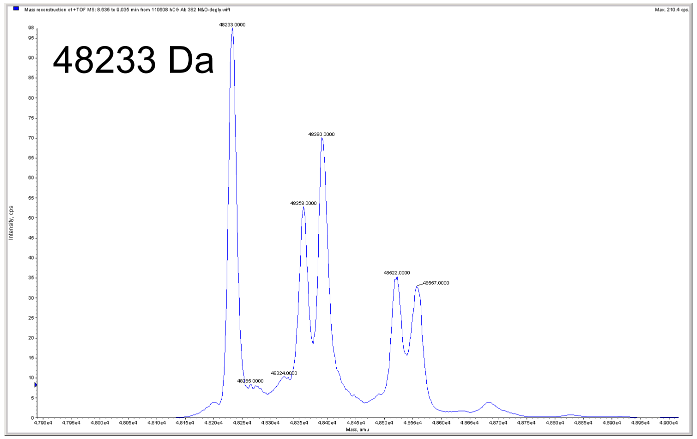

MS analysis of hCG Ab 385 (TCEP reduced)

MALDI-TOF-MS

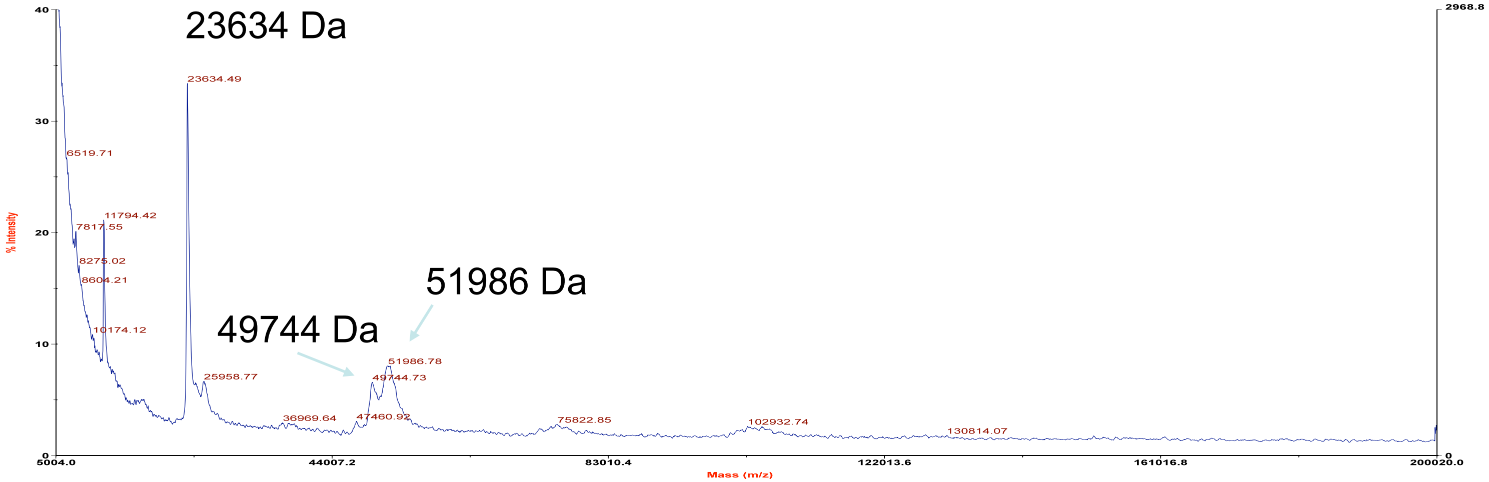

ESI-MS

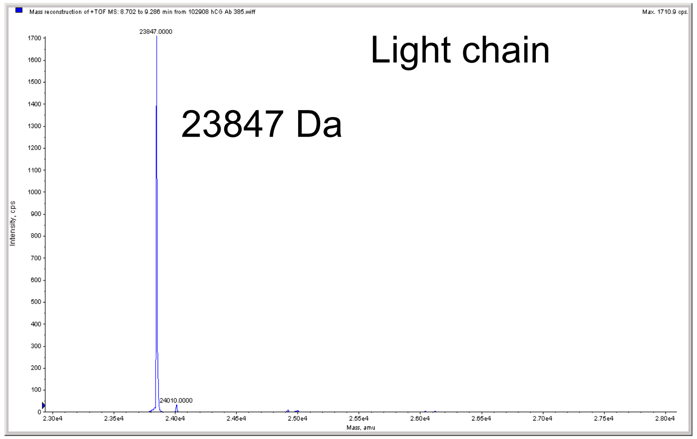

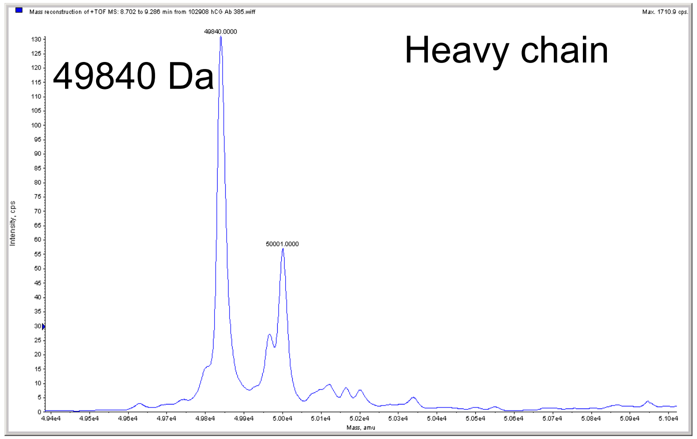

ESI-MS analysis of hCG Ab 385 (deglycosylated)

Light chain

Heavy chain

N-degly

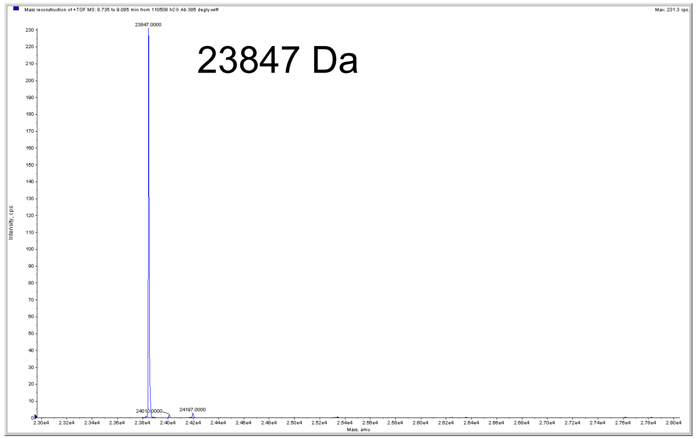

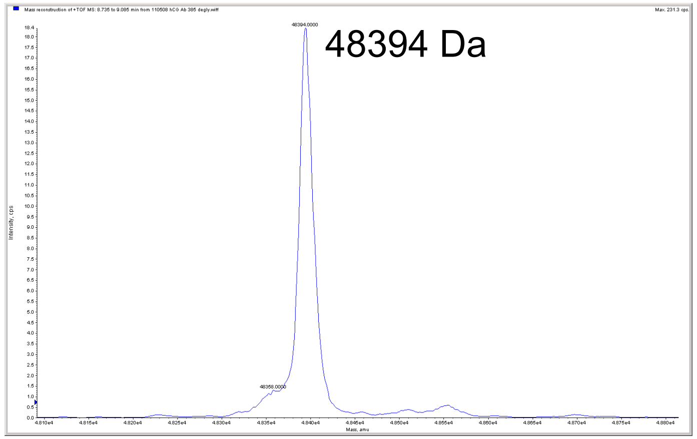

N&O-degly

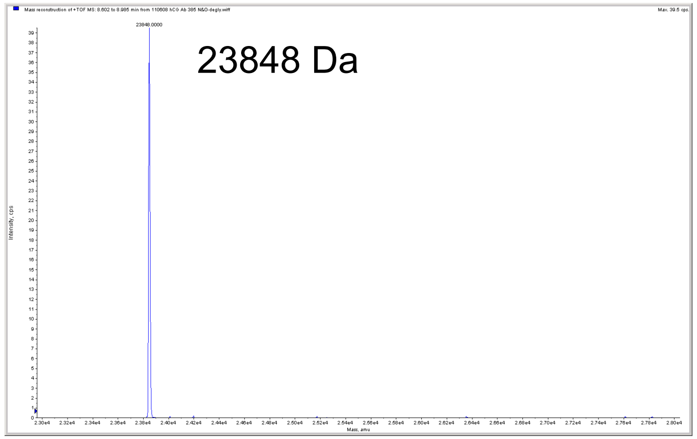

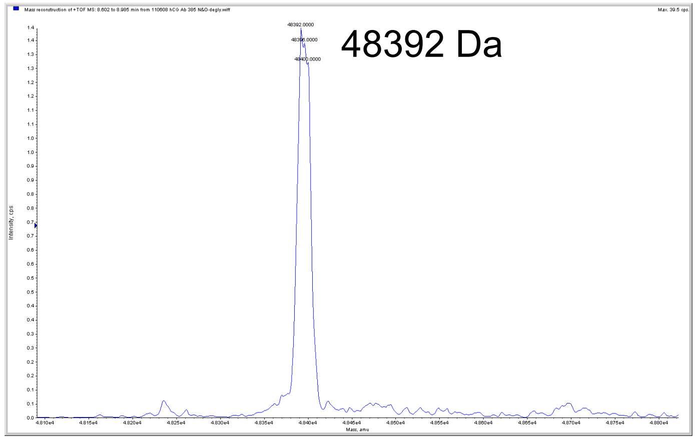

MS analysis of hCG Ab 388 (TCEP reduced)

MALDI-TOF-MS

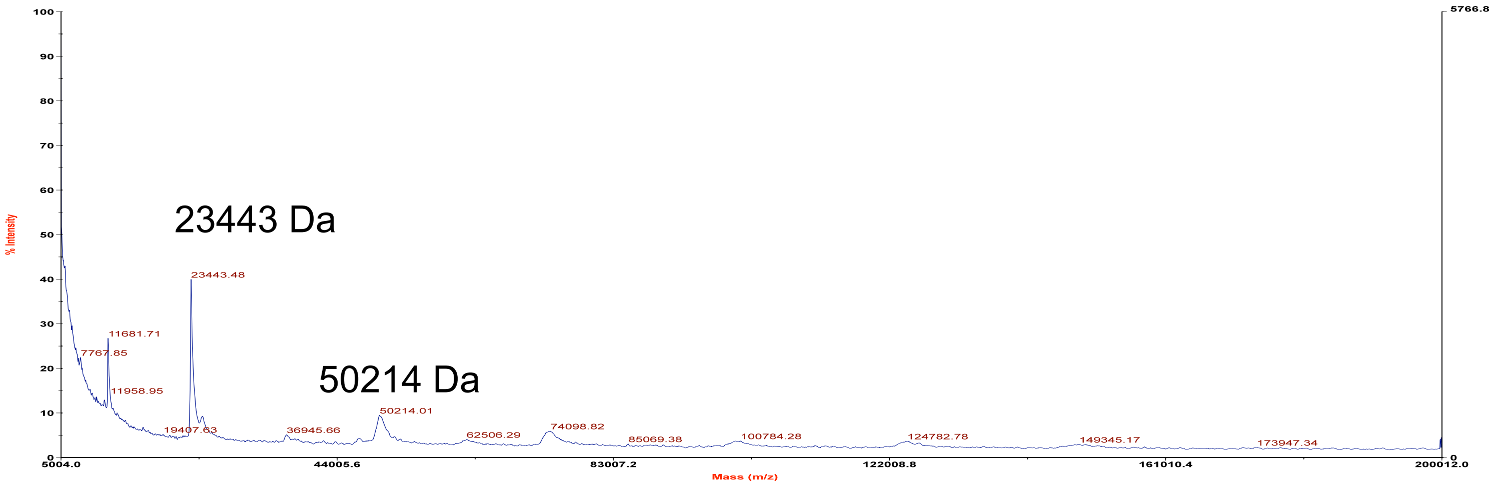

ESI-MS

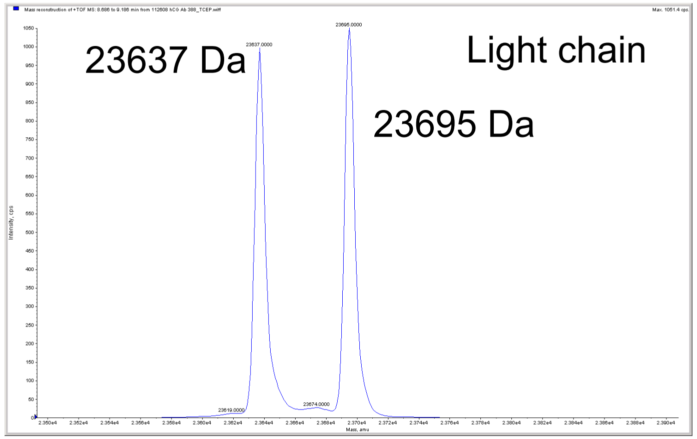

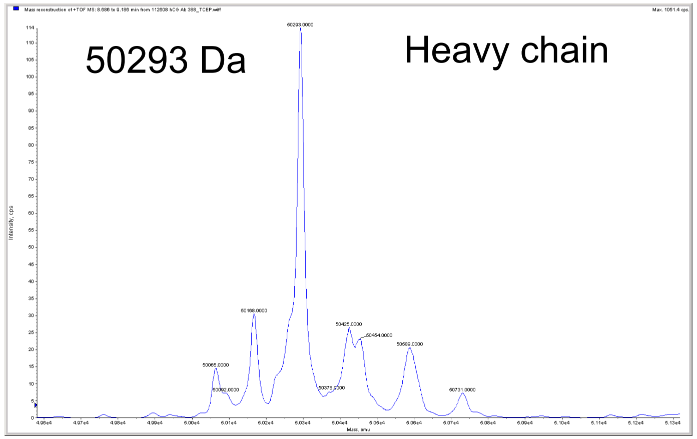

ESI-MS analysis of hCG Ab 388 (deglycosylated)

Light chain | Heavy chain

N-degly

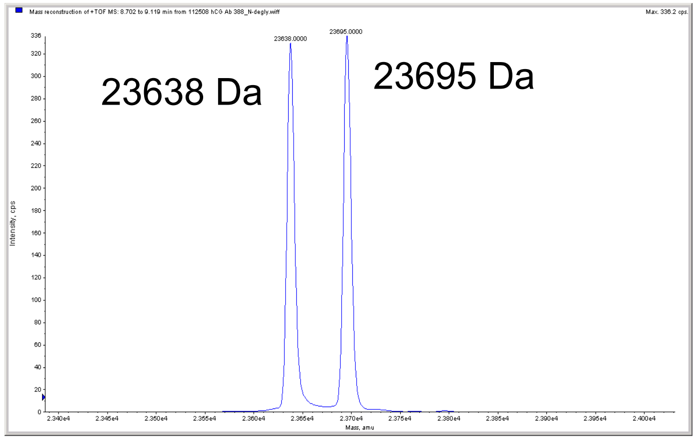

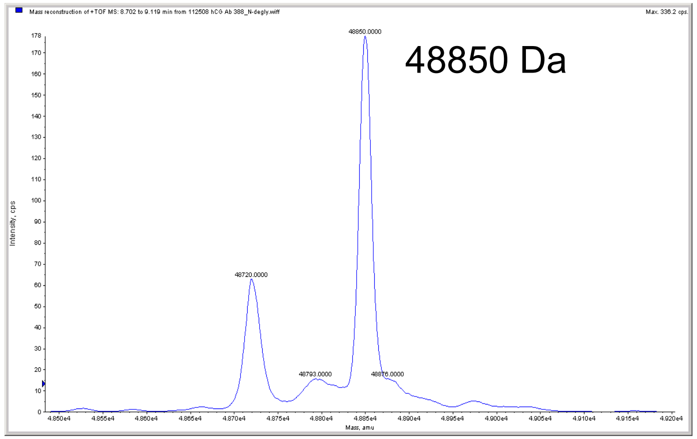

N&O-degly

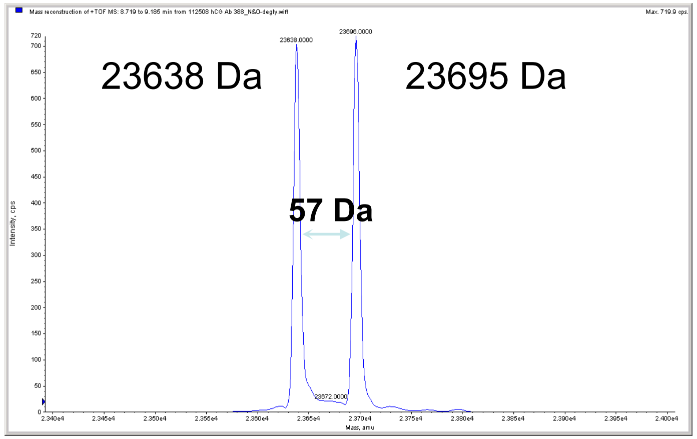

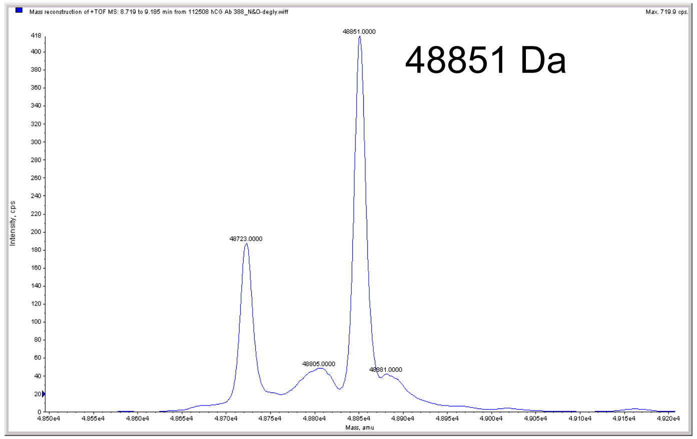

MS analysis of hCG Ab 400 (TCEP reduced)

MALDI-TOF-MS

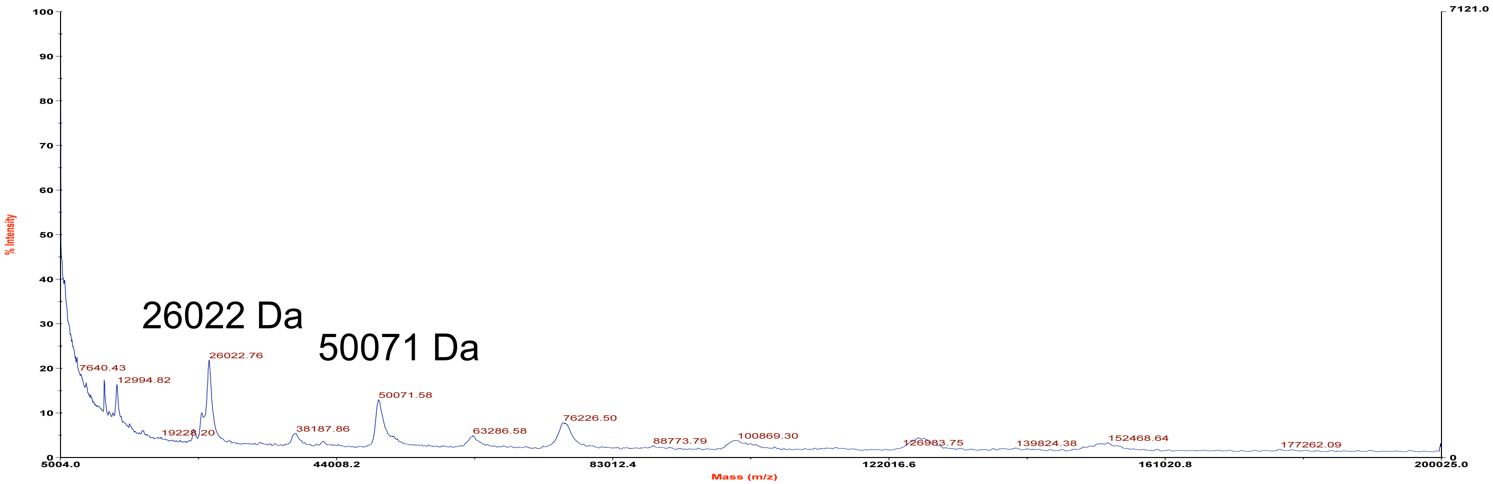

ESI-MS

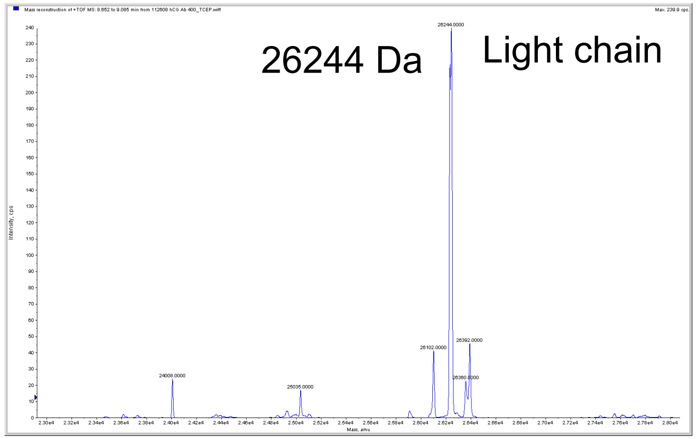

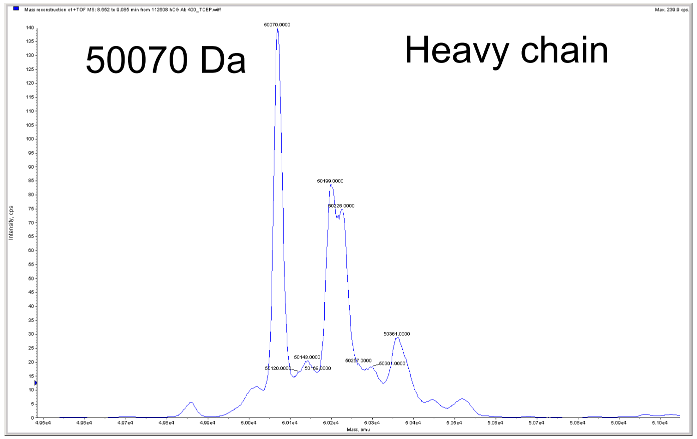

ESI-MS analysis of hCG Ab 400 (deglycosylated)

Light chain

Heavy chain

N-degly

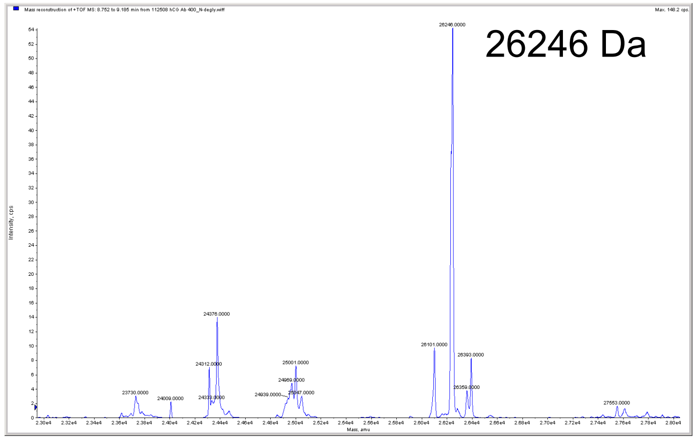

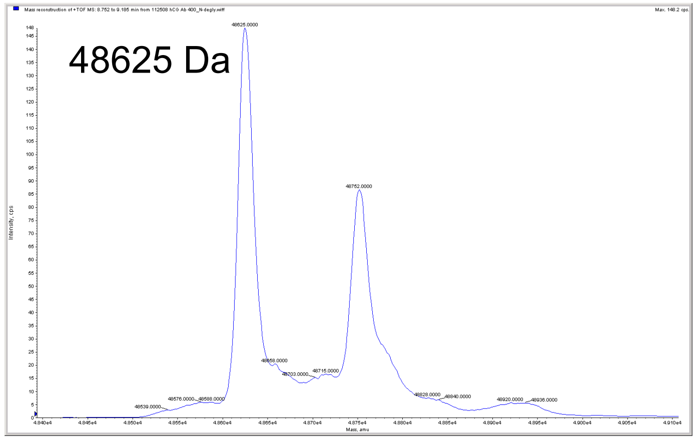

N&O-degly

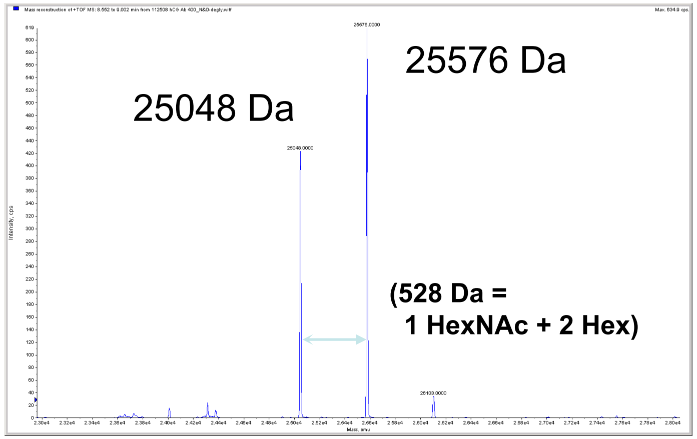

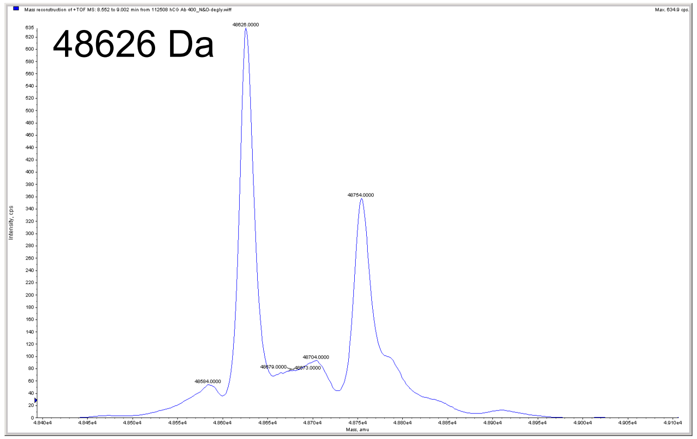

MS analysis of hCG Ab 406 (TCEP reduced)

MALDI-TOF-MS

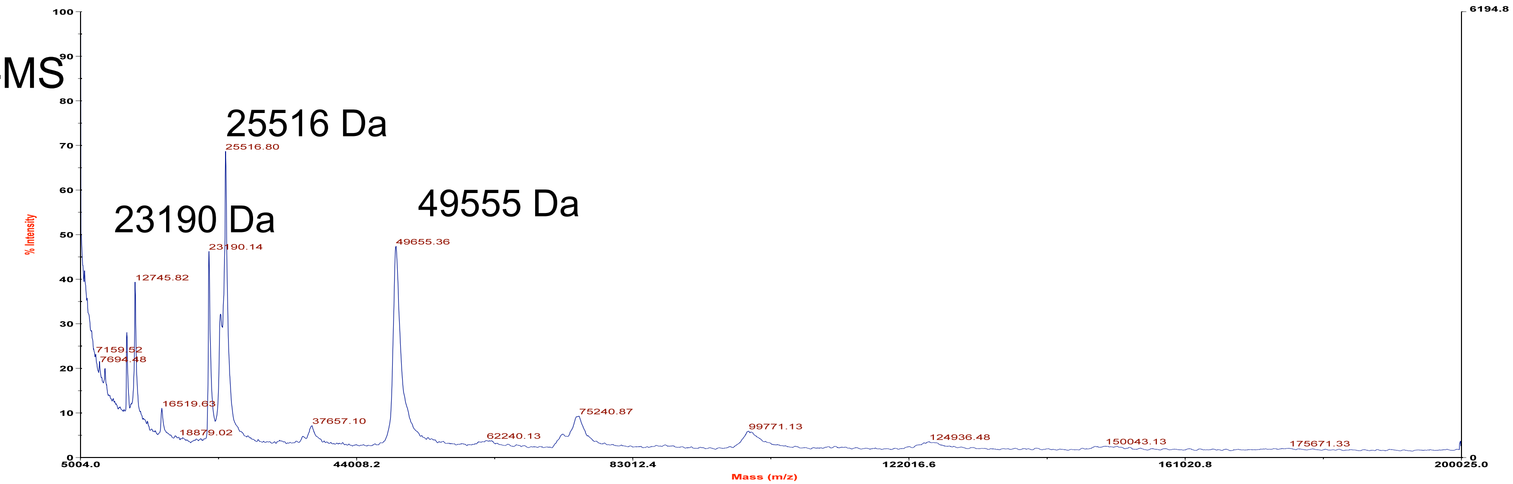

ESI-MS

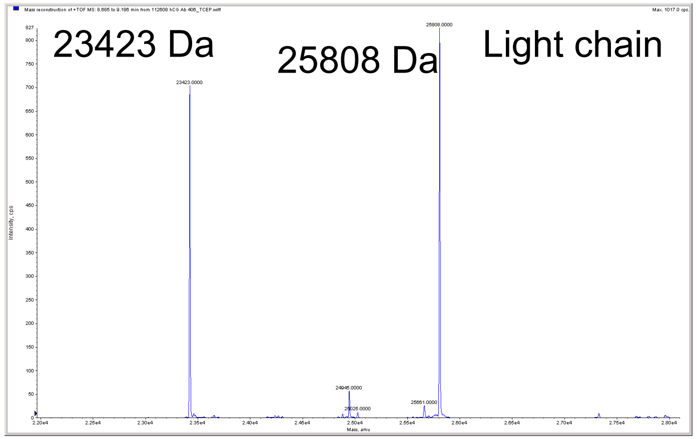

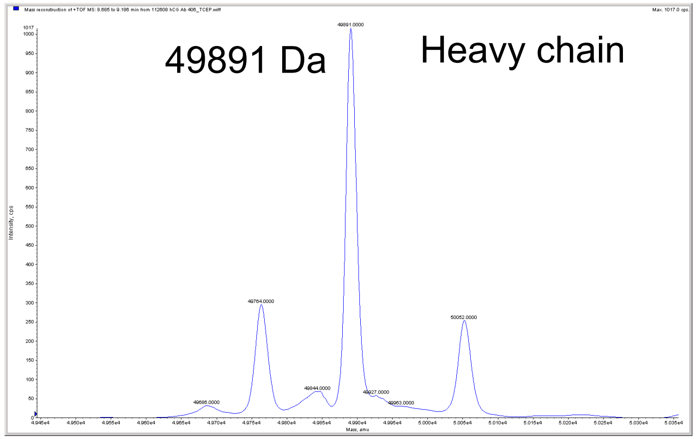

ESI-MS analysis of hCG Ab 406 (deglycosylated)

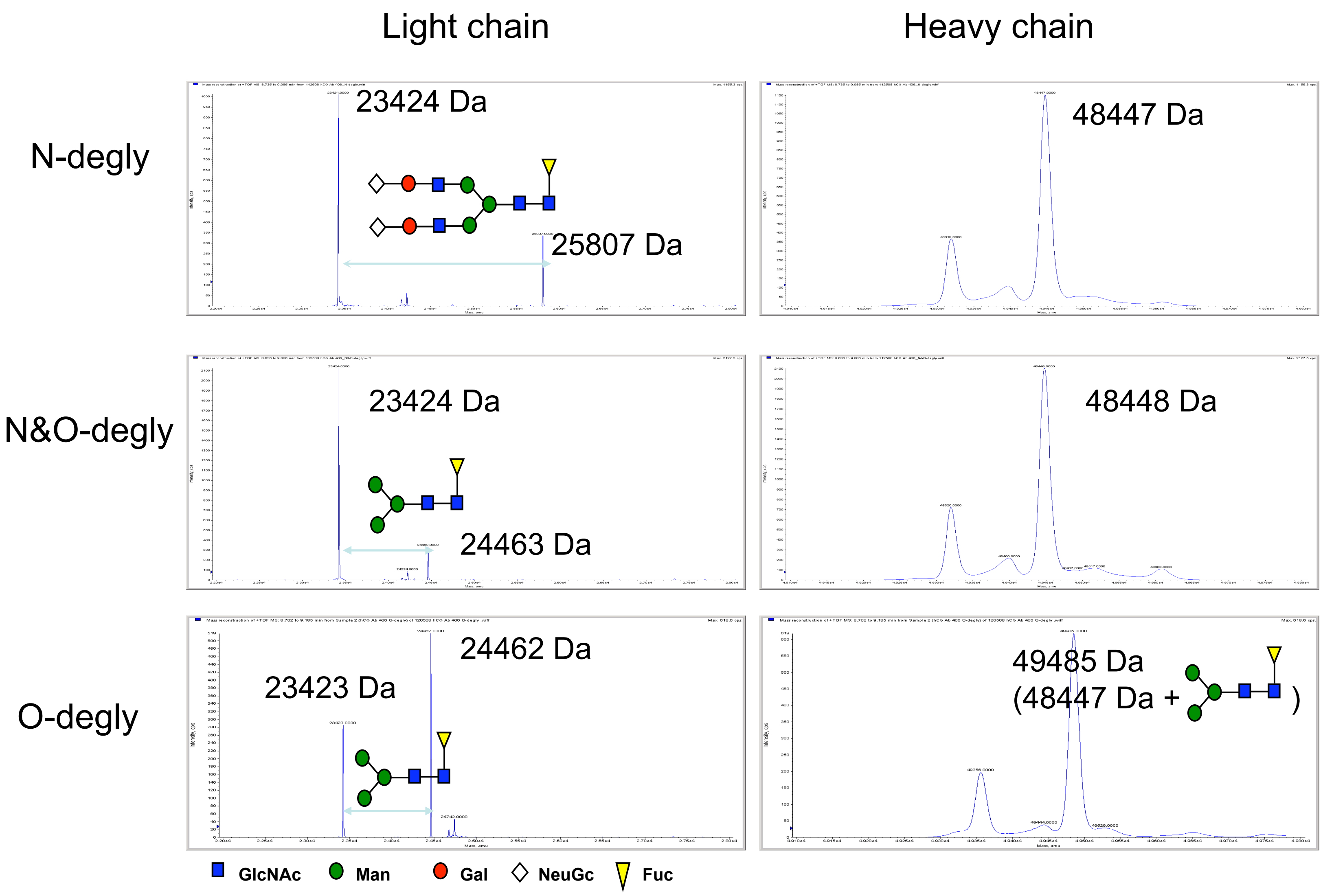

MS analysis of hCG Ab 420 (TCEP reduced)

MALDI-TOF-MS

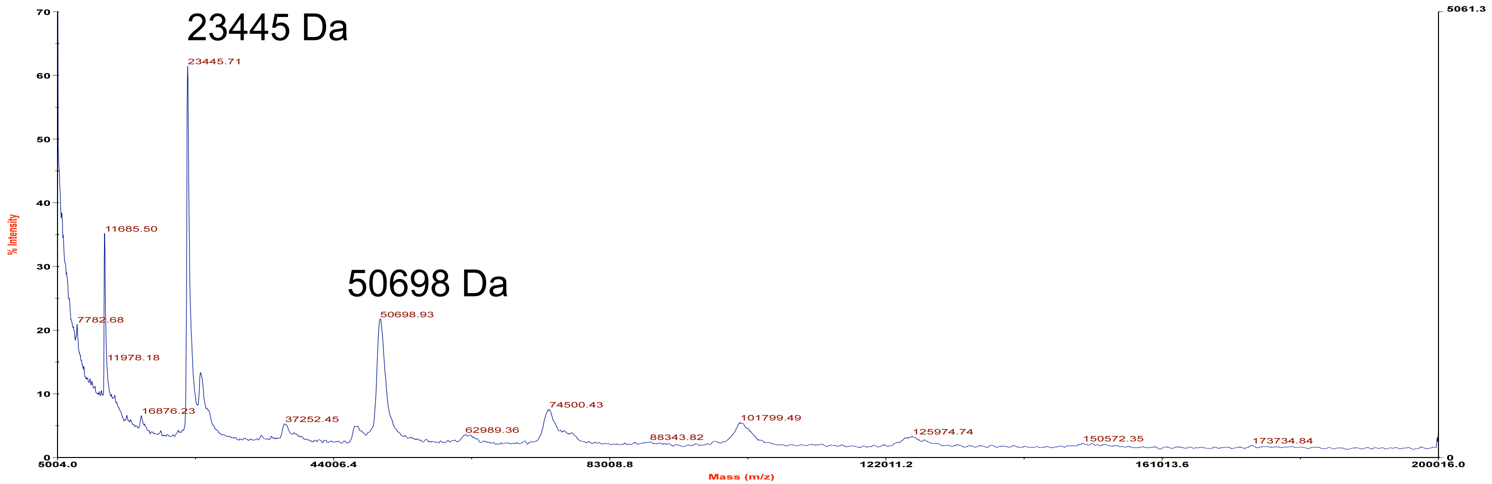

ESI-MS

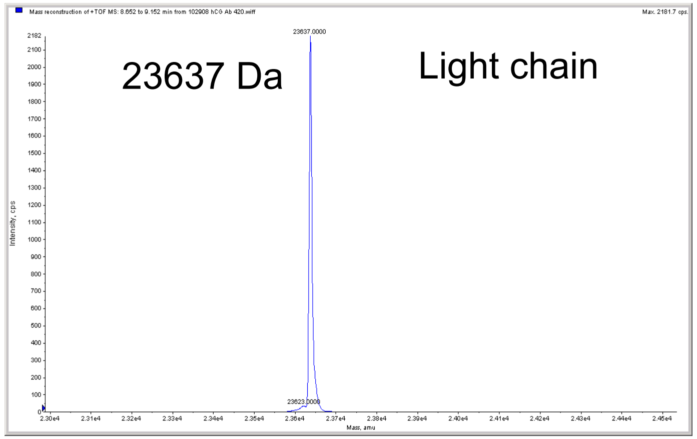

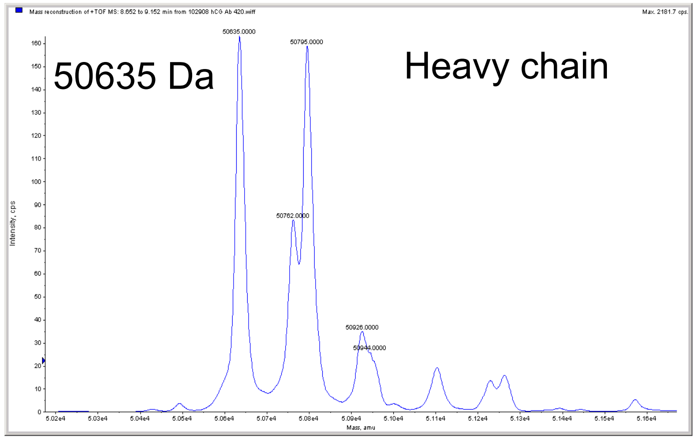

ESI-MS analysis of hCG Ab 420 (deglycosylated)

Light chain | Heavy chain

N-degly

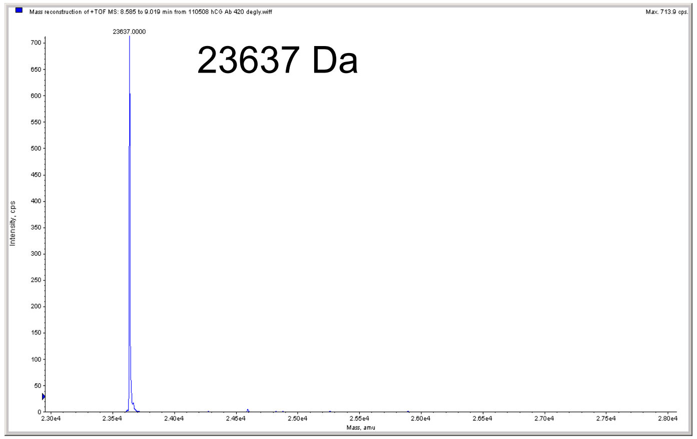

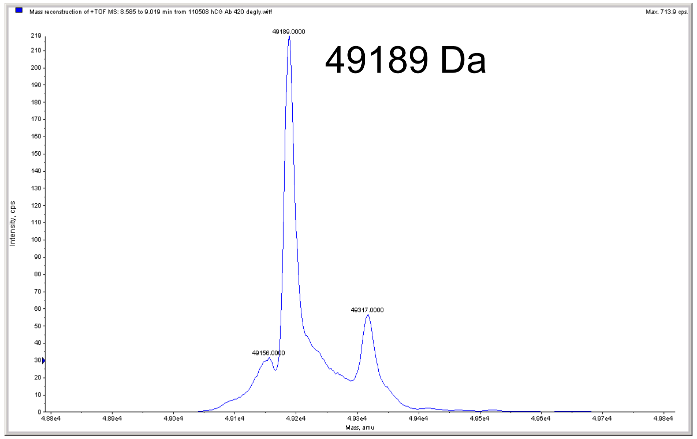

N&O-degly

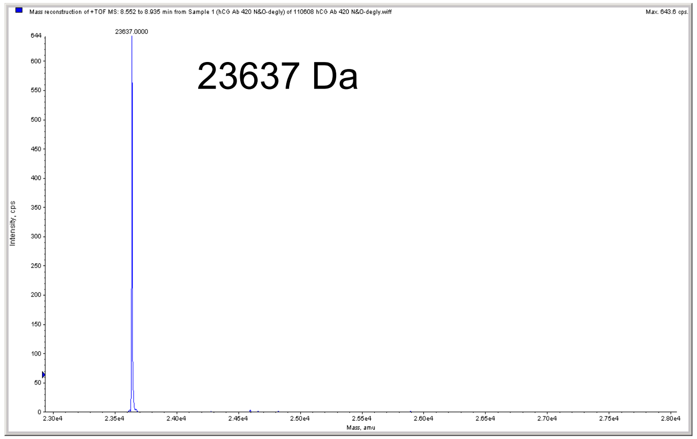

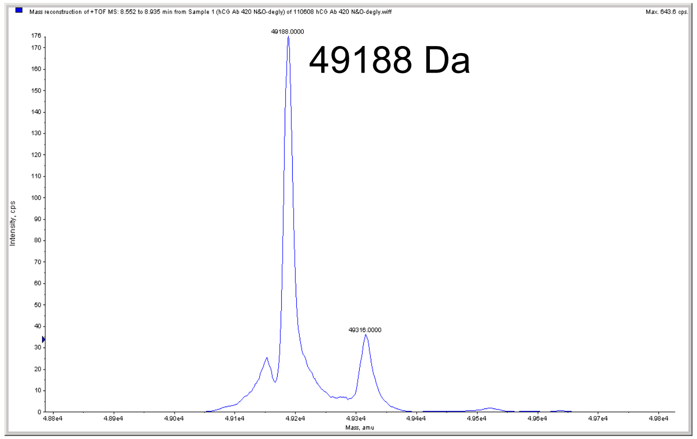

MS analysis of hCG Ab 426 (TCEP reduced)

MALDI-TOF-MS

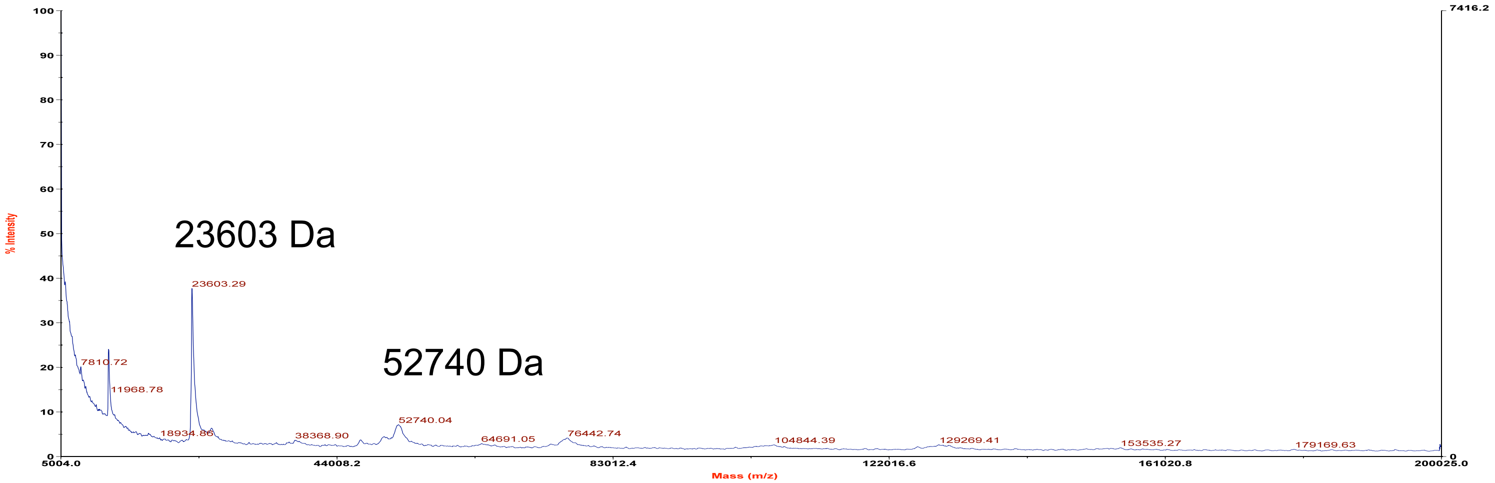

ESI-MS

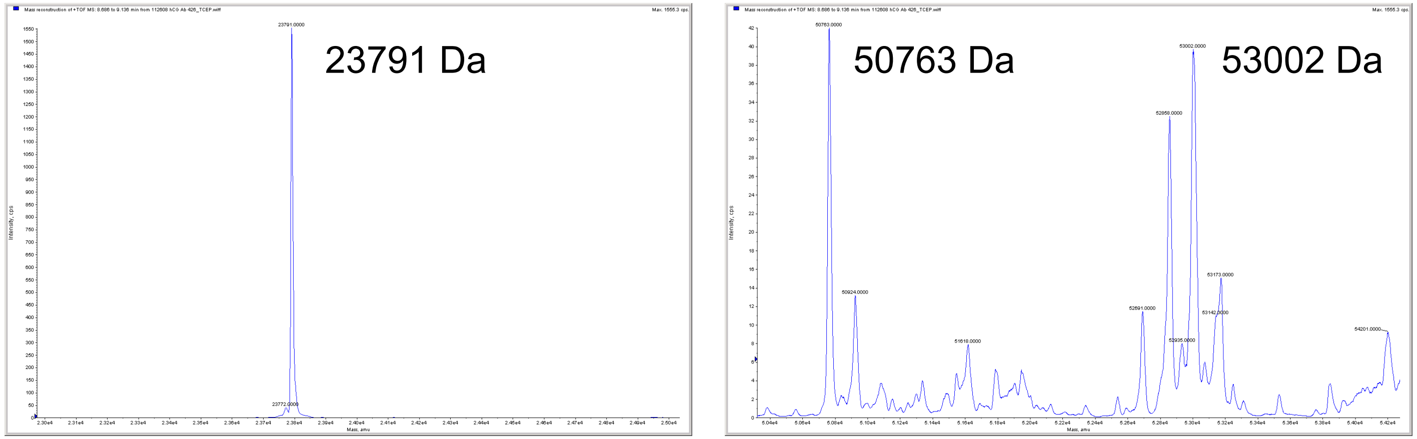

ESI-MS analysis of hCG Ab 426 (deglycosylated)

Light chain

Heavy chain

N-degly

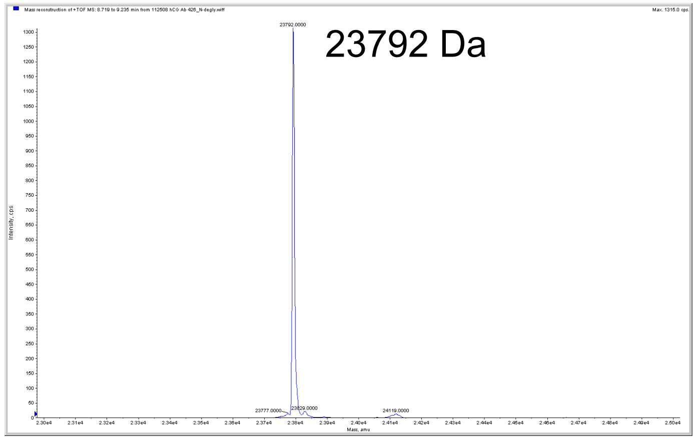

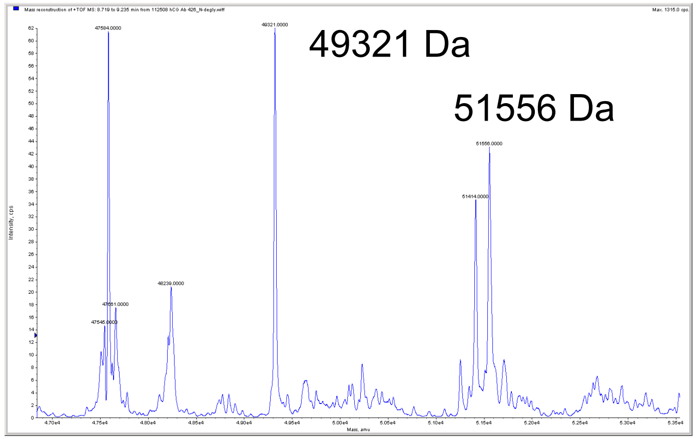

N&O-degly

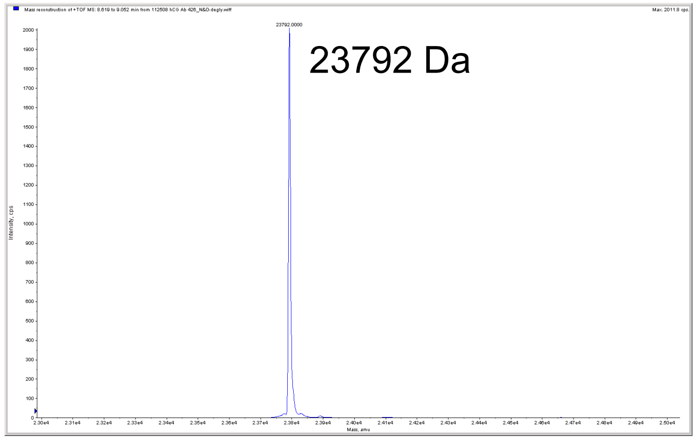

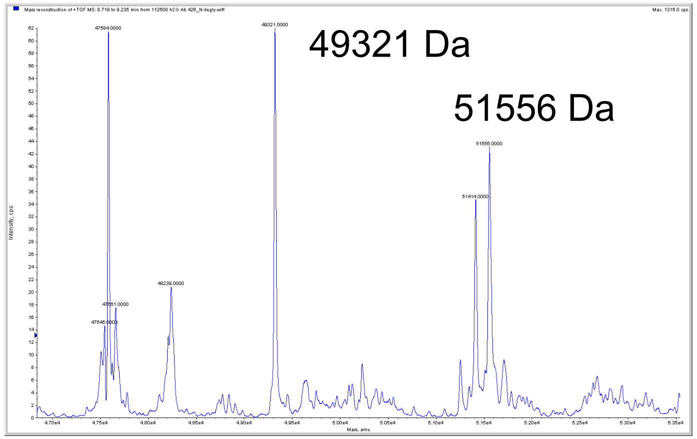

MS analysis of hCG Ab 450 (TCEP reduced)

MALDI-TOF-MS

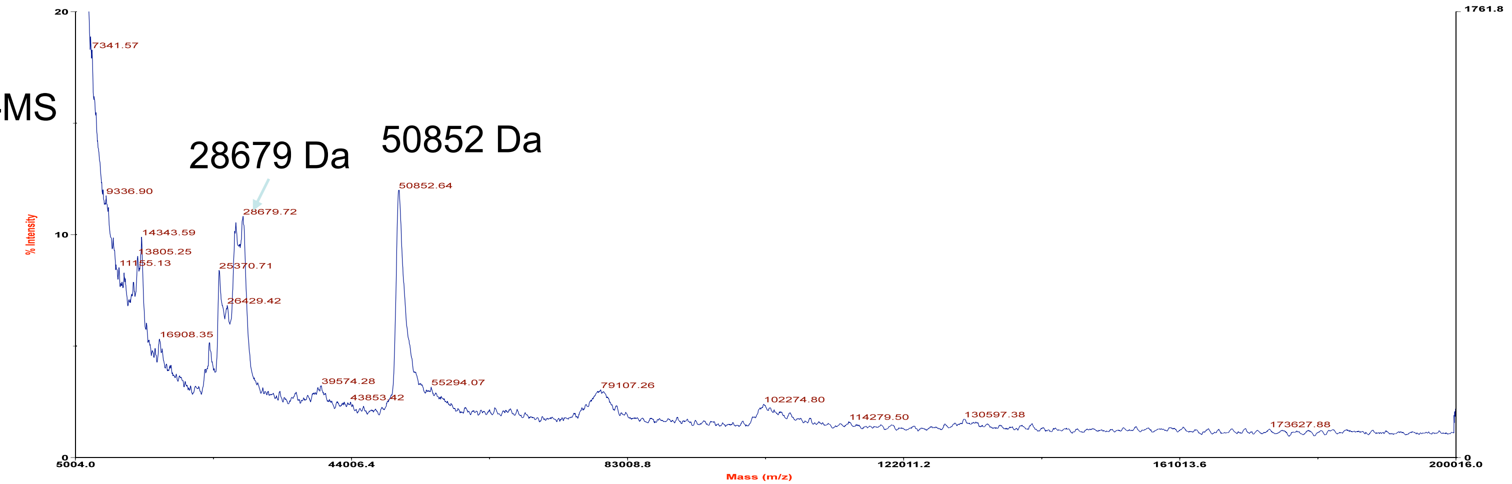

ESI-MS

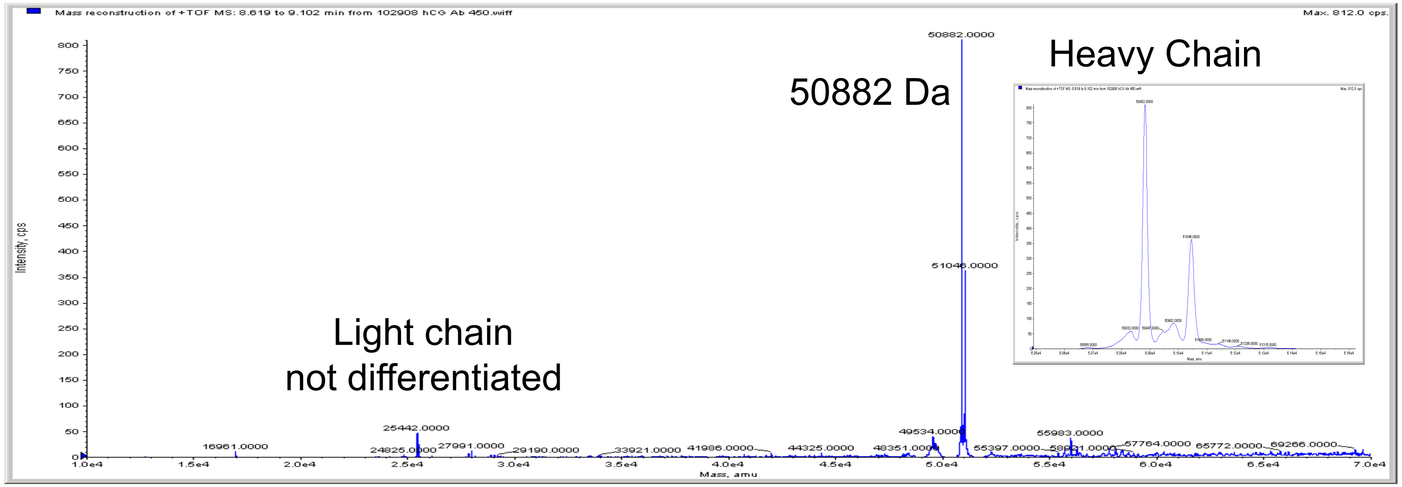

ESI-MS analysis of hCG Ab 450 (deglycosylated)

N-degly

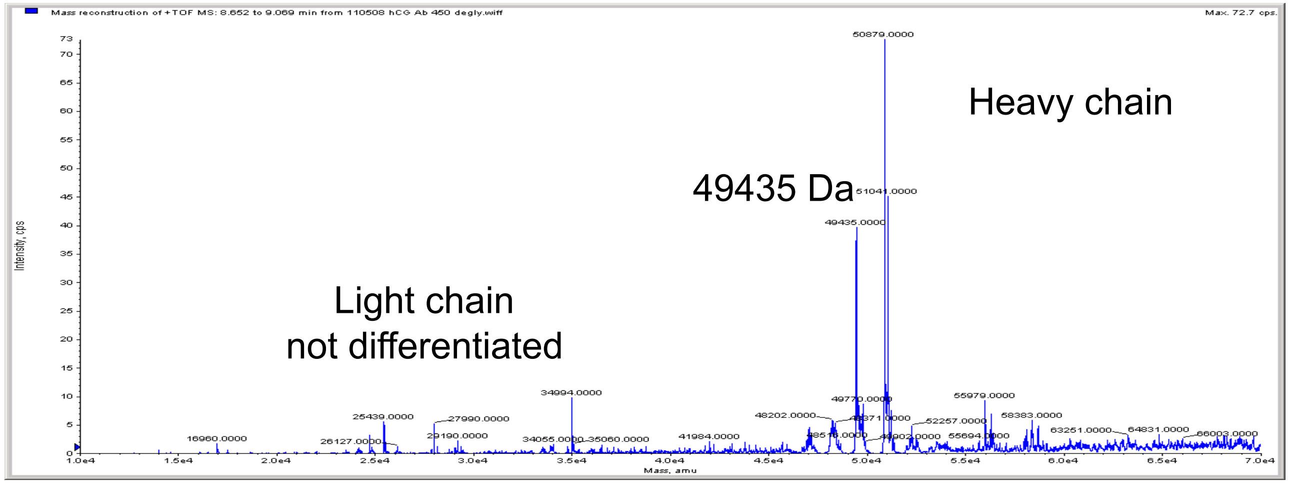

N&O-degly

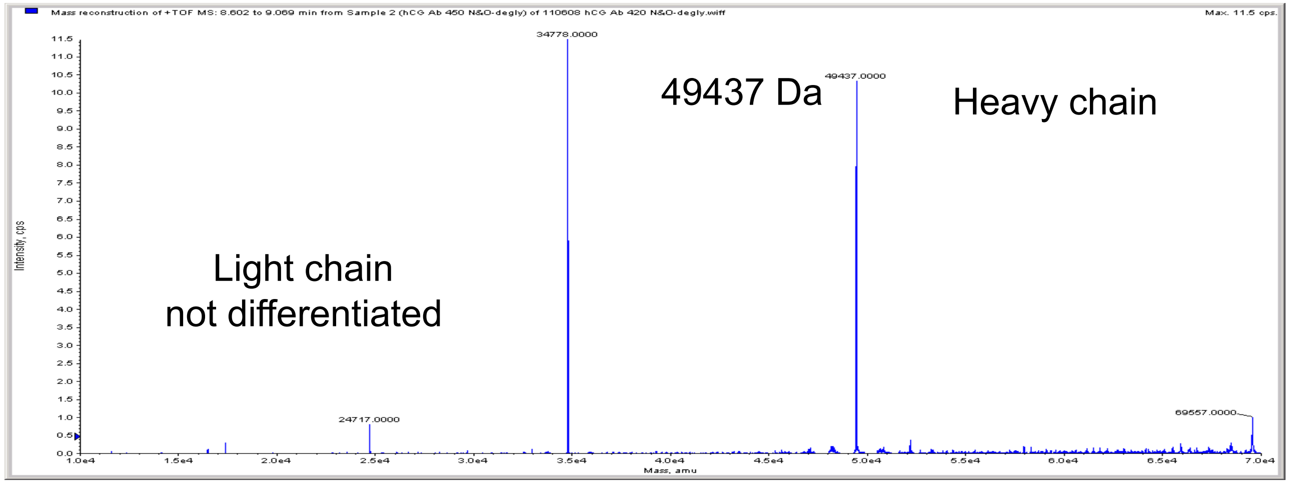

Summary of MS analysis of hCG ISOBM Ab samples

MS analysis summary:

Sample 382: Two light chains, one of the light chains is O-glycosylated; more than one group of heavy chains after deglycosylation, suggesting two antibodies may be present in sample

Sample 385: One light chain; two groups of heavy chains due to glycosylation

Sample 388: Two non-glycosylated light chains with MW difference of ~57 Da (may be due to Glycine amino acid residue difference); one group of heavy chains

Sample 400: Multiple light chain peaks, light chains are O-glycosylated, after deglycosylation there are two light chains with MW difference of 528 Da (possibly due to trisaccharide difference); one group of heavy chains

Sample 406: Light chains are partially N-glycosylated; one group of heavy chains

Sample 420: One light chain; one group of heavy chains, tested as a control sample

Sample 426: One light chain; heavy chains complex with at least two groups of heavy chains

Sample 450: Complex light chains by MALDI-TOF-MS, light chain not differentiated by ESI-MS; one group of heavy chains

Online Resource 19

ISOBM-mAbs: Competitive RIA with hCGβ109-145 vs. hCGβ*. Determination of % cross-reactivity.

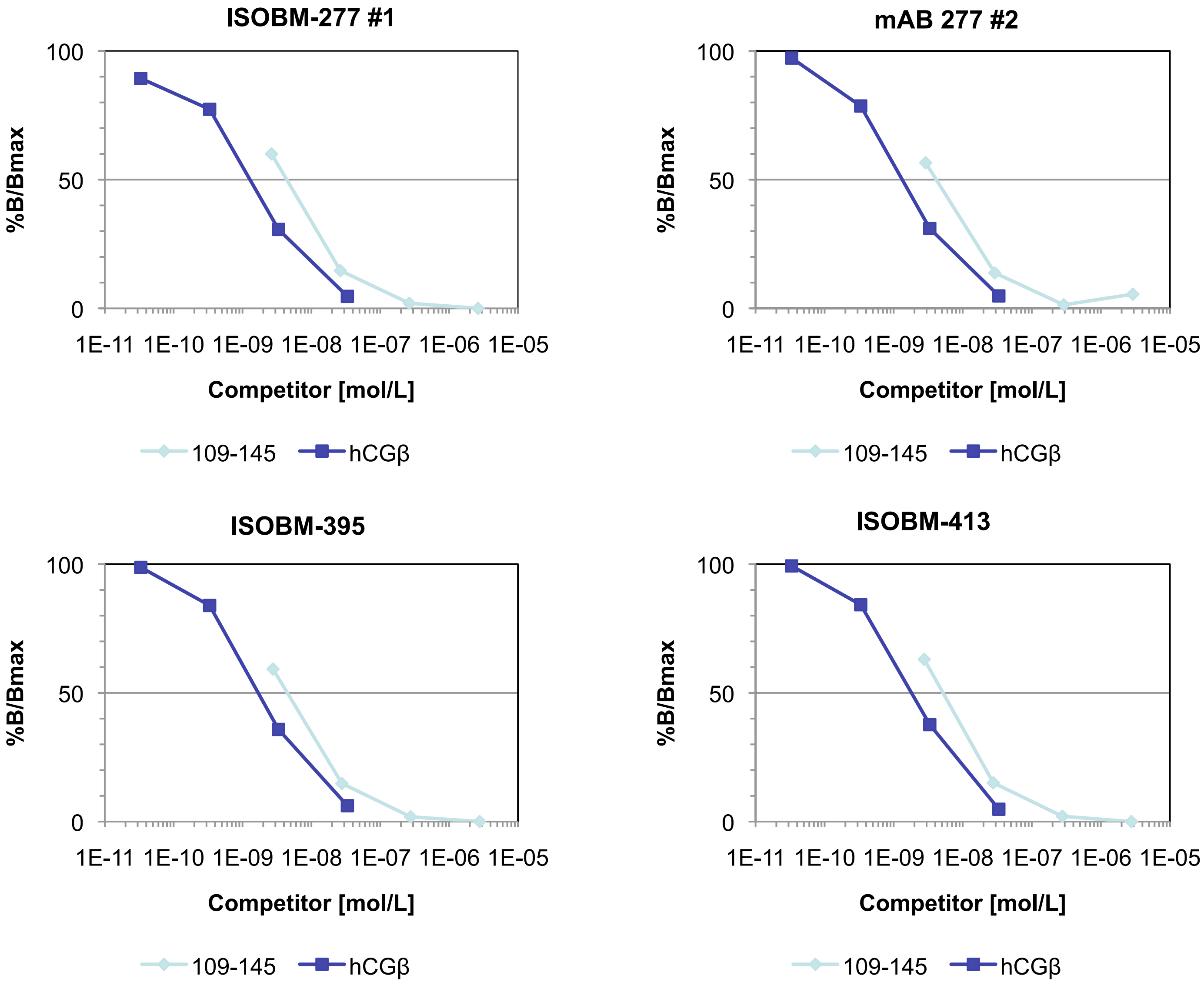
ISOBM-277 #1
mAB 277 #2
ISOBM-395
ISOBM-413
100
50
0
%B/Bmax
1E-11 1E-10 1E-09 1E-08 1E-07 1E-06 1E-05
Competitor [mol/L]
109-145
hCGβ

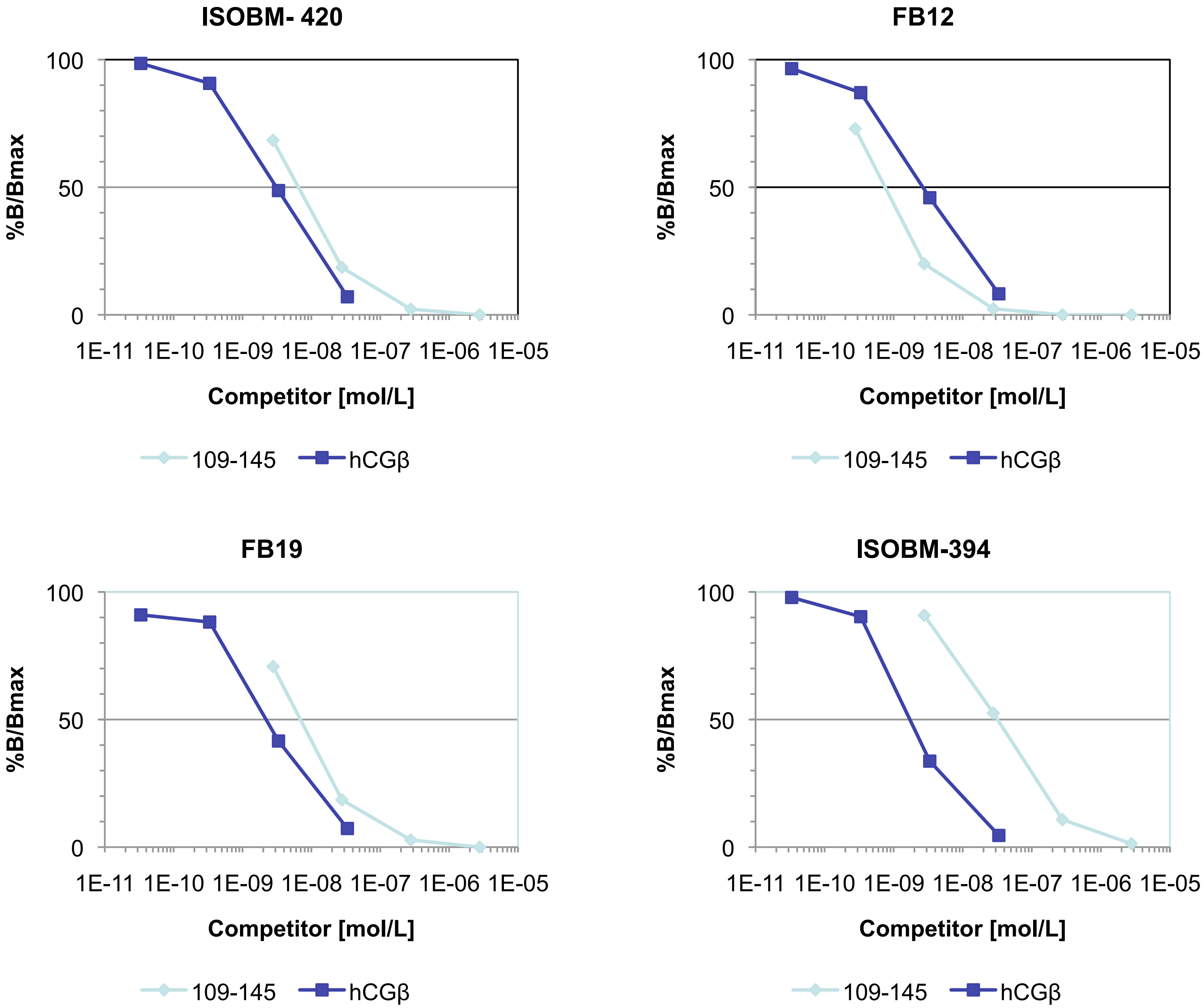
ISOBM- 420
FB12
FB19
ISOBM-394
%B/Bmax
100
50
0
1E-11 1E-10 1E-09 1E-08 1E-07 1E-06 1E-05
Competitor [mol/L]
109-145
hCGβ

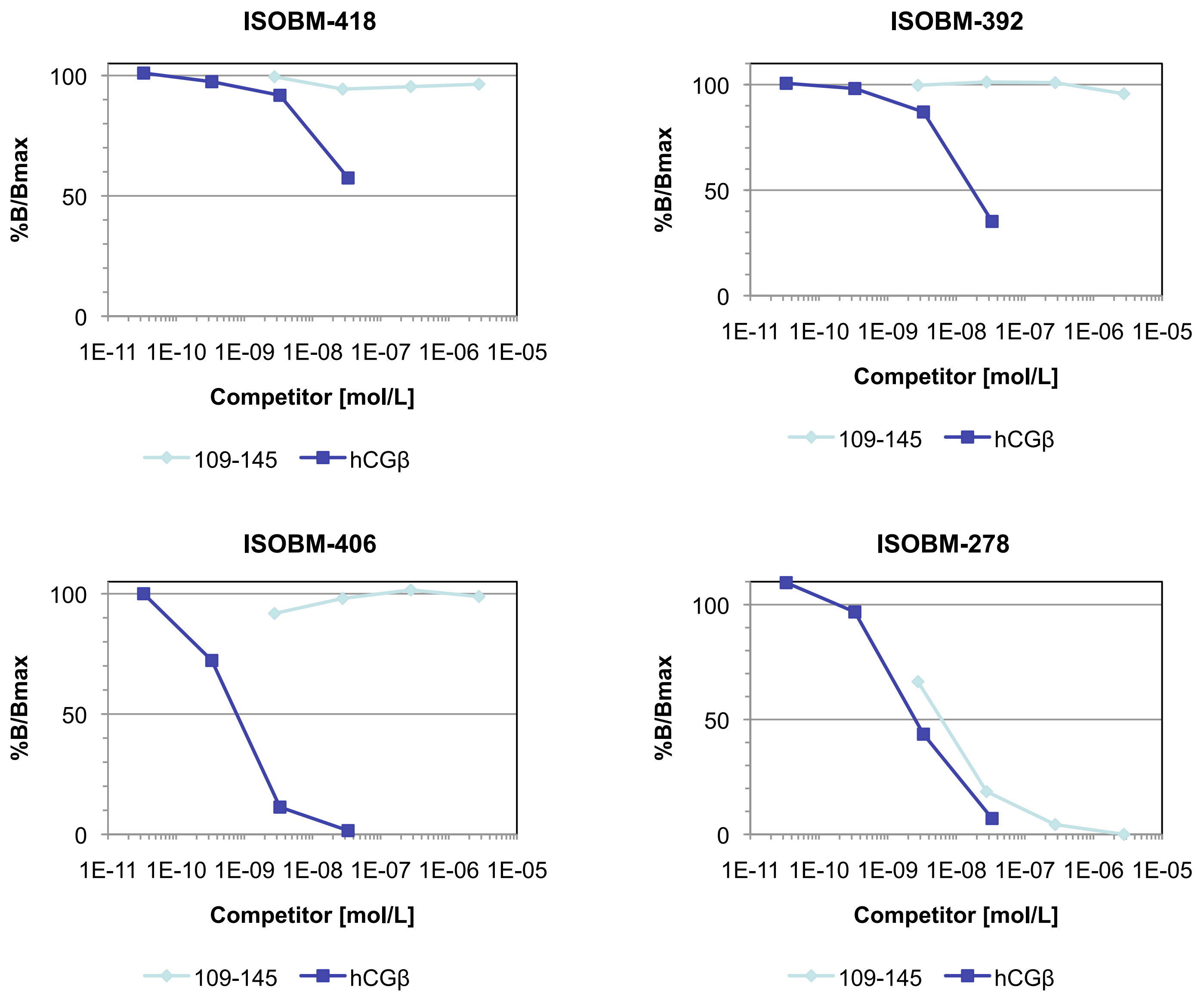

ISOBM-418
ISOBM-392
ISOBM-406
ISOBM-278
%B/Bmax
100
50
0
1E-11 1E-10 1E-09 1E-08 1E-07 1E-06 1E-05
Competitor [mol/L]
109-145
hCGβ

Online Resource 20

ISOBM-mAbs: Competitive Ligand Assay (left panel) and BIAcore® analysis of Ab specificities and affinities.

Minor cross-reactions of c-mAbs (ISOBM-424 and 389) with hCGβ as seen in BIAcore® have also been observed previously (PB unpublished data) pointing to a contribution of assembled hCGβ to c-epitopes.

Gray boxes … positive competition
White boxes … no competition
n.a. … not applicable

Specificity (CLA)								
**ISOBMii Ab Codes**	**hCG**	**hCGβ**	**hCGn**	**hCGβn**	**hCGβcf**	**hCGα**	**hLH**	**Epitope** CLA
403								β1,2,4
435*								β1,2,4
382								β1,2,4
388								β1,2,4
390								β1,2,4
402								β1,2,4
408	n/a	n/a	n/a	n/a	n/a	n/a	n/a	-
416								β1,2,4
417								β1,2,4
426								β1,2,4
427								β1,2,4
449*								β1,2,4
396	n/a	n/a	n/a	n/a	n/a	n/a	n/a	-
399								β3,5
400	n/a	n/a	n/a	n/a	n/a	n/a	n/a	-
401	n/a	n/a	n/a	n/a	n/a	n/a	n/a	-
405								β3,5
423								β3,5
434*	n/a	n/a	n/a	n/a	n/a	n/a	n/a	-
441*	n/a	n/a	n/a	n/a	n/a	n/a	n/a	-
419								β1,2,4
445*								β1,2,4
428 sheep								β3,5
429 sheep								β1,2,4
430 sheep								β3,5
431 sheep								β3,5
442*								β3,5
410	n/a	n/a	n/a	n/a	n/a	n/a	n/a	-
437*	n/a	n/a	n/a	n/a	n/a	n/a	n/a	-
386	n/a	n/a	n/a	n/a	n/a	n/a	n/a	-
397	n/a	n/a	n/a	n/a	n/a	n/a	n/a	-
407	n/a	n/a	n/a	n/a	n/a	n/a	n/a	-
409	n/a	n/a	n/a	n/a	n/a	n/a	n/a	-
415	n/a	n/a	n/a	n/a	n/a	n/a	n/a	-
439*	n/a	n/a	n/a	n/a	n/a	n/a	n/a	-
450*	n/a	n/a	n/a	n/a	n/a	n/a	n/a	-
395	n/a	n/a	n/a	n/a	n/a	n/a	n/a	-
413	n/a	n/a	n/a	n/a	n/a	n/a	n/a	-
420	n/a	n/a	n/a	n/a	n/a	n/a	n/a	-
394								β8,9
418	n/a	n/a	n/a	n/a	n/a	n/a	n/a	-
392	n/a	n/a	n/a	n/a	n/a	n/a	n/a	-
406								β8,9
384								β3,5
393	n/a	n/a	n/a	n/a	n/a	n/a	n/a	-
443*	n/a	n/a	n/a	n/a	n/a	n/a	n/a	-
444*	n/a	n/a	n/a	n/a	n/a	n/a	n/a	-
448*	n/a	n/a	n/a	n/a	n/a	n/a	n/a	-
414								c4
447*								c3
387								c3
411	n/a	n/a	n/a	n/a	n/a	n/a	n/a	-
422								c3
425	n/a	n/a	n/a	n/a	n/a	n/a	n/a	-
436*								c4
438*								c4
446*	n/a	n/a	n/a	n/a	n/a	n/a	n/a	-
424								c3
440*	n/a	n/a	n/a	n/a	n/a	n/a	n/a	-
389								c3
433								c3
383	n/a	n/a	n/a	n/a	n/a	n/a	n/a	-
385								c/α
391	n/a	n/a	n/a	n/a	n/a	n/a	n/a	-
398	n/a	n/a	n/a	n/a	n/a	n/a	n/a	-
412	n/a	n/a	n/a	n/a	n/a	n/a	n/a	-
421	n/a	n/a	n/a	n/a	n/a	n/a	n/a	-
432	n/a	n/a	n/a	n/a	n/a	n/a	n/a	-
404	n/a	n/a	n/a	n/a	n/a	n/a	n/a	-

Specificity and Affinity (Biacore)						
**ISOBMii Ab Codes**	**hCG**	**hCGβ**	**hCGβcf**	**Epitope** Biacore	**Epitope** CLA + Biacore	**Epitope** final
403				β1-5	β1,2,4	β_1
435*				β1-5	β1,2,4	
382				β1-5	β1,2,4	β_2
388				β1-5	β1,2,4	
390				β1-5	β1,2,4	
402				β1-5	β1,2,4	
408				β1-5	β1-5	
416				β1-5	β1,2,4	
417				β1-5	β1,2,4	
426				β1-5	β1,2,4	
427				β1-5	β1,2,4	
449*				β1-5	β1,2,4	
396				β1-5	β1-5	β_3
399				β1-5	β3,5	
400				β1-5	β1-5	
401				β6-7	β6,7	
405				β1-5	β3,5	
423				β1-5	β3,5	
434*				β1-5	β1-5	
441*				β1-5	β1-5	
419				β1-5	β1,2,4	β_4
445*				β1–5	β1,2,4	
428 sheep				β1-5	β3,5	β_5
429 sheep				β1-5	β1,2,4	
430 sheep				β1-5	β3,5	
431 sheep				β1-5	β3,5	
442*				β1-5	β3,5	
410				β6,7	β6,7	β_6
437*				β1-5	β1-5	
386				β1-5	β1-5	β_7
397				β6,7	β6,7	
407				β6,7	β6,7	
409				β6,7	β6,7	
415				β6,7	β6,7	
439*				β6,7	β6,7	
450*				β8,9	β8,9	β_8
395				β8,9	β8,9	
413				β8,9	β8,9	
420				β8,9	β8,9	
394				β8,9	β8,9	β_9
418				β8,9	β8,9	
392				β	β	β
406				β8,9	β8,9	
384				βcf	β	βcf
393				βcf	βcf	
443*				β	β	
444*				βcf	βcf	
448*				βcf	βcf	
414				c/α	c4	c_1
447*				c/α	c3	
387				c/α	c3	c_2
411				c/α	c/α	
422				c/α	c3	
425				c/α	c/α	
436*				c/α	c4	
438*				c/α	c4	
446*				c/α	c/α	c_3
424				β8,9	β8,9/c3	c_4
440*				c/α	c/α	
389				?	?	c
433				c/α	c3	
383				c/α	c/α	α
385				c/α	c/α	
391				c/α	c/α	
398				β	β	
412				c/α	c/α	
421				c/α	c/α	
432				c/α	c/α	
404				c/α	c/α	α_6?

*reference antibodies | I.C. … intern/al control

Key (kD)
< 0.01 nM
0.01 - 0.1 nM
0.11 - 1 nM
1.1 - 10 nM
>10 nM
